# Supplementary material for: Modeling ocean distributions and abundances of natural- and hatchery-origin Chinook salmon stocks with integrated genetic and tagging data
Source: PeerJ. 2023 Nov 28;11:e16487. doi: 10.7717/peerj.16487 (PMC10691356; doi:10.7717/peerj.16487)
Supplement: Supplemental Information 1 [file peerj-11-16487-s001.docx]

Jensen et al. Supplement S1

**Overview**

This model description is derived from the model description provided in Supplement S1 from Shelton et al. (2021). We describe model components common to all models (i.e., *CWT-only, CWT+GSI,* and *CWT+GSI+Age)*, as well as components specific to only one or two models. Furthermore, we note where detailed descriptions of model processes are redundant with text from Supplement S1 in Shelton et al. (2021) and are therefore cut for brevity.

**Base model description for all models**

To estimate the seasonal abundance and distribution of fall-run Chinook salmon, hereafter CS, we simultaneously model the abundance and distribution of CS that leave rivers and enter the ocean in 5 of the 8 modeled ocean regions (Fig. 1) over 36 years (brood years 1977-2012). Calendar seasons, *s*, were defined as follows: spring (Mar-May), summer (Jun-Jul), fall (Aug-Oct) and winter (Nov-Feb). Compared to the model described in Shelton et al. 2021, we model ocean distribution for fewer and higher-resolution origin regions (i.e., stocks) over a smaller number of ocean regions for an expanded number of brood years. Our selected stocks were Central Valley fall-run (SFB), California Coast (CAC), Klamath River (KLT), and North California/South Oregon Coast (NCASOR); these stocks reflect distinct Evolutionarily Significant Units and distinct genetic reporting groups (Seeb et al., 2007; Clemento et al., 2014). We selected a smaller number of ocean regions due to limited available genetic stock identification (GSI) data north of WAC (Fig. 1) and to present a manageable case study to guide future integrated models that use both coded wire tag (CWT) and GSI data. Furthermore, the four selected stocks were very rarely detected north of the Washington state - Canada border (see Shelton et al., 2019, 2021), supporting the reduced number of ocean regions; more northerly distributed stocks would require GSI data coverage north of WAC (*Supplement S2*). Relative to Shelton et al. (2021), we increased the number of brood years to take advantage of the availability of more recent CWT recovery data (i.e., through 2018). We also changed the definition of calendar seasons for spring and winter to better match existing patterns of fishery harvest.

Our model for CS stocks tracks the abundance of fish from the spring of age 2 (defined as calendar year minus brood year, with age incrementing at the beginning of the fall season) to fall of age 6, encompassing 19 seasonal steps (Table S1.1). As conventions for describing the age of CS are confusing and vary regionally and by run type, we provide a table outlining our CS age classification used (Table S1.1).

Our focal stocks of CS enter the river and spawn during the fall (August to October). Eggs that are deposited in calendar year *c* hatch and spend much of their first year growing in freshwater and most enter saltwater as smolts during the spring or summer of the following year. At the beginning of fall in year *c+1*, they become age *y=*1 fish. In the fall of year *c+2* they become age *y=2*, etc. (Table S1.1). In general, we try to keep indexing as simple as possible by tracking populations in terms of the model age in seasons (*a*) with *a* = 1 corresponding to spring of calendar year 2, *a* = 5 corresponding to spring of age 2, up to *a* = 19 indicating fall of age *y* = 6. Unfortunately, some model components such as fishing mortality vary and are reported by calendar year and season, not model age, and so have appropriate indices to reflect this complexity (see below).

To generate estimates of the abundance of tagged CS from each of the 4 stocks in the 8 ocean regions, we need to understand six core processes: 1) the number of tagged fish entering the ocean from origin rivers (for the purposes of the estimation described in this supplement, only the abundances of tagged fish are needed); 2) the natural mortality of juvenile fish; 3) the natural mortality of adult fish; 4) ocean region-specific fishing mortality by age; 5) the spatial distribution of fish in the ocean; and 6) the age-specific loss of fish from the ocean due to maturation.

Distinct but related versions of this base model are described in detail in Shelton et al. (2019; 2021). We note places where the model structure has diverged from the Shelton et al. (2021) model. A full list of parameters and subscripts used in model description in Table S1.2.

**Additional model description for models incorporating GSI data (*CWT+GSI, CWT+GSI+Age*)**

For models including GSI data, in addition to tracking the abundance and distribution of CWT CS release groups, we now also track the abundance and distribution of CS from each stock in each year (including all hatchery- and natural-origin CS). These new groups allow us to connect model predictions of abundance and harvest to available GSI data (see *Expected GSI group catches and likelihoods for GSI mixture data*, below). For simplicity, we refer to CWT released fish as 'CWT release groups' or 'CWT releases' and the total releases from a given stock as "total release groups" or "total releases". We understand that natural production does not, in fact, involve the release of CS at any point, but we retain the nomenclature because of the data parallels with CWT information. We only tracked total release groups for broodyears 1992-2012 because available GSI data were first collected in 1998, and 1992 represents the earliest broodyear that could reasonably contribute to sampled catch in 1998.

For total releases, we only need to understand the following four core processes to generate estimates of the total abundance of CS from each of the 4 stocks in the 8 ocean regions: 1) the natural mortality of adult fish; 2) ocean region-specific fishing mortality by age; 3) the spatial distribution of fish in the ocean; and 4) the age-specific loss of fish from the ocean due to maturation. There is no data for most CS stocks on the number of individuals outmigrating from each stock and so we estimate the initial number of individuals for each total release group (i.e., starting at age *a* = 1) within the model (see below); there is no need or ability to account for the number of fish entering the ocean or juvenile mortality before age *a* = 1.

**Process model description**

*Defining releases and early life-history for all models*

We are interested in tracking the number and distribution of each CWT CS release for the entirety of its life cycle. We denote the initial number of CS from release group $i$ as $N_{0,i}$. Each release group is associated with a particular origin region, brood stock, brood year, and release year and thus may include multiple individual tag codes reported in the CWT database (see Supplement S2). Because we use hatchery releases of tagged fish, $N_{0,i}$ are treated as known without error. Let $\phi_{i}$ be the estimated juvenile mortality rate for fish from release group $i$. “Juvenile” here includes the period between release and the start of the model (which varies between one and thirteen months, depending on release month) and includes both time spent in freshwater and the marine environment (although we note some releases are trucked downstream and spend little or no time in freshwater after release; Sturrock et al., 2019). The abundance of a release at the end of the juvenile period is then$N_{i,0}e^{{-\phi}_{i}}$. This structure allows for differences in juvenile survival for fish arising from different rivers or hatcheries within a single origin region. We model juvenile mortality hierarchically, $\phi_{i} \sim LogNormal(\underline{log(\phi)},\tau_{\phi})$, with $\underline{log(\phi)}$ the among stock median and $\tau_{\phi}$ controlling the dispersion of mortality rates on the log scale.

*Additional release and early life-history details for models including GSI data*

We were also interested in tracking the number and distribution of each total CS release group *k* for each stock (i.e., one release group per stock and year, representing the total number fish, both tagged and untagged). Due to a lack of outmigrant estimates at the stock-level, we do not have equivalent data for the CWT and total release groups. We define N*_k,0_* as the number of CS present at the beginning of timestep 1 (equivalent to the end of the juvenile period) We estimate total release abundance at the end of the juvenile period as the sum of expected CWT-tagged fish abundance (corresponding to the same stock, year, and age as the aggregate release), after accounting for juvenile mortality using $\phi i$, and the number of non-CWT-tagged fish (i.e., estimated as a latent variable). So the

$$N_{k0}=N_{kW}+\sum_{i\in k} N_{i0}e^{-\phi_{i}} (S1.1)$$

where $N_{kW}$ represents the number of CS that are not CWT-tagged from stock-year combination *k.* We constrain $N_{kW}$ to be positive with this structure.

This constraint facilitates model convergence by reducing the occurrence of unrealistically low abundance estimates.

*Population dynamics structure for all models*

In each of the 8 ocean regions, there is natural and fisheries related mortality of CS, so for the winter, spring, and summer, the number of CS from release group *i* (or *k,* not shown in Eq. S1.1) in ocean region *l* at the end of model age *a* is

$N_{i,a+1,l}= N_{i,a}\theta_{r,l,s,c}e^{{-M}_{a}-\sum_{g} F_{a,s,c,l,g}-\sum_{g} U_{a,s,c,l,g}}$ ($S1.$2)

where $M_{a}$is the density-independent natural mortality, $F_{a,s,c,l,g}$ and $U_{a,s,c,l,g}$ represent the fishing mortality rate for retained and not retained salmon, respectively, for model age *a*, in season *s,* calendar year *c,* ocean region *l,* and gear type *g.* The parameter $\theta_{r,l,s,c}$ is the proportion of fish from origin (or stock) *r*, present in ocean region *l,* at the beginning of season *s* in calendar year *c* (see *Ocean Distribution Model* below)*.* Note that *M* and *F* do not depend on stock (*r*) nor release group (*i* or *k*) because we assume age-specific mortality rates are identical across stocks after the juvenile stage, as is fisheries vulnerability (see *Vulnerability to fisheries* below, although future elaborations of this model might allow for departures from these assumptions). This formulation assumes that fish from the same stock, but potentially different rivers or hatcheries, have identical ocean distributions and these ocean distributions are the same for CS of different ages. By mapping tagged CS release groups *i* and overall CS groups *k* onto their common stock *r*, the integrated model shares information and enables the joint estimation of ocean distribution.

For CS, spawning adults leave the ocean and return to the freshwater to spawn. We model spawning as a proportion of fish leaving the ocean at fraction $f_{r}$ of the time during the fall season (0 < $f_{r}$ < 1). We use a leaving ocean date of September 1 ($f_{r}$ = 0.33) for all runs in this model but allow for variation in run timing among stocks in the future. Let $\delta_{r,l,y}$ be proportion of fish from stock *r*, in ocean region *l,* leaving the ocean to spawn and $D_{i,y}$ (below) be the total number of fish from release *i* leaving the ocean and entering the spawning rivers in spawning year *y*. We present equations S1.3-S1.5 in terms of *i* but note the same processes apply to total stock release groups *k*.

$D_{i,y}= \sum_{l=1}^{L} \left( N_{i,a}\theta_{r,l,s,c}{\delta_{r,l,y}e}^{{-f_{r}(M}_{a}+\sum_{g} F_{a,s,c,l,g}+\sum_{g} U_{a,s,c,l,g})} \right)$ ($S1.$3)

The number of fish remaining in the ocean in each region at the end of the fall is then

$N_{i,a+1,l}=N_{i,a}\theta_{r,l,s,c}(1-{\delta_{r,l,y})e}^{{-M}_{a}-\sum_{g} F_{a,s,c,l,g}-\sum_{g} U_{a,s,c,l,g}}$ ($S1.$4)

The total number of fish for each stock and age present along the entire coast is a latent variable in the model. We allow for process error that acts on the entire population,

$$N_{i,a}=e^{\omega_{i,a}}\sum_{l=1}^{8} N_{i,a,l} (S1.5)$$

where $\omega_{i,a}\sim N\left( 0,M_{a} \right)$. This process variability represents population drivers not included in our model that are experienced by all stocks in the ocean and serves to help account for errors of model mis-specification and additional unmodeled processes. We constrain the process error to have a standard deviation equivalent to the assumed age-specific natural mortality to avoid large swings in abundance over the life cycle. In an ideal world, our model would include all of the important aspects of salmon biology and process error would not be needed.

*Population dynamics structure for models including GSI data*

To improve model convergence in estimates of process error and, subsequently, abundance, we constrained the process error to be identical for every release (i.e., either CWT or total) associated with the same stock and broodyear, based on the implicit assumption that effects of population drivers not included in our model are shared by releases from the same stock and broodyear.

*Ocean distribution for all models*

CS are dispersed across the ocean in a way that varies seasonally. We assign $\theta_{r,l,s}$ to be the proportion of fish from origin region *r*, present in ocean region *l,* at the beginning of season *s.* In contrast to methods in Shelton et al. (2021), we removed year to year variability in distribution to simplify model estimation with limited data availability for selected stocks. We define $\theta_{r,l,s}=\frac{exp[\xi_{r,l,s}]}{\sum_{l} exp[\xi_{r,l,s}]}$; the parameters $\xi_{r,l,s}$ determines the distribution of fish for each stock and season. Across ocean regions, the proportions must sum to one because these represent the entire ocean extent:$\sum_{l} \theta_{r,l,s}=1$.

Since fisheries activity and fisheries recoveries are spatio-temporally patchy, and ocean biological distributions of CS are assumed to be smooth – adjacent ocean regions are more similar in abundance than distant regions, on average - we used a dimension reduction technique known as predictive process modeling (Banerjee et al., 2008, Finley et al., 2009, Shelton et al., 2014, 2019, 2021) to calculate $\xi_{r,l,s}.$ In short, this approach is analogous to a spline and uses fewer parameters to estimate the spatial distribution by imposing smoothness constraints on the values of $\xi_{r,l,s}$; adjacent ocean regions will have more similar values than distant regions. Specifically, we ordered the 8 ocean regions in our model domain (Fig. 1) from south to north and numbered them 1 to 8. We then introduced a set of 5 evenly spaced knot points from regions 0.75 to 8.25 for the summer, fall, and winter and spring season (knot locations can also be driven by spatial coordinates, but we assume equal area and distance between regions along the coast).

For each combination of stock and season, we estimate a latent parameter at the knot locations (though these random effects vary by stock and season, the knot locations are shared). From the value estimated at each knot, we used a Gaussian covariance function and the ordinary kriging projection to then project the latent variable, $\xi_{r,l,s}$ at each ocean region corresponding to the integer values (i.e., *l* = 1, 2, …,8). This results in a spatially smooth set of values for $\xi$ for each stock and season. To make the projection, we need to define two additional parameters beyond the value at each knot: 1) a smoothing parameter $\pi_{s}$ which controls how each knot contributes to the values at each region; and 2) a variance parameter, $\varpi$, which controls the overall scale of variability in $\xi$**.** We estimate two $\pi_{s}$, one for the winter-spring distribution ($\pi_{s=win:spr}$) and one for the summer and fall seasons ($\pi_{s=sum:fal})$. We use a fixed value of $\varpi=1.$ As in previous versions of this model (Shelton et al., 2019, 2021), due to limited fishing effort and recoveries during the winter months, we assume the spring and winter distributions are identical.

*Maturation and spawning migration, fishing mortality, vulnerability, and natural mortality for all models*

The vast majority of these components have not changed from the model presented in Shelton et al. (2021). As a result, we refer interested parties to that publication for details on the parameterization of these model components (see their Supplement S1, pages 6-10).

However, we updated the discrete region numbering systems, as applied in calculating the proportion of mature fish leaving a particular region of the ocean, for the four focal stocks to reflect the new stock structures in this case study. These discrete region numbers are provided in Table S1.3; we note that presented results include a deviation from this table for CWT releases from NCASOR only, such that the following numbers are applied for regions MONT-WAC: 4, 3, 2, 1, 1, 2, 3, 4. We expect the impacts of this deviation to be negligible.

**Observation model description**

*Expected landed catches for all models*

We lack direct, fisheries independent surveys of CS in the ocean, but CS are caught coastwide during a range of fisheries. We used spatio-temporally explicit recovery data from either one sampling method (CWTs, for the *CWT-only* model) or two sampling methods (CWTs and GSI, for models including GSI data), as well as multiple fishing gear types (i.e., commercial troll, recreational hook and line, and pelagic hake trawl bycatch). We also used estimates of total fishery landings for commercial troll and recreational hook and line gear types in our analysis. Specifically, we compared observed recovery and landings data to expected landed catch values, or latent states derived from expected landed catch, to estimate model parameters. For seasons without spawning migrations, the expected landed catch for each release group, age, region, and gear type follows the Baranov catch equation (Baranov, 1918; Beverton & Holt, 1957). Here we provide expected catch equations for CWT release groups *i* but note the same equations are applied to overall stock release groups *k*:

$$\mu_{1,i,a,l,g}=\frac{F_{a,s,c,l,g}}{M_{a}+F_{total}}N_{i,a}\theta_{r,l,s}\left\{ 1-exp\left[ -\left( M_{a}+F_{total} \right) \right] \right\} (S1.6)$$

where $F_{total}=\sum_{g} F_{a,s,c,l,g}+\sum_{g} U_{a,s,c,l,g}$. For the season when spawning migrations occur, let $N_{i,a,l,S}$ be the number of CS remaining in the ocean in region *l* at the end of the fall (S1.4) (i.e., after spawning fish enter freshwater at fraction $f_{i}$ of the time through the season). Then expected catch for the entire fall season in a region is

$$\mu_{1,i,a,l,g}=\frac{F_{a,s,c,l,g}}{M_{a}+F_{total}}N_{i,a}\theta_{r,l,s}\left\{ 1-exp\left[ -f_{i}\left( M_{a}+F_{total} \right) \right] \right\}+\frac{F_{a,s,c,l,g}}{M_{a}+F_{total}}N_{i,a,l,S}\left\{ 1 -exp\left[ -(1-f_{i})\left( M_{a}+F_{total} \right) \right] \right\}$$

($S1.7)$

*Expected and observed CWT recoveries for all models*

Estimates of parameters rely in part on comparing expected catches of CWT release groups (i.e., $\mu_{i,a,l,g}$) to the observed catches across all releases, times, and regions, and finding sets of parameters that minimize the distance between observed and expected catches. For each release group, we queried the RMIS database to extract all CWT recovered and assigned each catch record to a fishing gear, region, and season. Each recovery has an associated estimated number of fish it represents. The estimated number ($C_{i,a,l,g}$) is a deterministic expansion of the actual observed number of fish ($O_{j,a,l,g};$most commonly 1) by the fraction of the fishery catch sampled for CWT ($\varrho_{j,l,c,s,g};$generally between about 10% and 50%; Supplement S2). We summed across the estimated number of fish from each individual CWT tag code *j* that make up each release in our model; recall that what we are tracking in release group $i$ is a combination of individual CWT tag groups released from a single hatchery, brood stock, brood year, and release year. We use $O_{j,a,l,g}$ to represent the observed number of fish from tag code *j*, and $\varrho_{j,l,c,s,g}$ to represent the sampling fraction associated with $O$. The expanded total landed catch, $C_{i,a,l,g}$, for each release group is

$$C_{i,a,l,g}=\sum_{j} O_{j,a,l,g}\frac{1}{\varrho_{j,l,c,s,g}} (S1.8)$$

We use $C_{i,a,l,g}$ in model estimation because it is comparable to previous uses of CWT recovery data (e.g., Weitkamp, 2010; Satterthwaite et al., 2013; Kilduff et al., 2014,) and it lessens the computational burden of the model, moving from millions of observations required from tracking *O* for each CWT tag group to tens or hundreds of thousands with *C* for each aggregated release group*.* To accommodate this aggregation, we use a single fixed value of $\varrho_{l,c,s,g}$ for all observations in a season-year-ocean region-gear combination. In some cases, there is a small amount of variation within each stratum in $\varrho$ because a single season includes multiple reporting periods, and our ocean regions could be subdivided by samplers. When this occurred, we used the median value. This parameter is instrumental in determining and propagating uncertainty in catches (see next section).

In Shelton et al. (2021) we derived a likelihood to use with CWT recovery data. We summarize the results of that derivation here, but do not recreate it. In summary, we incorporate a likelihood for the probability of observing at least one CWT fish from release as,

$$G_{i,a,l,g}\sim Bernoulli\left( 1-\left( \frac{\beta_{i,a,l,g}\varrho_{l,c,s,g}^{-1}}{\beta_{i,a,l,g}\varrho_{l,c,s,g}^{-1}+1} \right)^{\alpha_{i,a,l,g}} \right) (S1.9)$$

where the parameters $\alpha_{i,a,l,g}$ and $\beta_{i,a,l,g}$ are a function of the expected catch ($\mu_{1,i,a,l,g}$), the sampling fraction ($\varrho_{l,c,s,g}$) and a gear-specific parameter $\sigma_{1,g}$ that controls the amount of overdispersion in the catch.

$\alpha_{i,a,l,g}=\frac{1}{{\sigma_{1,g}}^{2}}, \beta_{i,a,l,g}=\frac{\alpha_{i,a,l,g}}{\mu_{1,i,a,l,g}}$ (S1.10)

Second, for cases where we observe non-zero CWT recoveries, we model the expanded number of CWT observed as a gamma distribution

$$C_{i,a,l,g}\sim Gamma\left( \alpha_{C_{i,l,s,g}},\beta_{C_{i,l,s,g}} \right), if C_{i,l,s,g}>0 (S1.11)$$

whose parameters $\alpha_{C_{i,l,s,g}},\beta_{C_{i,l,s,g}}$are also functions of $\mu_{i,a,l,g}$, $\sigma_{1,g}$, and $\varrho_{l,c,s,g}$ but do not have a simple, compact form (see Shelton et al. 2021 for details). For this model, both the probability of occurrence and positive components are connected logically to three understandable quantities: the expected catch for each release group in each region, age, and gear type stratum, the stochastic variation in catch associated with each gear type, and the sampling fraction. This model allows for the possibility of observing zero fish from a stratum even when non-zero fish from that release were actually caught; decreases in the sampling fraction increase the probability of observing zero fish.

*Observation models and data for Chinook salmon escapement proportions, for all models*

Another component of the model accounts for the CS that leave the ocean and return to their origin river or hatchery and complete their life cycle. Ideally, we would have a likelihood component corresponding to the observed fish in rivers and hatcheries for each release group. Unfortunately, examination of the RMIS database revealed problems in the freshwater recovery data; we identified some individual tag groups from throughout the study region with many ocean recoveries but near zero freshwater recoveries. Following Shelton et al. (2019, 2021), we elected to incorporate only information about the relative occurrence of different age CS in freshwater recoveries, not the actual expanded numbers of total observed freshwater recoveries.

Thus, for a given release group we calculated the proportion of fish from a given release group that were observed in freshwater for ages 2 to 6. We only include releases with at least 50 individuals observed in freshwater. Across all release groups within a stock, we then calculated an average proportion of age *y* fish observed in freshwater $p_{1,r,y}$ for each origin region. This approach assumes that the observed proportion of fish from a given release each age is accurate but ignores absolute abundance estimates. As these proportions must sum to 1, we use $p_{1,r,y}$ to inform the parameters of a Dirichlet likelihood; the predicted proportion of a CS release group in freshwater is

$$p_{1,r,y}\sim Dirichlet\left( n\frac{D_{i,y}}{\sum_{y} D_{i,y}} \right) (S1.12)$$

where $n$is the effective sample size and scales the estimated proportion of fish in freshwater to reasonable parameter values for the Dirichlet distribution (Table S1.2). Here we provide the predicted proportion equation for CWT release groups *i* but note the same equations also are applied to overall stock release groups *k*. Due to insufficient numbers of observed freshwater CWT recoveries for the California Coast stock (CAC), we assumed age-based proportions of CAC CS release groups in freshwater were equivalent to those estimated for the Klamath stock (KLT). We use *n = 100* for all stocks but CAC, which was assigned *n* = *10* to reflect the borrowing of information from KLT*,* in the results shown. Therefore, we assume that the releases within a region can vary among releases and across years in their return proportions, but they are constrained to be broadly similar to the average proportion for each region.

*Observation models and data for Chinook salmon run sizes for models including GSI data*

To constrain estimated abundances for total release groups *k* to biologically feasible values, given the absence of freshwater release abundances, we compare $D_{k,y}$ values (i.e., the total number of fish from release *k* leaving the ocean and entering the spawning rivers in spawning year *y*) to independent estimates of stock-specific run size. Specifically, we sum all $D_{k,y}$ values corresponding to calendar year *c* and stock *r* to produce model predictions of stock run size $\mu_{2,c,r}$. We compare independent (“observed”) estimates of escapement values, $X_{c,r}$, to expected values,

$$X_{c,r}\sim Lognormal\left( log \left( \mu_{2,c,r} \right) ,\sigma_{2,c,r} \right) (S1.13)$$

where $\sigma_{2,c,r}$ is the log-scale standard deviation of the distribution. We conducted these comparisons for years 1998-2014 and for all stocks. We selected these years based on the availability of GSI data and modeled stock release groups, as we can only compare predicted and “observed” run sizes for calendar years *c* in which release groups representing all possible ages (2-6) are modeled.

We obtained run size data for SFB, CAC, KLT, and NCASOR groups by compiling contemporary and historical datasets on a stock-by-stock basis (Anderson and Ward, 2016; CDFW unpublished data, 2019, 2021, 2022; ODFW, 2013; PFMC, 2020; USFWS, 1960; Zach Larson & Associates, 2013). For SFB, we summed escapement estimates for fall-run, late fall-run, and Feather River Hatchery spring-run fish, in addition to fall-run harvest estimates on the Sacramento River, to obtain a measure of total run size; fall-run, late fall-run, and Feather River Hatchery spring-run fish are genetically highly similar in the Central Valley fall-run reporting group (Seeb et al., 2007; Clemento et al., 2014). We summed spring- and fall-run run size estimates to obtain total KLT run size again due to the inability to separate run timing groups using GSI alone. Obtaining total run size estimates for some stocks (i.e., CAC and NCASOR) proved challenging because these stocks include numerous small and relatively isolated coastal watersheds with inconsistent historical survey and estimation efforts. Depending on the availability of more-or-less complete run size estimates for each stock and year, we fixed values of $\sigma_{2,c,r}$ to either 0.1 (complete estimate) or 0.2 (incomplete) (see *Supplement S2*). We selected these values based on visual examinations of variability corresponding to these values and professional judgment. We also note that a recent rebuilding plan for Pacific salmon assumed a CV of 20% for escapement estimates (O’Farrell & Satterthwaite, 2021).

*Expected GSI group catches and likelihoods for GSI mixture data, for models including GSI data*

As with our comparison of expected and observed catches of CWT release groups *i*, we conduct similar comparisons of expected catches of total stock release groups *k* to GSI mixture data to find sets of parameters that minimize the distance between observed and expected values. We obtained GSI data from past sampling of recreational hook-and-line harvest in California (Satterthwaite et al. 2015) and sampling of commercial troll harvest in California, Oregon, and Washington (Satterthwaite et al., 2014; Bellinger et al., 2015). Although GSI data spanned 1998-2018, we necessarily restricted the range to 1998-2014. If we were to include years 2015 or later, we would need to model brood years beyond 2012, and we lacked necessary CWT data (post-2018) to model the entire lifespan of these later brood years. We excluded GSI data for select regions, seasons, and years for which the number of sampled fish was less than 10.

Unlike CWT data, GSI data only provide information on the relative proportions of genetic reporting groups within a group of sampled fish (e.g., 11% of 200 sampled fish in region *l,* season *s*, year *c* are assigned to SFB); it is not possible to identify particular brood year components within a stock. For a specific *l/s/c* combination, specific GSI data are the total number of sampled fish, $Y_{l,s,c,g}$, and the number of fish assigned to each reporting group (or stock *r*), $y_{l,s,c,r,g}$. We note that $Y_{l,s,c,g}$ is necessarily an integer but $y_{l,s,c,r,g}$ can be any positive real number (or 0), as we sum individual assignment probabilities to each stock *r* across all sampled fish to obtain $y_{l,s,c,r,g}$ (see also Jensen et al., 2022).

The structure of these data presents several challenges for integration into the existing model. First, the sum of $y_{l,s,c,r,g}$ across modeled *r* for a given *l*, *s*, *c*, and *g* is not necessarily equal to $Y_{l,s,c,g}$ if unmodeled stocks are present in sampled fish. Second, observed $y_{l,s,c,r,g}$ potentially encompass fish from multiple brood years *k* from stock *r*, preventing us from directly comparing GSI observations to predicted catch from specific release groups. Finally, observations ($y_{l,s,c,r,g}$) can take on a value of zero or be positive, preventing us from using standard multivariate likelihoods in model estimation. We use a newly developed mixture model (Jensen et al., 2022) that can account for challenges of these data.

The first challenge is that we observe individual fish in the GSI data that are assigned to stocks that are not of focal interest. Therefore, we must first define an expected proportion of the catch that is from our focal groups. First, we sum $\mu_{1,k,a,l,g}$ from stock *r* across all release and age combinations (indices *k* and *a*) that contribute to catch from region *l,* season *s*, year *c*, and gear *g* from stock *r*, yielding the new expected catch $\mu_{1,l,s,c,r,g}$ for each stock. We also obtain the observed number of sampled fish from modeled, or focal, stocks, $y_{l,s,c,F,g}$, by summing $y_{l,s,c,r,g}$ across all *r*, and estimate the proportion of focal stocks,$p_{2,l,s,c,F,g}$, in the observed number of fish $Y_{l,s,c,g}$,

$$y_{l,s,c,F,g}\sim Binomial\left( Y_{l,s,c,g},p_{2,l,s,c,F,g} \right) (S1.14)$$

where $y_{l,s,c,F,g}$ has been rounded to the nearest integer. We similarly obtain total expected catch of all focal stocks, $\mu_{1,l,s,c,F,g}$, by summing $\mu_{1,l,s,c,r,g}$ across all *r.* We calculate expected catch across all stocks (i.e., focal and non-focal), $\mu_{1,l,s,c,g}$, by expanding the focal stocks by their estimated proportion,

$$\mu_{1,l,s,c,g}=\mu_{1,l,s,c,F,g}/p_{2,l,s,c,F,g} (S1.15)$$

Using $p_{2,l,s,c,F,g}$, $\mu_{1,l,s,c,r,g}$, and $\mu_{1,l,s,c,g}$, we calculate the expected proportion contribution of each stock *r* ($p_{2,l,s,c,r,g}$), as well as the proportional contribution of non-focal stocks ($p_{2,l,s,c,NF,g}$), to expected harvest where,

$$p_{2,l,s,c,r,g}=\mu_{1,l,s,c,r,g}/\mu_{1,l,s,c,g} (S1.16)$$

$$p_{2,l,s,c,NF,g}=1-p_{2,l,s,c,F,g} (S1.17)$$

Finally, we compare the expected proportional contributions of each stock *r* to GSI data $y_{l,s,c,r,g}$ and $Y_{l,s,c,g}$ using a zero-and-one inflated Dirichlet regression model with estimated overdispersion $\phi_{GSI}$ (Jensen et al., 2022); proportional contribution of non-focal stocks are necessary only to calculate component probabilities in the mixture model. Additional model details and worked examples of model implementation can be found in Jensen et al. (2022) The overdispersion term allows the model to weight the importance of GSI data relative to overall model likelihoods; lack of consistent sampling metadata associated with GSI observations prevented more empirical means of data weighting.

*Observation models and data for Chinook salmon total landings, for models including GSI data*

To ensure the model does not estimate unrealistic values for overall stock catches, as a function of potentially unattainable release group abundances and fishery mortality terms, we compare expected catch across all stocks to independent estimates of landings. We obtained landings estimates for commercial troll and recreational hook-and-line fisheries, summarized primarily by port, month, and year, from PFMC (2019a). We aggregated landings from each gear type by season and ocean region to obtain observed total landings, $Z_{l,s,c,g}$ (see *Supplement S2* for details). We compared observed landings to expected catches for all seasons and regions with positive observed landings from 1998-2014:

$$Z_{l,s,c,g}\sim Normal(\mu_{1,l,s,c,g},\mu_{1,l,s,c,g}\sigma_{3}) (S1.18)$$

where $\sigma_{3}$ is the coefficient of variation for $\mu_{1,l,s,c,g}$. We fix $\sigma_{3}$ to 0.1 based on professional judgment and the recent assumed 10% CV in harvest rate implementation error (O’Farrell and Satterthwaite, 2021).

Calculating $\mu_{1,l,s,c,g}$ for every region, season, year, and gear-type with non-zero observed landings is challenged by availability of GSI data necessary to calculate $p_{2,l,s,c,F,g}$. Therefore, in addition to estimating $p_{2,l,s,c,F,g}$ in the presence of GSI data with the binomial likelihood, we specify $p_{2,l,s,c,F,g}$ hierarchically so that

$$logit(p_{2,l,s,c,F,g})\sim Normal\left( p_{2,l,F},\sigma_{4} \right) (S1.19)$$

in which $p_{2,l,F}$ is the expected logit-transformed proportion of focal stocks in landings by region and $\sigma_{4}$ is the standard deviation around proportions. We applied a logit transformation to $p_{2,l,s,c,F,g}$ to ensure all estimated values are bounded by [0,1]. The hierarchical specification allowed the model to estimate $p_{2,l,s,c,F,g}$ in the absence of corresponding GSI data. We note that we assumed expected proportions of focal stocks in landings in the COL region were equal to those in the WAC region due to a paucity of GSI data from COL.

*Exploration of synthetic age-structure data for the* GSI+CWT+Age *model*

Based on observed challenges in achieving model convergence for estimates of total release group abundances, we added an exploratory, additional age-structure constraint to the model. We hypothesized that the lack of age-structure data in both marine landings and freshwater escapement allowed similar total stock abundances to be achieved with multiple combinations of total-release specific abundances. For example, a total estimated stock abundance of 100,000 fish (i.e., estimated based on other described data inputs including total landings, stock-specific run sizes, and GSI data) could be achieved with 90,000 age-2, 5,000 age-3, 3,000 age-4, and 2,000 age-5 fish; alternatively, the same stock abundance could be achieved with 20,000 age-2, 70,000 age-3, 5,000 age-4, and 5,000 age-5 fish. With no age-structure data in place, the model is allowed considerable flexibility in estimating total release-specific abundances over time.

We added an exploratory age-structure constraint to the model by providing fabricated samples of ages from marine landings as part of a new multinomial likelihood. Specifically, for a given season, year, and stock, we provide counts of ages, generated from the product of fixed age proportions (i.e., either ages 2-5 or 3-5, depending on season) and a specified number of sampled fish. In spring and summer, we specify proportions of ages including 2-5; in fall and winter, we specify proportions for ages 3-5 because age 2 fish, according to the current model structure, are only tracked in spring and summer (age 2 fish become age 3 fish in fall, and the next release group is not tracked until spring). These counts are then applied to a multinomial likelihood, in which the multinomial probabilities are calculated using model estimates of release-specific (and therefore age-specific) fish abundances (i.e., for ages 2-5). This is formalized in the equation below, in which $p_{3,.,s,c,r}$ is a vector of proportions $p_{3,a,s,c,r}$, which represent the proportional contributions of release age-*a* fish to the total abundance of stock *r* (either encompassing ages 2-5 or 3-5, depending on the season) in season *s* and calendar year *c*, and $A_{.,s,c,r}$ is a vector of sampled fish assigned to release ages *a,* ranging from years 2-5, for stock *r* in season *s* and calendar year *c*.

$A_{.,s,c,r}\sim Multinomial\left( p_{3,.,s,c,r} \right) (S1.20)$

Proportions $p_{3,a,s,c,r}$ are calculated using model estimates of release- and stock-specific total release abundances.

With this new age-structure, our simulated data of aged fish, generated with an expected proportional contribution of different ages to stock abundance, generally constrain estimates of release-specific abundances to conform to our expected age contributions. We restricted our consideration of age constraints to ages 2-5 due to a dearth of age 6 fish in the cohort reconstructions used to inform our specified data.

We based our proportions of fish aged 2, 3, 4, 5 on the reconstructed total Klamath River Fall Chinook ocean abundance at age estimated by the ocean fisheries management models used to produce the summary statistics reported in PFMC (2021). For spring and summer, we specify estimated age 2, 3, 4, and 5 proportions of 0.62, 0.29, 0.08, and 0.01, respectively, for all stocks but SFB. For SFB, we specified estimated proportions of 0.65, 0.33, 0.015, and 0.005. We expected SFB to have an age distribution skewed towards younger ages based on past observations of earlier maturation rates (Satterthwaite et al., 2017). For fall and winter, we specify age 3, 4, and 5 proportions of 0.76, 0.21, and 0.03 for all stocks but SFB; for SFB, we specify age 3, 4, and 5 proportions of 0.89, 0.10, and 0.01. We applied a total sample size of 200 aged fish for each season, year, and stock, based on recommended sample sizes for both stock composition using GSI and age composition using scale aging (Allen-Moran et al., 2013; Satterthwaite et al. 2013), as well as observed scale reading counts for assessment of Klamath fall-run Chinook salmon age composition (Klamath River Technical Team, 2021).

We acknowledge that some of these specified data would be challenging to collect at our specified sample sizes. For example, many hundreds or thousands of fish would have to be sampled to obtain 200 identified CAC fish for aging purposes, based on observed contributions of CAC fish to total catch from available GSI data. In reality, age structure data is more readily collected for abundant stocks like SFB than rarer stocks like CAC.

Table S1.1. Age conventions for fall Chinook salmon used in this paper.

|  |  |  | **Fall Chinook Salmon** | | |
| --- | --- | --- | --- | --- | --- |
| **Calendar Year (c)** | **Calendar Month** | **Model Month (m)** | **Season (s)** | **Model Age (a)** | **Spawner Age (y)** |
| Brood Year (BY) | September | pre-model | Fall | pre-model | 0 |
|  | October |  |  |  |  |
|  | November |  | Winter |  |  |
|  | December |  |  |  |  |
| BY+1 | January |  |  |  |  |
|  | February |  |  |  |  |
|  | March |  | Spring |  |  |
|  | April |  |  |  |  |
|  | May |  |  |  |  |
|  | June |  | Summer |  |  |
|  | July |  |  |  |  |
|  | August |  | Fall |  | 1 |
|  | September |  |  |  |  |
|  | October |  |  |  |  |
|  | November |  | Winter |  |  |
|  | December |  |  |  |  |
| BY+2 | January |  |  |  |  |
|  | February |  |  |  |  |
|  | March | 1 | Spring | 1 |  |
|  | April | 2 |  |  |  |
|  | May | 3 |  |  |  |
|  | June | 4 | Summer | 2 |  |
|  | July | 5 |  |  |  |
|  | August | 6 | Fall | 3 | 2 |
|  | September | 7 |  |  |  |
|  | October | 8 |  |  |  |
|  | November | 9 | Winter | 4 |  |
|  | December | 10 |  |  |  |
| BY+3 | January | 11 |  |  |  |
|  | February | 12 |  |  |  |
|  | March | 13 | Spring | 5 |  |
|  | April | 14 |  |  |  |
|  | May | 15 |  |  |  |
|  | June | 16 | Summer | 6 |  |
|  | July | 17 |  |  |  |
|  | August | 18 | Fall | 7 | 3 |
|  | September | 19 |  |  |  |
|  | October | 20 |  |  |  |
|  | November | 21 | Winter | 8 |  |
|  | December | 22 |  |  |  |
| BY+4 | January | 23 |  |  |  |
|  | February | 24 |  |  |  |
|  | March | 25 | Spring | 9 |  |
|  | April | 26 |  |  |  |
|  | May | 27 |  |  |  |
|  | June | 28 | Summer | 10 |  |
|  | July | 29 |  |  |  |
|  | August | 30 | Fall | 11 | 4 |
|  | September | 31 |  |  |  |
|  | October | 32 |  |  |  |
|  | November | 33 | Winter | 12 |  |
|  | December | 34 |  |  |  |
| BY+5 | January | 35 |  |  |  |
|  | February | 36 |  |  |  |
|  | March | 37 | Spring | 13 |  |
|  | April | 38 |  |  |  |
|  | May | 39 |  |  |  |
|  | June | 40 | Summer | 14 |  |
|  | July | 41 |  |  |  |
|  | August | 42 | Fall | 15 | 5 |
|  | September | 43 |  |  |  |
|  | October | 44 |  |  |  |
|  | November | 45 | Winter | 16 |  |
|  | December | 46 |  |  |  |
| BY+6 | January | 47 |  |  |  |
|  | February | 48 |  |  |  |
|  | March | 49 | Spring | 17 |  |
|  | April | 50 |  |  |  |
|  | May | 51 |  |  |  |
|  | June | 52 | Summer | 18 |  |
|  | July | 53 |  |  |  |
|  | August | 54 | Fall | 19 | 6 |
|  | September | 55 |  |  |  |
|  | October | 56 |  |  |  |

Table S1.2. Parameter definitions, descriptions, and prior distributions.

| **Data** | |  | ***Prior*** |
| --- | --- | --- | --- |
|  | $N_{0,i}$ | Initial number of smolts released by a hatchery in release group *i* | *N/A* |
|  | $\varrho_{l,c,s,g}$ | Fraction of catch sampled for ocean region *l,* calendar year *c,* season *s*, and gear type *g* | *N/A* |
|  | $E_{c,l,sg}$ | Fisheries effort data for calendar year *c,* season *s*, ocean region *l,* and gear type *g* | *N/A* |
|  | $R_{l,c,s,g}$ | Minimum retention size in inches for each region, calendar year, season, and gear type | *N/A* |
|  | $C_{i,c,l,s,g}$ | Expanded ocean catch for release $i$*,* calendar year *c,* ocean region *l,* season *s,* and gear type *g* | *N/A* |
|  | $G_{i,c,l,s,g}$ | Bernoulli random variable taking on a value of 1 if $C_{i,c,l,s,g}>0$ and 0 otherwise | *N/A* |
|  | $X_{c,r}$ | Independent estimates of run size | *N/A* |
|  | $y_{l,s,c,F,g}$ | Number of GSI sampled fish assigned to stock *r* | *N/A* |
|  | $Y_{l,s,c,g}$ | Number of GSI sampled fish | *N/A* |
|  | $Z_{c,l,s,g}$ | Independent estimates of landings | *N/A* |
|  | $A_{r,a,s,c}$ | Number of fish with known age *a* for stock *r* in season *s* and year *c* | *N/A* |
| **Ocean Distribution** | | |  |
|  | $\xi_{r,l,s}$ | Parameters defining the fish distribution for each origin region | Function of $\xi_{r,s,knot}$ $\varrho, and \varpi$ |
|  | $\theta_{r,l,s}$ | Proportion of surviving population from release region *r* in ocean region *l* for season *s* | Function of $\xi_{r,l,s}$ |
|  | $\pi_{s}$ | Spatial smoothing parameter | $\pi_{s}\sim$*Gamma(3,1)* |
|  | $\varpi$ | Spatial standard deviation parameter | *Fixed,* $\varpi=3$ |
|  | $\xi_{r,s,knot}$ | Knot values that are used in predictive process smoother. | *Normal(0,*$\varpi$ *^2^)* |
| **Juvenile Mortality** | | |  |
|  | $\underline{log(\phi)}$ | Hierarchical mean mortality rate across all releases | $\underline{log(\phi)}\sim Normal(loglog \left( 3 \right) ,0.5)$ |
|  | $\tau_{\phi}$ | SD of mortality rate across all releases | $\tau_{\phi}\sim Gamma(1.5,6)$ |
|  | $\phi_{i}$ | Mortality rate for fish from release *i* | $\phi_{i}\sim LogNormal(\underline{log(\phi)},\tau_{\phi})$ |
| **Adult mortality** | | |  |
|  | $\kappa_{1}$ | Monthly log mortality rate for CS more than 24 months of age. This is equivalent to having a survival rate of 0.90 for fish age 4 and older. | $\kappa_{1}=loglog \left( -\frac{loglog \left( 0.90 \right)}{12} \right) = -4.735$ |
|  | $\kappa_{2}$ | Slope of monthly log mortality rate for CS. This value combined with the $\kappa_{1}$ means that the annual survivorship for the youngest fish in the model is 0.60. | $\kappa_{2}=0.087$ |
|  | $\eta_{m}$ | Monthly mortality rate for age *m* fish. | *Deterministic function of* $\kappa_{1},\kappa_{2}$ |
|  | $M_{a}$ | Mortality for season ending at age *a* | *Sum of* $\eta_{m}$ *within each season* |
| **Maturity and leaving the ocean** | | |  |
|  | $\gamma_{1,r}$ | Intercept for logistic regression for maturation proportion | $\gamma_{1,r}\sim$*Normal(-5,1^2^)* |
|  | $\gamma_{2,r}$ | Slope for logistic regression for maturation proportion | $\gamma_{2,r}\sim$*LogNormal(0,0.25)* |
|  | $\varsigma_{r}$ | Spatial smoothing parameter that determines the proportion of fish in a region migrating into a river to spawn | $\varsigma_{r}\sim$*Gamma(12,2)* |
|  | $f_{i}$ | Fraction of the time at which spawning fish leave the ocean during the fall season | *Fixed* $f_{i}=0.33$ |
|  | $\delta_{r,l,y}$ | Proportion of fish that leave the ocean in year *y* due to maturation. Derived from freshwater recoveries information | *Deterministic function of* $\gamma_{1,r}$*,* $\gamma_{2,r}$*,* $\varsigma_{r}$ |
|  | $D_{i,y}$ | Latent variable for age *y* fish leaving the ocean in the fall. | *N/A* |
|  | $p_{1,r,y}$ | Proportion of age *y* fish that are observed leaving the ocean from a given cohort. | *N/A* |
|  | $n$ | Effective sample size for the Dirichlet distribution informing the age composition of fish leaving the ocean in | *Fixed, 100* |
| **Fishing mortality parameters** | | |  |
|  | $\alpha$ | Vulnerability intercept (logit scale) | *Fixed,* $\alpha=4.6$ |
|  | $\beta_{1,g}$ | Vulnerability slope as a function of retention size for each gear type | $\beta_{l,g}\sim$*Gamma(3,3)* |
|  | $\beta_{1,g=Trawl}$ | Vulnerability slope coefficient for the linear term (hake trawl gear type) | $\beta_{1,g=Trawl}\sim Normal(-1,1)$ |
|  | $\beta_{2,g=Trawl}$ | Vulnerability slope coefficient for the quadratic term in (hake trawl gear type) | $\beta_{2,g=Trawl}\sim Normal(-1,1)$ |
|  | ${log(q}_{g,c})$ | Catchability of fall CS for a given gear type in a given calendar year. Gear types include troll, treaty troll, recreational (both US and Canadian), and A-SHOP and Shoreside hake trawl. | *Function of* $Q_{0,g},Q_{1,g}, and Q_{2}$ |
|  | $Q_{0,g}$ | Asymptotic value for log *q* | $Q_{0,troll}\sim$ *Normal(-10,1*^2^*)*  $Q_{0,rec-US}\sim$ *Normal(-13,1*^2^*)*  $Q_{0,rec-CAN}\sim$ *Normal(-13,1*^2^*)*  $Q_{0,treaty}\sim$*Normal(-8,1*^2^*)*  $Q_{0,hake}\sim$*Normal(-15,1*^2^*)* |
|  | $Q_{1,g}$ | Slope parameter determining the rate of change for log-q | $Q_{1,g}\sim$*Gamma(12,8)* |
|  | $Q_{2}$ | Time point at which log q reaches a point halfway between its minimum and maximum value. | $Q_{1,g}\sim$*Normal(-5,2*^2^*)* |
|  | $v_{a,l,g}$ | Vulnerability of age *a* fish to gear *g* in region *l.* | *Determined by*$\alpha$*,* $\beta_{l,g}$ |
|  | $\chi_{c,s,l,g}$ | Parameter for regions missing fishing effort | $\chi_{c,s,l,g}\sim LogNormal(\underline{loglog \left( \rho\right)},\varepsilon)$ |
|  | $log(\rho)$ | Parameter scaling the median fishing mortality for time intervals lacking fishing effort | $log\left( \rho\right)\sim$*Normal(log(0.01),1*^2^*)* |
|  | *h* | Parameter scaling unobserved mortality associated with fishing activities | *Fixed, h = 0.1* |
|  | $\varepsilon$ | Parameter describing the variability among fishing mortalities for time intervals lacking fishing effort | $\varepsilon$ *~Gamma(2,2)* |
|  | $F_{a,s,c,l,g}$ | Density-independent rate of fishing mortality for retained fish | *Determined by fishing effort E and lower level fishing morality parameters.* |
|  | $U_{a,s,c,l,g}$ | Density-independent rate of fishing mortality for non-retained fish | *Determined by fishing effort E, scaling parameter h, and lower level fishing morality parameters.* |
| Observation Parameters | | |  |
|  | $\mu_{1,i,a,l,g}$,  $\mu_{1,k,a,l,g}$ | Predicted catch | *Determined by many of the lower level parameters.* |
|  | $\sigma_{1,g}$ | CV of catch rate for each gear group | $\sigma_{1,g}\sim$*Gamma*(50,50) |
|  | $\mu_{2,c,r}$ | Predicted escapement | *Determined by many of the lower level parameters.* |
|  | $\sigma_{2,c,r}$ | Standard deviation of escapement | *Fixed,* $\sigma_{2,c,r}$ *= 0.1/0.2* |
|  | $\sigma_{3}$ | CV of total predicted catch | *Fixed* $\sigma_{3}$ *= 0.1* |
|  | $p_{2,l,s,c,r,g}$ | Predicted proportional contribution of stock *r* to catch | *Determined by many of the lower level parameters* |
|  | $p_{2,l,F}$ | Expected proportional contribution of focal stocks to catch in region *l* | $p_{l,F}\sim Normal(p_{obs,l,F},sqrt\left( 2 \right))$ |
|  | $\sigma_{4}$ | Standard deviation of focal stock contributions to catch | $\sigma_{4}\sim Gamma(10,100)$ |
|  | $p_{3,a,s,c,r}$ | Expected proportional contribution of age *a* fish abundance from stock *r* to total stock *r* abundances for ages years 2-5. | *Determined by many of the lower level parameters* |
|  | | |  |
| Indexes | | |  |
|  | *c* | Calendar year. Model includes years 1979-2015 | |
|  | *i* | CWT hatchery release group indicator | |
|  | *j* | Tag Code indicator | |
|  | *k* | Overall stock release group indicator | |
|  | *l* | Ocean region; 17 regions shown in Figure 1. | |
|  | *g* | Gear type. Gear types include recreational and troll (including both commercial and treaty troll) | |
|  | *s* | Season. Winter (November-February), Spring (March-May), Summer (June-July) and Fall (August-October). | |
|  | *y* | Spawner age. Corresponds to the age in years of fish leaving to spawn. Fish are indexed as aging up at the beginning of fall each year. | |
|  | *r* | Origin region. Typically corresponds to the ocean region that contains the mouth of the origin river. See Table S2.1 | |
|  | *m* | Month | |
|  | *a* | Model age in seasons. The model contains a total of 19 seasons | |
|  | *F* | Indicates the sum of all focal (modeled) stocks | |
|  | *NF* | Indicates the sum of all non-focal (not modeled) stocks | |

Table S1.3. Distance Matrices for specific origin regions. Columns are origins, rows are regions.

|  | SFB | CAC | KLT | NCASOR |
| --- | --- | --- | --- | --- |
| MONT | 1 | 3 | 3 | 3 |
| SFB | 0 | 2 | 2 | 2 |
| MEND | 1 | 1 | 1 | 1 |
| NCA | 2 | 0 | 0 | 0 |
| SOR | 3 | 1 | 1 | 0 |
| NOR | 4 | 2 | 2 | 1 |
| COL | 5 | 3 | 3 | 2 |
| WAC | 6 | 4 | 4 | 3 |

**References**

Allen, S. D., Satterthwaite, W. H., Hankin, D. G., Cole, D. J., & Mohr, M. S. 2017. Temporally varying natural mortality: Sensitivity of a virtual population analysis and an exploration of alternatives. *Fisheries Research*, 185, 185– 197.

Allen-Moran, S.D., Satterthwaite, W.H., and Mohr, M.S. 2013. Sample Size Recommendations for Estimating Stock Composition Using Genetic Stock Identification (GSI). NOAA Technical Memorandum NMFS, NOAA-TM-NMFS-SWFSC-513.

Anderson, C.W., and Ward, D. 2016. Results of Freshwater Creek salmonid life cycle monitoring station 2015-2016. Humboldt State University, Department of Fisheries Biology.

Baker, P.F. and Morhardt, E. 2001. Survival of Chinook salmon smolts in the Sacramento-San Joaquin Delta and Pacific Ocean. *In:* R.L. Brown, ed. Contributions to the biology of Central Valley salmonids. Fish Bull. 179: Volume 2. California Department of Fish and Game, Sacramento. pp. 163-182

Banerjee, S., Gelfand, A.E., Finley, A.O., and Sang, H. 2008. Gaussian predictive process models for large spatial data sets. Journal of the Royal Statistical Society B 70: 825–848.

Baranov, T.I. 1918. On the question of the biological basis of fisheries. Nauch. Issledov. Iktiol. Inst. Izv. I 1: 81–128.

Bellinger, M. R., M. A. Banks, S. J. Bates, E. D. Crandall, J. C. Garza, G. Sylvia, and P. W. Lawson. 2015. Geo-referenced, abundance calibrated ocean distribution of Chinook salmon (Oncorhynchus tshawytscha) stocks across the west coast of North America. PLoS ONE 10(7): e0131276.

Beverton, R.J.H., and Holt, S.J. 1957. On the dynamics of exploited fish populations. Chapman & Hall.

California Department of Fish and Wildlife. 2021. GrandTab 2021.06.30: California Central Valley Chinook Population Database Report. https://www.wildlife.ca.gov/Conservation/Fishes/Chinook-Salmon/Anadromous-Assessment

California Department of Fish and Wildlife. 2019. Klamath River Basin Spring Chinook Salmon Spawner Escapement, River Harvest, and Run-Size Estimates 1980-2018. <https://wildlife.ca.gov/Conservation/Fishes/Chinook-Salmon/Anadromous-Assessment>

California Department of Fish and Wildlife. 2022. Klamath River Basin Fall Chinook Salmon Spawner Escapement, In-river Harvest and Run-size Estimates, 1978-201. <https://wildlife.ca.gov/Conservation/Fishes/Chinook-Salmon/Anadromous-Assessment>

Clemento, A.J., Crandall, E.D., Garza, J.C., and Anderson, E.C. 2014. Evaluation of a single nucleotide polymorphism baseline for genetic stock identification of Chinook salmon (*Oncorhynchus tshawytscha*) in the California Current large marine ecosystem. Fishery Bulletin 112: 112-130.

Finley, A.O., Sang, H., Banerjee, S., and Gelfand, A.E. 2009. Improving the performance of predictive process modeling for large datasets. Computational Statistics and Data Analysis 53: 2873–2884. doi:10.1016/j.csda.2008.09.008.

Hankin, D.G. and Logan, E. 2010. A preliminary analysis of Chinook salmon coded-wire tag recovery data from Iron Gate, Trinity River and Cole Rivers Hatcheries, brood years 1978–2004. Prepared for The Hoopa Valley Tribal Council and the Arcata Office, U.S. Fish and Wildlife Service. Available at: <https://www.fws.gov/arcata/fisheries/reports/technical/IGHTRH.CWTanalysis2009%20Hankin%20Rpt.pdf>

Klamath River Technical Team. 15 February 2021. Klamath River Fall Chinook Salmon Age-Specific Escapement, River Harvest, and Run Size Estimates, 2020 Run

Jensen, A.J., Kelly, R.P., Anderson, E.C., Satterthwaite, W.H., Shelton, A.O., and Ward, E.J. 2022. zoid: A mixture model (and R package) for modeling proportional data with 0s and 1s in ecology. Ecology. 103(11): e3804.

Kilduff, D.P., Botsford, L.W., and Teo, S.L.H. 2014. Spatial and temporal covariability in early ocean survival of Chinook salmon (*Oncorhynchus tshawytscha*) along the west coast of North America. ICES J. Mar. Sci. 71(7): 1671–1682. doi:10.1093/icesjms/fsu031.

McHugh P, Johnson G, Schaffler, J. 2015. Chinook FRAM base period documentation: growth functions. Pacific Fisheries Management Council. Available at, <https://www.pcouncil.org/documents/2015/11/agenda-item-d-2-attachment-2-2.pdf/>

Mohr MS. 2006. Klamath River fall Chinook assessment: overview. Unpublished report. Santa Cruz (CA): National Marine Fisheries Service. Available from Michael S. Mohr, National Marine Fisheries Service, 110 McAllister Way, Santa Cruz, CA 95060, USA.

Mohr MS, O’Farrell MR. 2014. The Sacramento Harvest Model (SHM). NOAA Technical Memorandum NMFS-SWFSC-525. Available from: [https://swfsc.noaa.gov/publications/TM/SWFSC/NOAA- TM-NMFS-SWFSC-525.pdf](https://swfsc.noaa.gov/publications/TM/SWFSC/NOAA-%20TM-NMFS-SWFSC-525.pdf)

O’Farrell, M.R., and Satterthwaite, W.H. 2021. A rebuilding time model for Pacific salmon. Fisheries Research 238: 105900.

Oregon Department of Fish and Wildlife. 2013. Conservation plan for fall Chinook salmon in the Rogue Species Management Unit. Adopted by the Oregon Fish and Wildlife Commission, January 11, 2013.

PFMC (Pacific Fishery Management Council). 2019a. Review of 2018 Ocean Salmon Fisheries Stock Assessment and Fishery Evaluation Document for the Pacific Coast Salmon Fishery Management Plan. Pacific Fishery Management Council, 7700 NE Ambassador Place, Suite 101, Portland, OR 97220-1384, USA.

PFMC (Pacific Fishery Management Council). 2019b. Stock abundance analysis and evironmental assessment part 1 for 2019 ocean salmon fishery regulations. Pacific Fishery Management Council, 7700 NE Ambassador Place, Suite 101, Portland, OR 97220-1384, USA.

Pacific Fishery Management Council. 2020. Preseason Report I: Stock abundance analysis and environmental assessment part 1 for 2020 ocean salmon fishery regulations. Regulation Identifier Number 0648-BJ48. [www.pcouncil.org](http://www.pcouncil.org)

Pacific Fishery Management Council. 2021. *Preseason Report I: Stock Abundance Analysis and Environmental Assessment Part 1 for 2021 Ocean Salmon Fishery Regulations.* (Document prepared for the Council and its advisory entities.) Pacific Fishery Management Council, 7700 NE Ambassador Place, Suite 101, Portland, Oregon 97220-1384.

Seeb, L.W., Antonovich, A., Banks, M.A., Beacham, T.D., Bellinger, M.R., Blankenship, S.M., Campbell, M.R., Decovich, N.A., Garza, J.C., Guthrie III, C.M., Lundrigan, T.A., Moran, P., Narum, S.R., Stephenson, J.J., Supernault, K.J., Teel, D.J., Templin, W.D., Wenburg, J.K., Young, S.F., and Smith, C.T. 2007. Development of a standardized DNA database for Chinook salmon. Fisheries 32(11): 540-552.

Satterthwaite WH, Mohr MS, O’Farrell MR, Wells BK 2013. A comparison of temporal patterns in the ocean spatial distribution of California’s Central Valley Chinook salmon runs. Canadian Journal of Fisheries and Aquatic Sciences, 70, 574–584.

Satterthwaite, W.H., O’Farrell, M.R., and Mohr, M.S. 2013. Klamath-Trinity Basin Fall Run Chinook Salmon Scale Age Analysis Evaluation. NOAA Technical Memorandum NMFS, NOAA-TM-NMFS-SWFSC-522.

Satterthwaite, W. H., M. S. Mohr, M. R. O’Farrell, E. C. Anderson, M. A. Banks, S. J. Bates, M. R. Bellinger, L. A. Borgerson, E. D. Crandall, J. C. Garza, B. J. Kormos, P. W. Lawson, and M. L. Palmer-Zwahlen. 2014. Use of genetic stock identification data for comparison of the ocean spatial distribution, size-at-age, and fishery exposure of an untagged stock and its indicator: California Coastal versus Klamath River Chinook salmon. Trans. Am. Fish. Soc. 143:117-133.

Satterthwaite, W. H., J. Ciancio, E. Crandall, M. L. Palmer-Zwahlen, A. M. Grover, M. R. O’Farrell, E. C. Anderson, M. S. Mohr, and J. C. Garza. 2015. Stock composition and ocean spatial distribution inference from California recreational Chinook salmon fisheries using genetic stock identification. Fisheries Research 170: 166-178.

Satterthwaite, W.H., Carlson, S.M., and Criss, A. 2017. Ocean size and corresponding life history diversity among the four run timings of California Central Valley Chinook salmon. Transactions of the American Fisheries Society 146: 594-610.

Sharma, R., Vélez Espino, L.A. , Wertheimer, A.C., Mantua N., and Francis, R.C. 2013. Relating spatial and temporal scales of climate and ocean variability to survival of Pacific Northwest Chinook salmon (*Oncorhynchus tshawytscha*). Fisheries Oceanography 22: 14–31. doi:10.1111/fog.12001.

Shelton, A.O., J.T. Thorson, E.J. Ward, B.E. Feist. 2014. Spatial, semi-parametric models improve estimates of species abundance and distribution. Canadian Journal of Fisheries and Aquatic Sciences 71:1655-1666.

Shelton, A. O., Satterthwaite, W. H., Ward, E. J., Feist, B. E., & Burke, B. 2019. Using hierarchical models to estimate stock-specific and seasonal variation in ocean distribution, survivorship, and aggregate abundance of fall run Chinook salmon. Canadian Journal of Fisheries and Aquatic Sciences, 76(1), 95-108. doi:10.1139/cjfas-2017-0204.

Sturrock, A. M., W. H. Satterthwaite, K. M. Yoshida, E. R. Huber, H. J. W. Sturrock, S. Nusslé, and S. M. Carlson. 2019. Eight decades of hatchery salmon releases in the California Central Valley: Factors influencing straying and resilience. Fisheries 44:433-444.

United States Fish and Wildlife Service (USFWS). 1960. Natural Resources of Northwestern California. Report. Appendix: a preliminary survey of fish and wildlife resources. 104 p.

Zach Larson and Associates. 2013. Operation of dual frequency identification sonar (DIDSON) to monitor adult anadromous fish migrations in the Smith River, California: 2-year pilot study. Submitted to: California Department of Fish and Wildlife and the County of Del Norte.
