# Supplementary material for: Modeling ocean distributions and abundances of natural- and hatchery-origin Chinook salmon stocks with integrated genetic and tagging data
Source: PeerJ. 2023 Nov 28;11:e16487. doi: 10.7717/peerj.16487 (PMC10691356; doi:10.7717/peerj.16487)
Supplement: Supplemental Information 2 [file peerj-11-16487-s002.docx]

Jensen et al. Supplement S2

Overview

We note in section headers throughout whether described data are applied in all models, only models including GSI data (*CWT+GSI, CWT+GSI+Age*), or only models including both GSI and age data (*CWT+GSI+Age*)

Fisheries and Salmon Tagging Data (all models)

Fisheries Effort and Sampling Fractions

Fishing effort statistics for Chinook salmon (CS), utilized in model estimation of fishing mortality, are reported in a variety of ways by different governmental agencies. We use the same data sources and methods as those presented in Shelton et al. (2021), providing effort data on troll, recreational, and trawl fisheries. The major addition for our analyses is that we have updated all data sets through 2018. In a departure from Shelton et al. (2021), we truncated the spatial range of our analysis and so only include information from Washington, Oregon, and California (excluding the waters of the Strait of Juan de Fuca and Puget Sound in Washington).

Using publicly available documents, grey literature, and other publications, we identified when CS retention was allowed for each gear group and only retained effort information for periods in which CS retention was allowed.

**Troll Fisheries**

We collated commercial fisheries effort data for 1979-2018 for Washington, Oregon, and California (Fig. S2.1, S2.3). Troll fisheries effort was reported in boat-days for all fisheries except the treaty troll fishery that occurs in the waters of Washington state and reports effort in terms of deliveries. For all troll fisheries, we derive estimates of sampling fraction from the RMIS database (Figs. S2.2, S2.4).

**Recreational Fisheries**

Recreational effort data largely comes from creel surveys conducted by state or provincial governments. Recreational effort for coastal Washington, Oregon, and California is reported in documents produced by the Pacific Fisheries Management Council. This document is known as the “blue book” and effort is reported in angler-trips by major port and month (https://www.pcouncil.org/safe-documents-3/). We assign effort from ports to adjacent ocean regions. We extracted data at the statistical area level from publications and assigned effort to the appropriate ocean region (Fig. S2.5).

For all recreational fisheries, we derive estimates of sampling fraction from the RMIS database (Fig. S2.6).

**Pelagic Trawl Effort and Sampling (Hake fleets)**

In addition to the fisheries that target CS, we include data from two pelagic trawl fisheries that target Pacific hake (*Merluccius productus*) along the coasts of California, Oregon, and Washington. These fisheries are distinguished by whether they catch and deliver hake to land based plants for processing (the “shoreside” fleet) or whether they catch and process fish at-sea (the “at-sea” fleet). Importantly, RMIS does not provide accurate sampling fraction information for these bycatch fleets so we outline how we derive sampling fractions for each fleet (Fig. S2.8, S2.10).

*Shoreside fleet*

We used the Pacific Fisheries Information Network (PacFIN) Fish Ticket data set to quantify fishing effort for the shoreside fleet (PacFin retrieval dated 5/25/2019, Pacific States Marine Fisheries Commission, Portland, Oregon, (www.psmfc.org)). We identified hake fishing trips as trips that caught at least 50% hake and used one of the following gear types: Groundfish Trawl (GFT), Mid-water Trawl (MDT), Other Trawl (OTW), Roller Trawl (RLT). Because PacFIN data does not have comprehensive information on fishing trip length, we used multiple data sources and methods to estimate trip length. NOAA’s West Coast Groundfish Observer Program (WCGOP) provided trip length data derived from logbooks for MDT trips after 1990. We used the mean trip length by season and fishing region to estimate the length of MDT trips prior to 1990.

For non-MDT gear types, we estimated trip lengths using consecutive PacFIN departure dates for an individual vessel, this value is considered a maximum possible trip length. We filtered out trips that lasted longer than 10 days (known trip lengths were never longer than 10 days, average trip length was 2-3 days). Next, we mapped effort onto ocean regions. When latitudes and longitudes were not provided, we used management codes and descriptions provided in the “PACFIN_CATCH_AREA_DESCRIPTION” column. Occasionally, the management codes provided mapped to regions that spanned more than one of our ocean regions. In those circumstances, we assigned effort based on proportional overlap between regions. In some cases, the geo-referenced MDT logbook data informed ambiguous PacFIN fishing descriptions. Effort is reported as boat days in each season and ocean region per year (Fig. S2.7).

Prior to 2010, the shoreside hake fleet had only sporadic and irregular sampling of CS bycatch for CWT. While there are a few CWT recoveries for the shoreside fleet prior to 2011, we exclude this data because we cannot determine an appropriate sampling fraction for these observations. We treat the sampling fraction prior to 2010 as zero. Therefore, even though the shoreside fishery certainly caught CS before 2010, we have zero observations of CWT. Beginning in 2011, land-based catch monitors were required to sort 100% of a delivery. Monitors attempt to sample all salmon in a delivery for CWT’s. If that is not possible (i.e., there are too many fish), monitors subsampled 40 fish. However, nearly 100% of the CS caught have been surveyed for CWT’s as it is rare to have more than 40 per delivery.

*At-sea hake fleet*

Observed effort data for the at-sea hake fleet was provided by the At-Sea Hake Observer Program (A-SHOP) at NOAA’s Northwest Fisheries Science Center in Seattle (Fig. S2.9). This data set, pulled from the NorPac database, represents fishing from 1991 to present, and encompasses the period during which the fishery was completely domestic (Bailey et al., 1982; Hicks et al., 2014). Data from the catcher processor sector, which fishes and processes catch on board, and also the mothership sector, which receives catch from small catcher vessels and processes, are included. Earlier data from the joint venture and foreign fleets (1970s and 80s) were not included due to uncertainty about data quality. Prior to 1991 there was certainly CS bycatch and CWTs were recovered in the at-sea fleet, however, the methods and protocols were substantially different.

We used two-level data for fishing trips observed by A-SHOP observers: data related to the fraction of boats with an observer on board and data related to the fraction of total catch surveyed per tow, given there was an observer on board. We assigned fishing effort to ocean regions based on latitudes and longitudes associated with each haul. We multiplied the tow or catch-level (depending on year) annual expansion rates, provided by the A-SHOP Observer Program at NOAA NMFS, to calculate total effort. Expansion rates for the domestic fishery (1990 to 2015) are based on catch weight. To calculate the fraction of total effort surveyed for CWT by observers we needed two components: 1) the fraction of hauls observed and 2) the fraction of each catch (MT) that was surveyed. We used expansion factors, as mentioned above, to calculate the fraction of hauls observed. Next, we calculated the fraction of a haul that was surveyed for bycatch, given there was an observer onboard. To do this, we used the total weight of CS observed in a sample and the extrapolated weight to back calculate an expansion factor and fraction of catch sampled. Finally, we multiplied these two fractions (fraction of total fishing trips observed and fraction of catch (MT) observed) to get the fraction of total effort observed per season and region.

Coded wire tag release data

Coded wire tags have been for more than 50 years on the west coast of North America to understand the biology of salmon populations and manage fisheries (see Nandor et al., 2010; CTC, 2019). Briefly, young salmon - predominantly of hatchery origin - have a tiny wire with a unique code inserted into their snout and are then released into the wild. Batches of fish released at the same location by the same hatchery at the same time have identical CWT codes that are treated as a cohesive group for analysis. Tens of millions of CS are tagged each year along the west coast of North America. These fish are then recovered in ocean fisheries after months or years at sea or in rivers during a spawning migration and the tag can be recovered and identify

the origin and age of an individual fish.

We identified CWT tag codes of interest by drawing upon the hatchery groups used extensively in existing salmon management models (e.g., CTC, 2019) as well as our own exploration of the RMIS database (www.rpmc.org). We identified CS derived from brood years between 1977 and 2012 and released between 1978 and 2013 and arising from focal hatcheries from each of 4 stocks (Table S2.1). We also only included groups that had CWT and were adipose fin clipped; historically a clipped adipose fin was the indicator that a fish contained a CWT and should be sent to a laboratory so the tag could be extracted. In total we identified 3344 unique coded wire tag (CWT) group codes that met our criteria (Table S2.4).

We subsequently combined these CWT tag codes into single “release groups” arising from the same brood year, hatchery, stock origin, and release stage (e.g., fingerling or yearling). For every stock but CAC, we exclude release groups that had fewer than 5 CWT recoveries across all fisheries that were modeled (troll, recreational, trawl bycatch): we retained release groups with few CWT recoveries for CAC to include data from the maximum number of release groups for this already data-poor stock. This resulted in a total of 284 release groups. Table S2.1 provides an overview of the origin regions and the major rivers and hatchery groups associated with each region. Table S2.2 show release group identifiers corresponding to each release region. Table S2.3 summarizes the total number of CWT fish released across all years for each origin region in the model. Table S2.4 (in csv format) provides the tag codes queried before trimming for the number of recoveries.

Table S2.1. The four stocks included in the population dynamics model.

| Stock | River Basin(s) | Ocean Region of River Mouth | Notes |
| --- | --- | --- | --- |
| SFB | California Central Valley Rivers | SFB | Releases from the Sacramento and San Joaquin River basins. These are predominantly releases from Coleman, Feather, Merced, Mokelumne, Nimbus, and San Joaquin hatcheries. |
| CAC | Coastal California Rivers | SFB, MEN, NCA | Releases from California Coastal Rivers in California, including the Eel River, Mad River, and Redwood Creek. |
| KLT | Klamath-Trinity Rivers | NCA | Releases from the Klamath River basin predominantly from Irongate and Trinity hatcheries. |
| NCASOR | Northern California, Southern Oregon coastal rivers | NCA, SOR | Releases from the Smith River in northern California and Chetco River in southern Oregon |

Table S2.2. Hatcheries included in this analysis. These releases only include fall releases extracted from the RMIS database (predominantly run = 3 but also run = 8 and a few run =7). Summer and spring run fish are not included. The suffix ‘_small’ or ‘_large’ on some of the stock refers to hatchery that release fish at two sizes (or, equivalently, at two times of year). ‘large’ refers to yearling releases and ‘small’ to fingerling releases in many but not all cases.

| Stock | Hatchery Code | First Brood Year | Final Brood Year | Number of Brood Years |
| --- | --- | --- | --- | --- |
| SFB | Coleman | 1980 | 2012 | 33 |
| SFB | Feather | 1977 | 2012 | 36 |
| SFB | Merced | 1987 | 2012 | 20 |
| SFB | Mokelumne | 1980 | 2012 | 24 |
| SFB | Nimbus | 1982 | 2012 | 17 |
| SFB | San_Joaquin | 1977 | 1999 | 12 |
| SFB | Tehama | 1977 | 1984 | 5 |
| SFB | Yuba | 1983 | 2006 | 3 |
| CAC | LITTLE RIVER POND | 1985 | 1991 | 4 |
| CAC | Mad | 1980 | 1982 | 2 |
| CAC | Mad_large | 1989 | 2001 | 4 |
| CAC | PRAIRIE CR HATCHERY | 1992 | 1994 | 2 |
| CAC | REDWOOD CREEK PONDS | 1983 | 1987 | 4 |
| CAC | SILVERADO PLANT.BASE | 1983 | 1985 | 3 |
| CAC | WARM SPRINGS HATCHERY | 1984 | 2002 | 9 |
| KLT | Irongate | 1978 | 2012 | 33 |
| KLT | Trinity | 1977 | 2012 | 39 |
| NCASOR | Smith | 1978 | 2012 | 27 |
| NCASOR | Chetco | 1978 | 2012 | 7 |
| TOTAL | NA | NA | NA | 284 |

Table S2.3. Summary of coded wire tagged releases by stock across all years. The total number of coded wire tag releases included 163 million fish.

| Stock | Total Release Groups | Number of Years | Average CWT Released | Total CWT Released |
| --- | --- | --- | --- | --- |
| SFB | 150 | 36 | 853,829 | 128,074,311 |
| CAC | 28 | 17 | 34,577 | 968,147 |
| KLT | 72 | 36 | 462,830 | 33,323,766 |
| NCASOR | 34 | 30 | 60,723 | 2,064,583 |


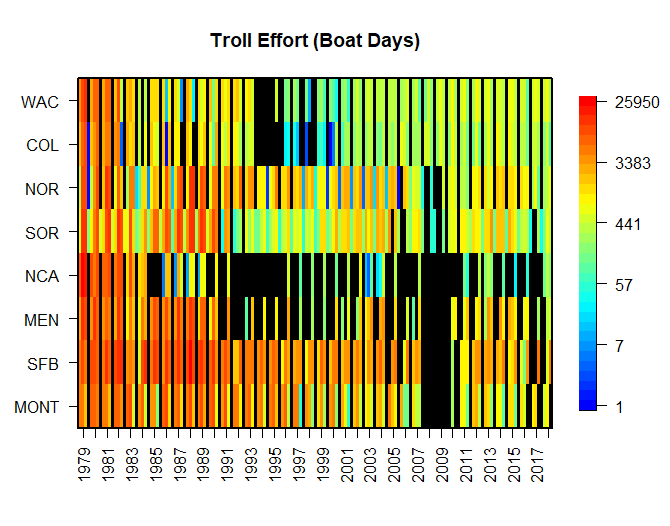


Figure S2.1. Commercial troll fishing effort in boat-days. The x-axis shows seasons; for each year the tick mark indicates the summer season. The y-axis corresponds to regions ordered south to north. Colors represent observed effort in each region-season (note the logarithmic color-scale). Black indicates no reported troll effort. In a small number of cases there are reported catches in locations and seasons without reported effort (see Supplement S1).


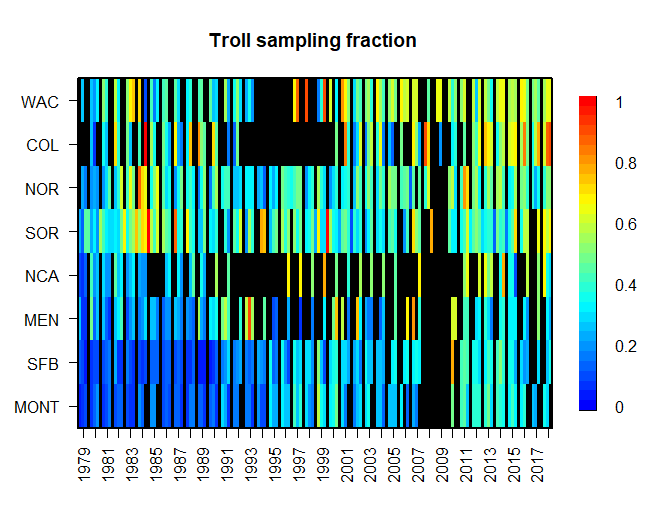


Figure S2.2. Commercial troll sampling fraction. The x-axis shows seasons; for each year the tick mark indicates the summer season. The y-axis corresponds to regions ordered south to north. These represent the proportion of the catch in each region-season that was sampled for coded wire tags. The color bar indicates the sampling fraction and black indicates no data is available for the sampling fraction.


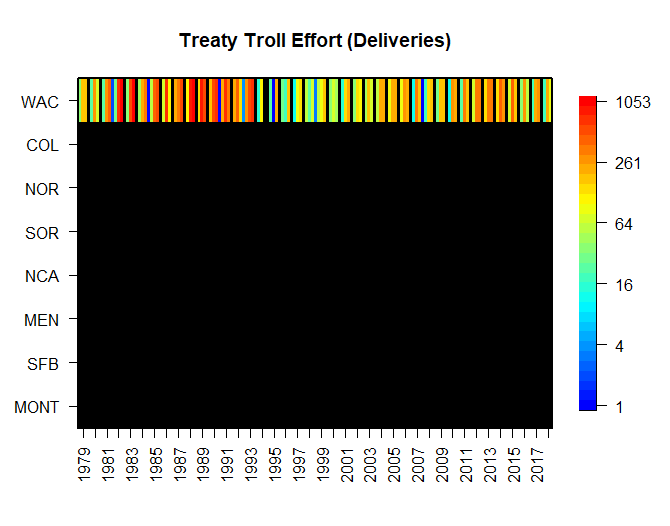


Figure S2.3: Treaty troll fishing effort in deliveries. For figure description see Figure S2.1.


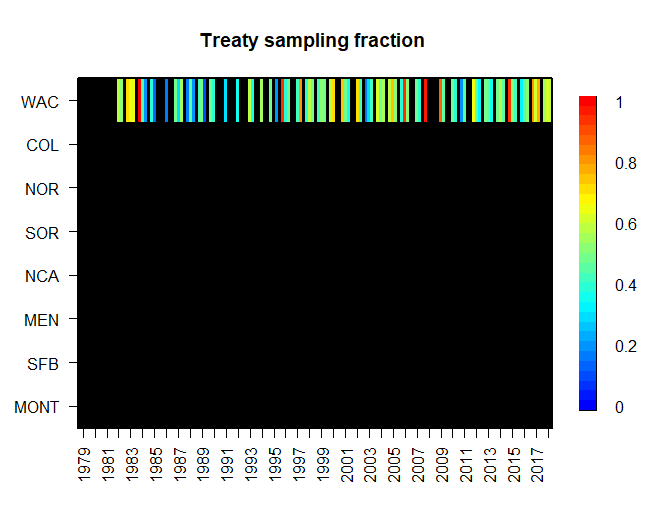


Figure S2.4: Treaty troll sampling fraction. For figure description see Figure S2.2.


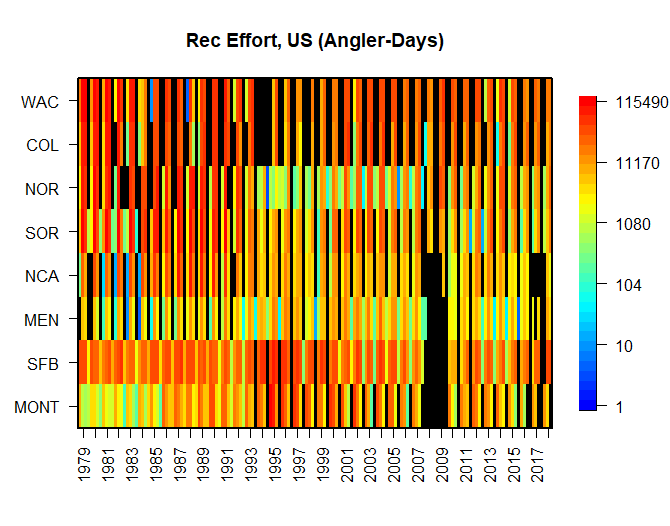


Figure S2.5. United States recreational fishing effort in angler-days. For figure description see Figure S2.1


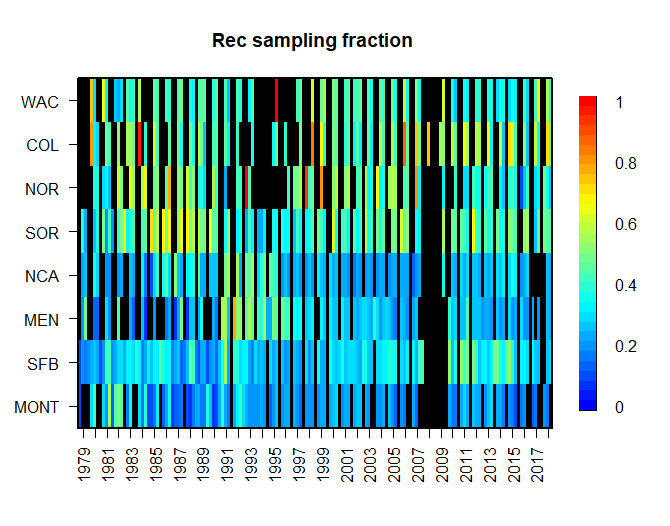


Figure S2.6. Recreational fishing sampling fraction. For figure description see Figure S2.2.


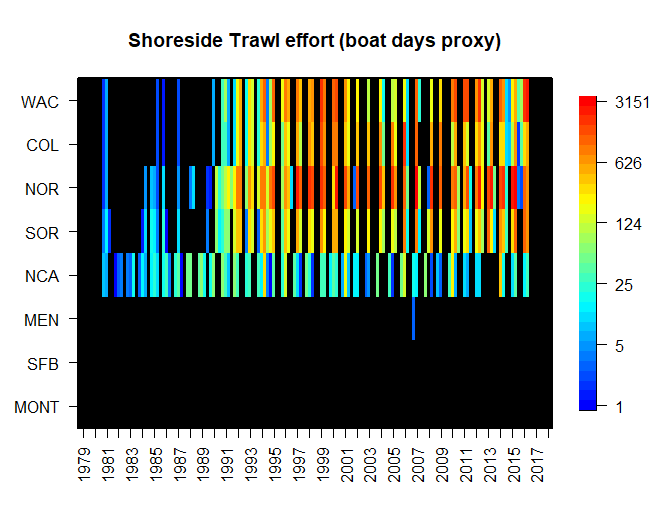


Figure S2.7. Shoreside hake fishing effort in boat days. For figure description see Figure S2.1.


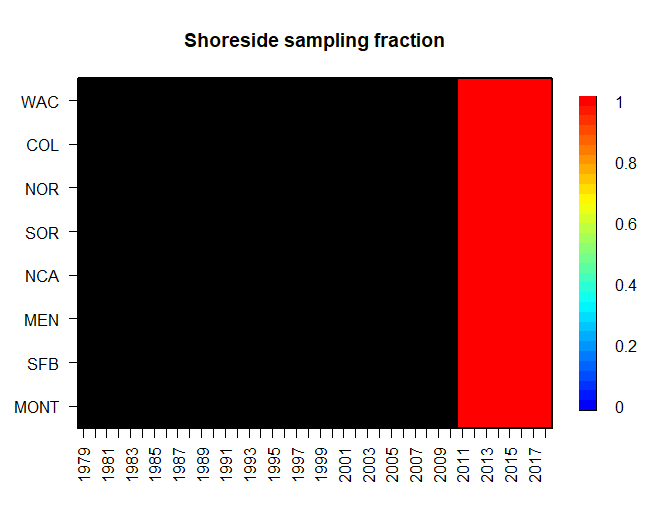


Figure S2.8. Shoreside hake sampling fraction. For figure description see Figure S2.2


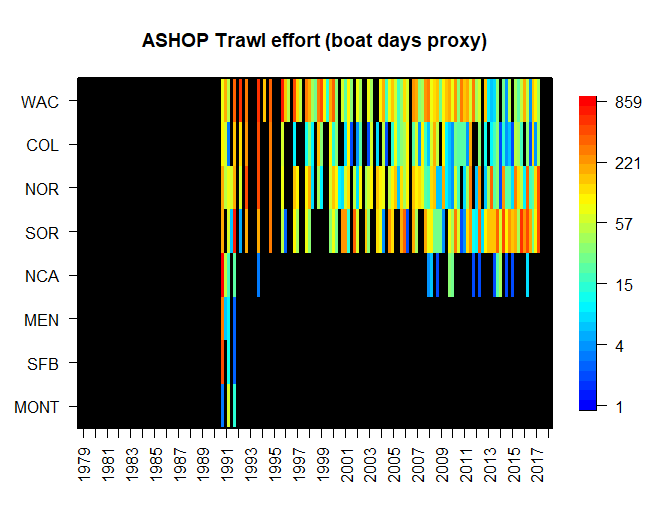


Figure S2.9. At-sea hake fishing effort in boat days. For figure description see Figure S2.1.


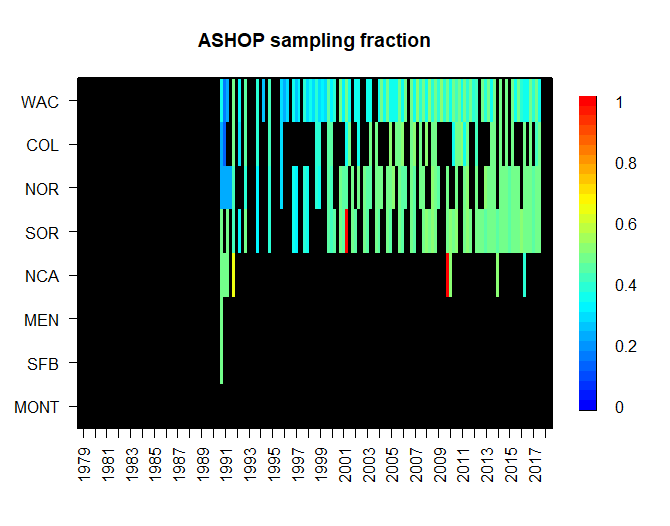


Figure S2.10. At-sea hake sampling fraction. For figure description see Figure S2.2.

CWT recovery data

Table S2.5. Summary of CWT recoveries for each stock by region, from 1979-2018. *North* represents all ocean regions north of WAC (see Shelton et al., 2021). In total, out-of-area CWT recoveries (North) for SFB, CAC, KLT, and NCASOR stocks represented just 0.9%, 6.4%, 0.3%, and 1.3% of all recoveries, respectively.

| Stock | CWT Recoveries by Region | | | | | | | | | |
| --- | --- | --- | --- | --- | --- | --- | --- | --- | --- | --- |
|  | MONT | SFB | MEN | NCA | SOR | NOR | COL | WAC | North | **Total** |
| SFB | 44295 | 105422 | 28193 | 11003 | 26572 | 24241 | 3033 | 6599 | 2201 | 251560 |
| CAC | 135 | 306 | 486 | 117 | 222.7 | 96.7 | 1.7 | 4.5 | 8.5 | 1378 |
| KLT | 552 | 3383 | 5678 | 5741 | 13546 | 2458 | 222 | 222 | 96 | 31898 |
| NCASOR | 80 | 271 | 637 | 378 | 2281 | 677 | 23 | 83.5 | 56.5 | 4487 |

Fisheries and Salmon Genetic Sampling Data (only models including GSI data)

**Recreational fisheries data from port-based sampling in California**

We first obtained GSI data from past sampling of recreational hook-and-line Chinook salmon landings in California (CA) ports by the California Department of Fish and Wildlife (CDFW). Tissue samples from approximately 20% of recreationally landed fish from 1998-2002 were collected (approximately 23,000 fin clips). Approximately half of these samples were genotyped; selection of samples was conducted using stratified random sampling by temporally- and spatially-defined strata. Fish were genotyped at 96 single-nucleotide polymorphism (SNP) loci, corresponding to loci used in the representative genetic baseline (Clemento et al. 2014). Genotyped fish were filtered to remove duplicate genotypes (i.e., indicative of mislabeled, duplicate, or contaminated tissue samples) and individuals with extreme individual heterozygosity. Genotypes then were compared against the SNP-based baseline using the software *gsi_sim* to obtain mixture proportions within temporally- and spatially-defined strata (i.e., 3-week sliding windows and management areas) and subsequently produce individual assignment (IA) probabilities to reporting groups for each fish (Anderson et al. 2008). Further sampling and genotyping details can be found in Satterthwaite et al. (2015).

We used all available individual assignment probabilities, as provided in the data file ‘CArec1998-2002PosteriorProbsEachIndividual.csv’, to summarize and analyze mixture proportions for the integrated state-space model. We removed all individuals with problematic IA probabilities (i.e., assigned to coho salmon, or probabilities do not conform to proportional space). We mapped port-specific sampling locations for genotypes to ocean region, mapped sampling dates to season, and calculated numbers of assigned fish to each reporting group or stock by summing group-specific probabilities across all fish within strata defined by year, season, and region. All formatting and filtering of CA recreational GSI data was conducted using the R script ‘Make CArec GSI.r’.

**Commercial troll data from the West Coast Salmon GSI Collaboration**

We obtained commercial troll data collected by Project CROOS (Collaborative Research on Oregon Ocean Salmon), as part of the West Coast Salmon GSI Collaboration. As part of this collaboration, commercial salmon troll fishermen in CA and Oregon (OR) collected tissue samples from retained fisheries harvest. Sampling intensity varied over space and time as a function of harvest constraints and the extent of participation by local fishermen; in 2010, for instance, approximately 24.1% of the OR and CA commercial fleet participated in sampling (Bellinger et al. 2015). Compared to recreational fisheries, we expect effort and harvest from commercial troll fisheries to be more broadly distributed away from large population centers; additionally, commercial fisheries generally have higher minimum size restrictions and fewer restrictions on daily harvest. Fish sampled from coastal waters of OR were genotyped at 13 microsatellite loci that corresponded to a standardized international baseline (Seeb et al., 2007). Fish sampled from CA were genotypes using a panel of 96 SNP loci used in a different baseline (Clemento et al., 2014). Genotypes were filtered out based on the number of genotyped loci, observed individual heterozygosity, and the degree of similarity among individuals. Genotypes were used to generate mixture proportions and IA probabilities within specified strata (i.e., 1-week sliding windows and management zones) using gsi_sim. More details on sampling programs and genotyping can be found in Satterthwaite et al. (2014) and Bellinger et al. (2015).

Data on IA probabilities and fish-specific metadata were obtained from CROOS project repositories. We used all available individual assignment probabilities, as provided in the data file ‘GSI Individuals_2019-08-20.csv’, to summarize and analyze mixture proportions for the integrated state-space model. We removed all individuals with problematic IA probabilities (i.e., if fish were assigned to coho salmon or their sums of group-specific probabilities either fell below 0.95 or exceeded 1.001; N=59), and standardized IA probabilities of remaining fish so that the sum for each fish was exactly 1 (sums sometimes deviated due to rounding errors). We mapped recorded latitude values of individual fish to ocean region using ‘Ocean spatial regions_CA_OR_WA_TWO_OR.csv’ as our reference, mapped sampling date to season, and calculated numbers of assigned fish to each reporting group or stock by summing group-specific probabilities across all fish within strata defined by year, season, and region. All formatting and filtering of GSI data was conducted using the R script ‘Make Croos GSI.r’.

**Commercial troll data from the West Coast Salmon GSI Collaboration**

We obtained data from voluntary tissue sampling conducted by commercial salmon fishermen in the coastal waters of Washington (WA). Fishermen representing approximately 35% of the total commercial fleet voluntarily and opportunistically collected tissue samples from retained Chinook salmon; financial incentives were offered to maintain sampling during periods of high catch. A random sample of tissue samples was genotyped at 13 microsatellite loci corresponding to the Seeb et al. (2007) baseline. Genotypes were removed from further analyses on the basis of missing loci, excessive homozygosity, and identical genotypes, and remaining genotypes were used to generate mixture proportions and IA probabilities for the total group of genotypes using ONCOR (Kalinowski et al., 2007). More details on the sampling program and genotyping can be found in Moran et al. (2018).

We used all available individual assignment probabilities, as provided in the data file ‘WA-troll_2012-2015.csv’, to summarize and analyze mixture proportions for the integrated state-space model (data available at [https://catalog.data.gov/dataset/chinook-salmon-genetic- stock-identification-data-genetic-stock-identification-of-washington-chi1](https://catalog.data.gov/dataset/chinook-salmon-genetic-stock-identification-data-genetic-stock-identification-of-washington-chi1) ). We removed all individuals with problematic IA probabilities and standardized IA probabilities of remaining fish so that the sum for each fish was exactly 1 (sums sometimes deviated due to rounding errors). We mapped recorded fishing area designations of individual fish to either the COL or WAC ocean region, mapped sampling date to season, and calculated numbers of assigned fish to each reporting group or stock by summing group-specific probabilities across all fish within strata defined by year, season, and region. All formatting and filtering of GSI data was conducted using the R script ‘Make WAcitsci GSI.r’.

**Aggregating and formatting total GSI data**

Across all GSI datasets, we only retained GSI data for a given year, season, and region if the number of sampled fish exceeded 10 due to concerns with model performance at lower sample sizes. We included GSI data from 1998-2014; we excluded GSI data after 2014 they potentially included fish from release broodyears 2013 and later, which were not modeled in this most recent iteration of the state-space model. We also restricted GSI observations to those that corresponded with positive estimates of landings (see *Landings Data*, below), as we were concerned these GSI observations potentially included periods of non-retention sampling and violated model assumptions of fishery processes. After these modifications, we were left with GSI data from every region, years 1998-2002, 2006-2007, and 2009-2014, and seasons spring, summer, and fall (Figs. S2.11, S2.12). Lack of consistent fishing effort in winter resulted in a lack of winter-based GSI data. All data formatting was conducted in R script ‘02b_Prep GSI data.R’.


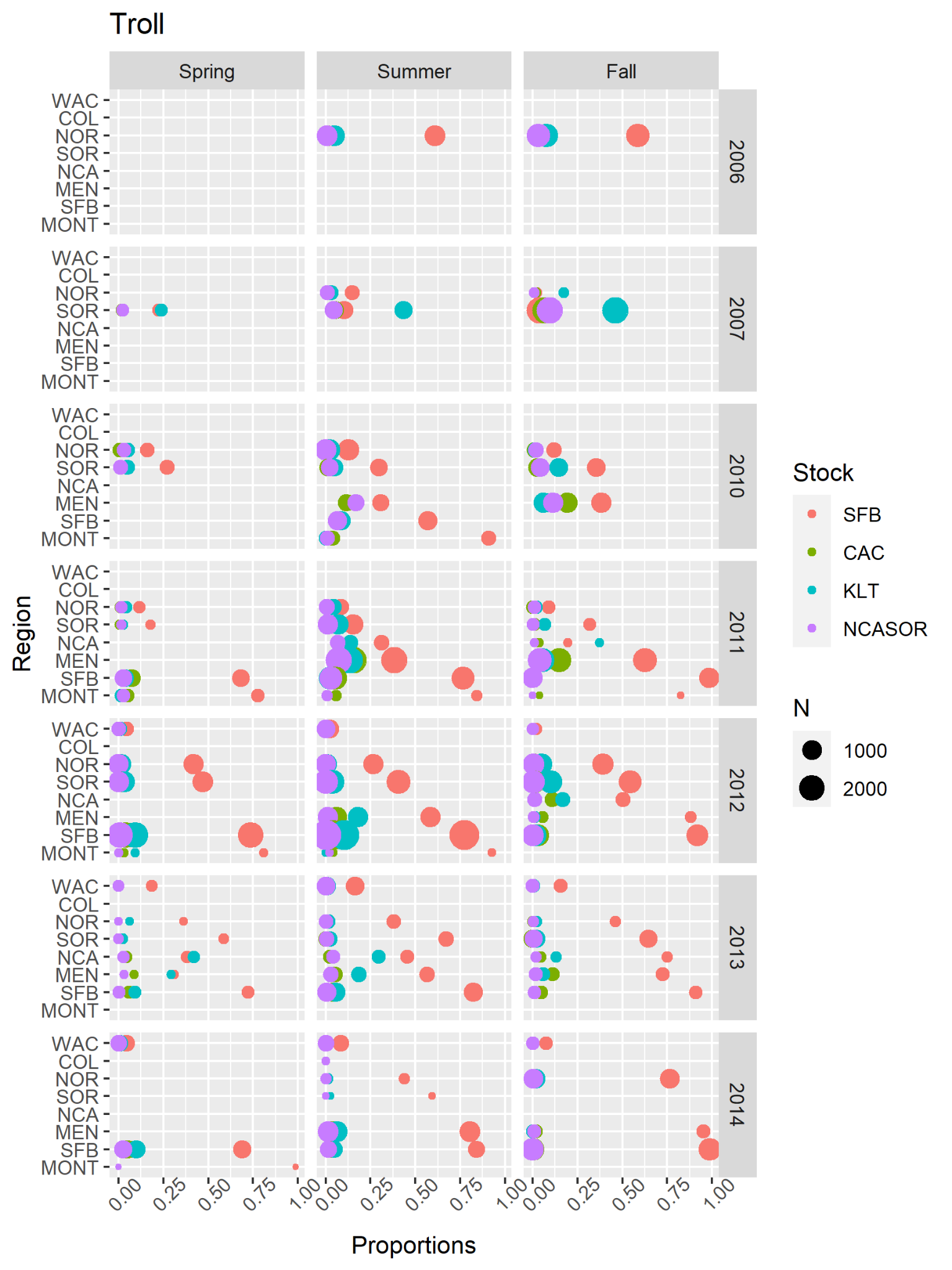


Fig. S2.11. Summary of GSI data from commercial troll fisheries. Points represented observed mixture proportions by stock for each combination of spatial region, season, and year. Point size (N) indicates the total number of sampled fish within each stratum.


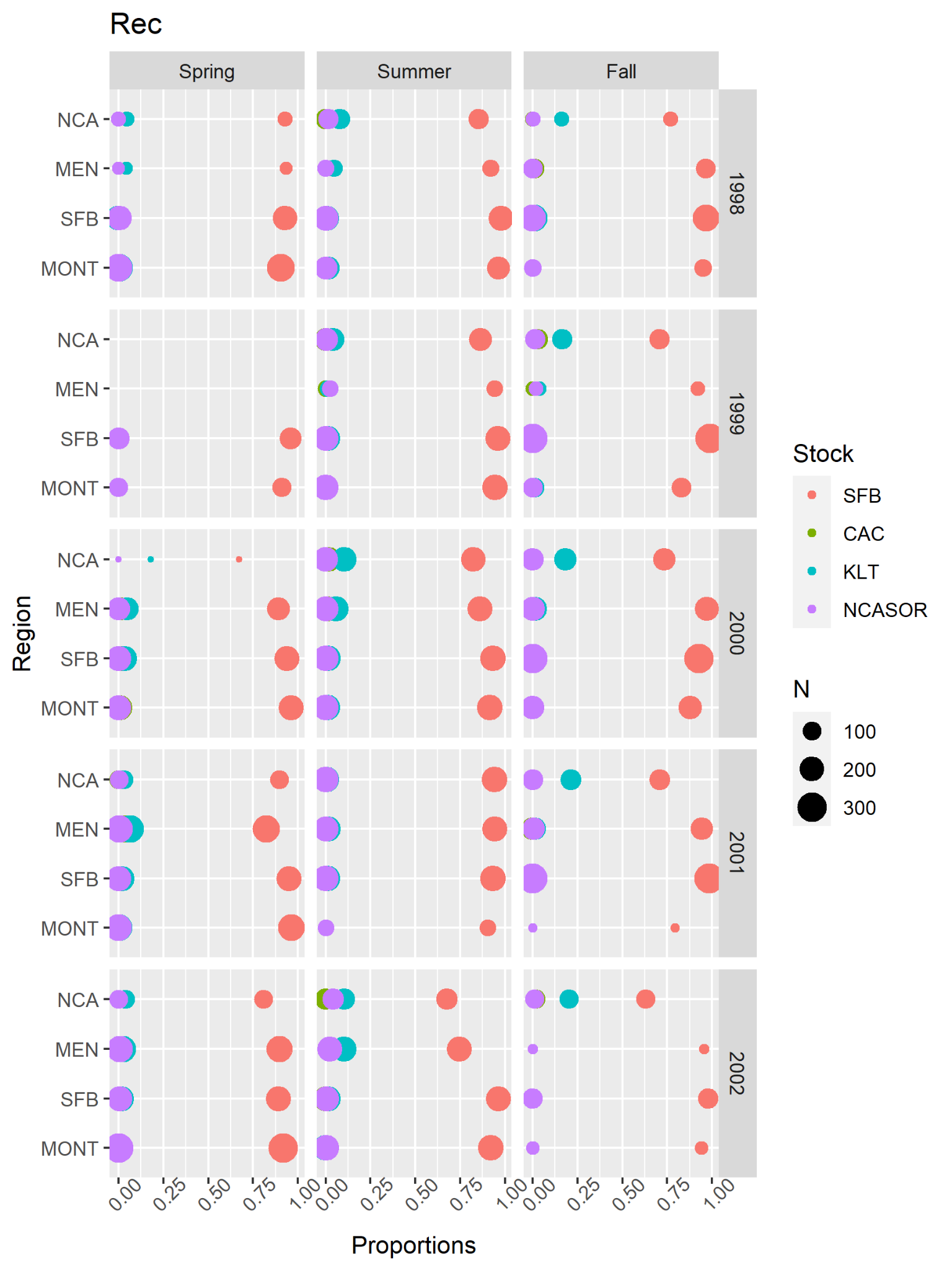


Fig. S2.12. Summary of GSI data from California recreational fisheries. Points represented observed mixture proportions by stock for each combination of spatial region, season, and year. Point size (N) indicates the total number of sampled fish within each stratum.

Run Size Data (only models including GSI data)

All aggregation and formatting of run size data (described below) was conducted in 'Make run size.r'. Data was read in from 'Compiled Run Size Data_8.10.21', which contained imported data from a variety of data sources for all relevant stocks.

**Central Valley fall-run (SFB)**

Escapement and river harvest estimates for SFB come from CDFW (2021) and PFMC (2020), respectively; we summed escapement estimates for fall-run, late fall-run, and Feather River Hatchery spring-run fish and fall-run harvest estimates on the Sacramento River to obtain a measure of total run size. Fall-run, late fall-run, and Feather River Hatchery spring-run fish are genetically highly similar in the Central Valley fall-run reporting group (Seeb et al., 2007; Clemento et al., 2014). Escapement estimates were generated from a combination of fish counts at hatcheries and dams, carcass surveys, live fish counts, and ground and aerial redd counts. Harvest estimates were predominantly estimated using data from annual angler surveys; we note our estimates of run size may exclude freshwater harvest in Central Valley watersheds other than the Sacramento River (e.g., the San Joaquin River). However, we expect the effect of this exclusion to be minimal. We considered all run size estimates from 1998-2014 to be reasonably complete and reliable (Fig. S2.13, Table S2.6).

**California Coast (CAC)**

Obtaining total run size estimates for CAC proved challenging because the stock includes numerous small and relatively isolated coastal watersheds with inconsistent historical survey and estimation efforts. For CAC, we used expanded estimates of redd counts from small coastal rivers in the Mendocino Coast survey region (CDFW unpublished data), video counts of migrating adults at Mirabel Dam on the Russian River (PFMC, 2020), and adult counts from weir sampling on Freshwater Creek (Anderson and Ward, 2016), in addition to baseline estimates of run size for Redwood Creek, Mad River, Eel River, and Mattole River based on preliminary tagging efforts (5,000, 5,000, 25,000, and 5,000 fish, respectively; USFWS, 1960). We note that CDFG (1965) warns ‘estimates given here which are based on little or no data should be used only in outlining the major and critical factors of the resource’, and that other historical estimates of expected abundance exist for some instances and suggest differing run sizes in select years (CDFG, 1965; Moyle, 2002). However, we feel the selected run size values reflect a reasonable baseline expectation, albeit with a large amount of uncertainty. We summed run size estimates, based on annual sampling or baseline values, across all described watersheds and regions to produce total annual run sizes. We consider all run size estimates from 1998-2014 to be incomplete due to the described data paucity in numerous river systems and years.

**Klamath River (KLT)**

Total run size estimates for spring- and fall-run Klamath River Basin Chinook salmon come from CDFW (2019, 2022). Run size estimates were generated as the sum of harvest estimates, based on creel surveys, and escapement estimates, based on video-assisted counts and a variety of field survey methods. We summed spring- and fall-run run size estimates to obtain total KLT run size again due to the inability to separate run timing groups using GSI alone. We consider all run size estimates to be reasonably reliable.

**North California/South Oregon Coast (NCASOR)**

For NCASOR, estimates of escapement for northern California’s Smith River (i.e., the only watershed with significant California Chinook salmon returns in the Southern Oregon / Northern California ESU) in return years 2010 and 2011 (i.e., 22,500 and 20,000, respectively) were obtained using DIDSON river monitoring and supplemental surveys (Zach Larson and Associates 2013); in all other years without escapement estimates, we assumed a run size of 15,000 from baseline estimates (USFWS, 1960). Estimates of spawning escapement for southern Oregon’s Chetco River, Winchuck River, Pistol River, Hunter Creek, and Euchre Creek, based on spawning surveys, were obtained from ODFW (2013) for 1998-2011. In years without escapement estimates, we assumed escapement was equal to mean escapement across all years with available estimates. Separate estimates of harvest for these systems were unavailable and assumed to be negligible. We summed run size estimates, based on annual sampling or baseline values, across all described watersheds to produce total annual run sizes. We consider all run size estimates from 1998-2014, with the exception of 2010 and 2011 which included empirical escapement estimates from all described watersheds, to be incomplete due to the described data paucity in numerous river systems and years.

Table S2.6. Summary of specified lognormal standard deviation for each stock- and year-specific estimate of run size. Standard deviations 0.1 and 0.2 correspond to estimates with relatively high and low reliability, respectively, based on the availability of annual data from contributing watersheds.

| Year | Specified standard deviation | | | |
| --- | --- | --- | --- | --- |
|  | *SFB* | *CAC* | *KLT* | *NCASOR* |
| 1998 | 0.1 | 0.2 | 0.1 | 0.2 |
| 1999 | 0.1 | 0.2 | 0.1 | 0.2 |
| 2000 | 0.1 | 0.2 | 0.1 | 0.2 |
| 2001 | 0.1 | 0.2 | 0.1 | 0.2 |
| 2002 | 0.1 | 0.2 | 0.1 | 0.2 |
| 2003 | 0.1 | 0.2 | 0.1 | 0.2 |
| 2004 | 0.1 | 0.2 | 0.1 | 0.2 |
| 2005 | 0.1 | 0.2 | 0.1 | 0.2 |
| 2006 | 0.1 | 0.2 | 0.1 | 0.2 |
| 2007 | 0.1 | 0.2 | 0.1 | 0.2 |
| 2008 | 0.1 | 0.2 | 0.1 | 0.2 |
| 2009 | 0.1 | 0.2 | 0.1 | 0.2 |
| 2010 | 0.1 | 0.2 | 0.1 | 0.1 |
| 2011 | 0.1 | 0.2 | 0.1 | 0.1 |
| 2012 | 0.1 | 0.2 | 0.1 | 0.2 |
| 2013 | 0.1 | 0.2 | 0.1 | 0.2 |
| 2014 | 0.1 | 0.2 | 0.1 | 0.2 |


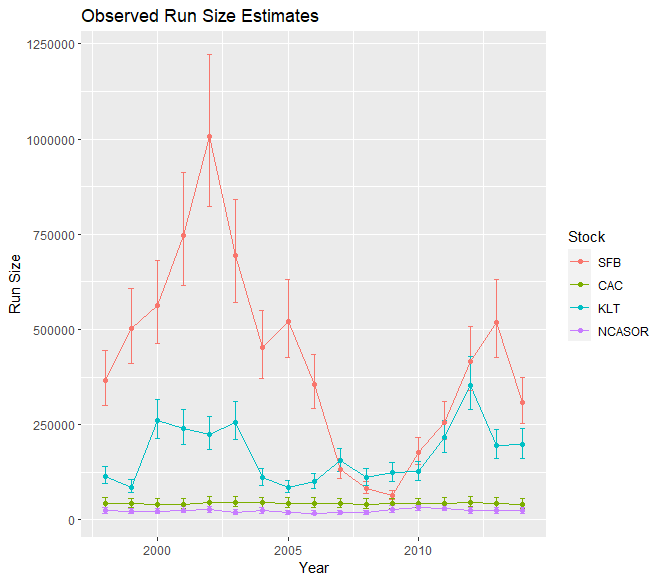


Figure S2.13. Estimated run sizes used as model constraints. Run size represents the sum of in-river harvest and escapement estimates, where available. Lines represent 95% confidence intervals that were calculated using the standard deviation values provided in Table S2.6.

Landings Data (only models including GSI data)

We obtained landings estimates for commercial troll and recreational hook-and-line fisheries, summarized primarily by port, month, and year, from the PFMC (2019). We mapped all port-based estimates to our ocean regions based on the spatial overlap of ports and regions except for Westport: for this port, we split landings between the COL and WAC regions using professional judgment. We further mapped monthly landings estimates to our four-season structure. Finally, we aggregated landings from each gear type by season, year, and ocean region to obtain observed total landings, $Z_{l,s,c,g}$. Patterns of observed landings are provided in Fig. S2.14.

All data formatting was conducted in the R script ‘Make PFMC landings GSI.R’, which imported landings data from the following aggregated data files: ‘CA_troll_landings.xlsx’, ‘OR_troll_landings.xlsx’, ‘WA_troll_landings.xlsx’, ‘CA_rec_landings.xlsx’, ‘OR_rec_landings.xlsx’, and ‘WA_rec_landings.xlsx’.


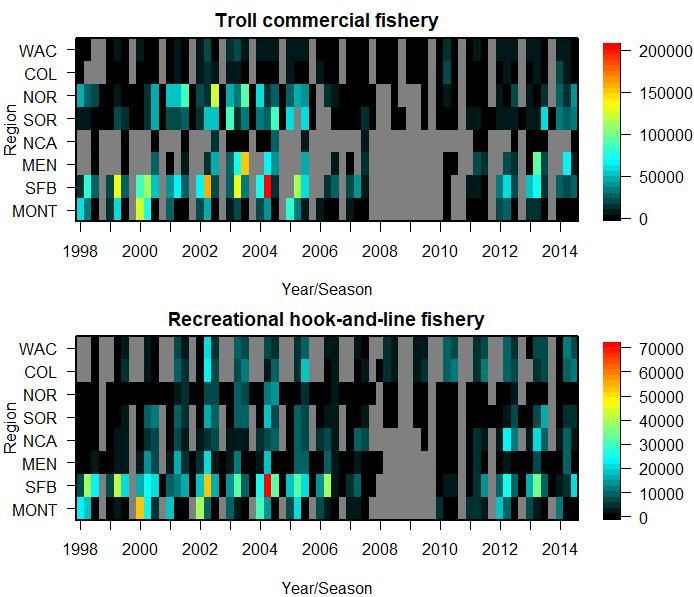
Figure S2.14. Summary of PFMC landings by gear type. Gray areas indicate region/season combinations with no apparent effort.

**References**

Anderson, E.C., Waples, R.S., and Kalinowski, S.T. 2008. An improved method for predicting the accuracy of genetic stock identification. Canadian Journal of Fisheries and Aquatic Sciences 65: 1475-1486.

Anderson, C.W., and Ward, D. 2016. Results of Freshwater Creek salmonid life cycle monitoring station 2015-2016. Humboldt State University, Department of Fisheries Biology.

Bailey KM, Francis RC, Stevens PR (1982) The life history and fishery of Pacific whiting, *Merluccius productus*. Calif. Coop. Oceanic Fish. Invet. Rep., 23, 81–98.

Bellinger, M. R., M. A. Banks, S. J. Bates, E. D. Crandall, J. C. Garza, G. Sylvia, and P. W. Lawson. 2015. Geo-referenced, abundance calibrated ocean distribution of Chinook salmon (Oncorhynchus tshawytscha) stocks across the west coast of North America. PLoS ONE 10(7): e0131276.

California Department of Fish and Game (CDFG). 1965. California Fish and Wildlife Plan, Volume 3, Supporting Data. Part B, Inventory (Salmon-Steelhead and Marine Resources). October 1, 1965

California Department of Fish and Wildlife. 2021. GrandTab 2021.06.30: California Central Valley Chinook Population Database Report. [https://www.wildlife.ca.gov/Conservation/Fishes/Chinook-Salmon/Anadromous- Assessment](https://www.wildlife.ca.gov/Conservation/Fishes/Chinook-Salmon/Anadromous-%09Assessment)

California Department of Fish and Wildlife. 2019. Klamath River Basin Spring Chinook Salmon Spawner Escapement, River Harvest, and Run-Size Estimates 1980-2018. <https://wildlife.ca.gov/Conservation/Fishes/Chinook-Salmon/Anadromous-Assessment>

California Department of Fish and Wildlife. 2022. Klamath River Basin Fall Chinook Salmon Spawner Escapement, In-river Harvest and Run-size Estimates, 1978-201. <https://wildlife.ca.gov/Conservation/Fishes/Chinook-Salmon/Anadromous-Assessment>

Clemento, A.J., Crandall, E.D., Garza, J.C., and Anderson, E.C. 2014. Evaluation of a single nucleotide polymorphism baseline for genetic stock identification of Chinook salmon (*Oncorhynchus tshawytscha*) in the California Current large marine ecosystem. Fishery Bulletin 112: 112-130.

CTC (Chinook Technical Committee) (2019) Annual report of catch and escapement for 2018. Pacific Salmon Commission Joint Chinook Technical Committee Report TCCHINOOK(19)–1, Vancouver, BC, pp.

Hicks AC, Taylor N, Grandin C, Taylor IG, Cox S (2014) Status of the Pacific hake (whiting) stock in US and Canadian waters in 2013. International Joint Technical Committee for Pacific Hake, Seattle, Washington, USA.

Kalinowski, S.T., Manlove, K.R., and Taper, M.L. 2007. ONCOR: a computer program for genetic stock identification. Montana State University, Department of Ecology, Bozeman.

Moran, P., J. Dazey, L. LaVoy, and S. Young. 2018. Genetic mixture analysis supports recalibration of the fishery regulation assessment model. Fisheries, 43: 83-97. doi:10.1002/fsh.10017

Moyle, P.B. 2002. Inland Fishes of California. University of California Press, Berkeley, California.

Nandor GF, Longwill JR, Webb DL (2010) Overview of the coded wire tag program in the Greater Pacific Region of North America. In: PNAMP Special Publication: Tagging, Telemetry and Marking Measures for Monitoring Fish Populations—A compendium of new and recent science for use in informing technique and decision modalities: Pacific Northwest Aquatic Monitoring Partnership Special Publication 2010-002, chap. 2 (eds Wolf KS, O’Neal JS), pp. 5–46.

Oregon Department of Fish and Wildlife. 2013. Conservation plan for fall Chinook salmon in the Rogue Species Management Unit. Adopted by the Oregon Fish and Wildlife Commission, January 11, 2013.

PFMC (Pacific Fishery Management Council). 2019. Review of 2018 Ocean Salmon Fisheries Stock Assessment and Fishery Evaluation Document for the Pacific Coast Salmon Fishery Management Plan. Pacific Fishery Management Council, 7700 NE Ambassador Place, Suite 101, Portland, OR 97220-1384, USA.

Pacific Fishery Management Council. 2020. Preseason Report I: Stock abundance analysis and environmental assessment part 1 for 2020 ocean salmon fishery regulations. Regulation Identifier Number 0648-BJ48. [www.pcouncil.org](http://www.pcouncil.org)

Satterthwaite, W. H., M. S. Mohr, M. R. O’Farrell, E. C. Anderson, M. A. Banks, S. J. Bates, M. R. Bellinger, L. A. Borgerson, E. D. Crandall, J. C. Garza, B. J. Kormos, P. W. Lawson, and M. L. Palmer-Zwahlen. 2014. Use of genetic stock identification data for comparison of the ocean spatial distribution, size-at-age, and fishery exposure of an untagged stock and its indicator: California Coastal versus Klamath River Chinook salmon. Trans. Am. Fish. Soc. 143:117-133.

Satterthwaite, W. H., J. Ciancio, E. Crandall, M. L. Palmer-Zwahlen, A. M. Grover, M. R. O’Farrell, E. C. Anderson, M. S. Mohr, and J. C. Garza. 2015. Stock composition and ocean spatial distribution inference from California recreational Chinook salmon fisheries using genetic stock identification. Fisheries Research 170: 166-178.

Seeb, L.W., Antonovich, A., Banks, M.A., Beacham, T.D., Bellinger, M.R., Blankenship, S.M., Campbell, M.R., Decovich, N.A., Garza, J.C., Guthrie III, C.M., Lundrigan, T.A., Moran, P., Narum, S.R., Stephenson, J.J., Supernault, K.J., Teel, D.J., Templin, W.D., Wenburg, J.K., Young, S.F., and Smith, C.T. 2007. Development of a standardized DNA database for Chinook salmon. Fisheries 32(11): 540-552.

United States Fish and Wildlife Service (USFWS). 1960. Natural Resources of Northwestern California. Report. Appendix: a preliminary survey of fish and wildlife resources. 104 p.

Zach Larson and Associates. 2013. Operation of dual frequency identification sonar (DIDSON) to monitor adult anadromous fish migrations in the Smith River, California: 2-year pilot study. Submitted to: California Department of Fish and Wildlife and the County of Del Norte.
