# Supplementary material for: Modeling ocean distributions and abundances of natural- and hatchery-origin Chinook salmon stocks with integrated genetic and tagging data
Source: PeerJ. 2023 Nov 28;11:e16487. doi: 10.7717/peerj.16487 (PMC10691356; doi:10.7717/peerj.16487)
Supplement: Supplemental Information 3 [file peerj-11-16487-s003.docx]

Jensen et al. Supplement S3

The supplement provides a discussion of MCMC and model fit diagnostics, as well as supplementary results figures for aspects of the study not addressed in the main text.

Diagnostics – *CWT-only* model

We implemented our statistical model in stan (rstan v2.21.2; Stan Development Team, 2022). We ran 6 chains with a warm up of 400 iterations and 1400 sampling iterations. There were 10 divergent transitions during the sampling phase of the MCMC. Visually, chains appeared to mix well and converge for all parameters. This observed convergence was supported by R-hat values less than 1.01 for all monitored parameters. Estimated effective sample sizes were generally greater than 7,000 but had a minimum of roughly 1,500.

All of the parameters were substantially different from their prior distributions and none of the estimated parameters were estimated to run up against hard boundaries (e.g., no variance parameters were estimated to be very close to 0).

Visual Diagnostics

In total, our fitted model includes 82,542 observations of CWT ocean catch; one for each release group-model age-region-gear combination included. Regions and seasons where there was no fishing effort or no CWT sampling are not included in the model. Out of the 82,542 observations, 11,998 had non-zero observed CWT recoveries. We present predictions and observations for the occurrence (Fig. S3.1) and positive model components (Fig. S3.2). Across all observations of occurrence the model over-predicts the occurrence of CWT (Fig. S3.1) somewhat and this pattern is shared across all stock origins (Fig. S3.3). This pattern is also apparent for the positive component of the model (Figs. S3.2, S3.4), but the magnitude of over-prediction is relatively small.

Additional exploration revealed that this over-prediction is driven primarily by very young fish and very old fish, which are relatively infrequently observed, similar to observed trends in Shelton et al. (2021). For example, we show plots of predicted and observed catch for one stock (SFB) subset by model ages (Figs. S3.5, S3.6; see Table S1.1 for age schedule). For the stocks in our analysis the majority of fish captured are from BY+3, BY+4, and BY+5 (the middle three rows), though the relative amount varies strongly with the stock-specific maturation schedule. In general, the plots of predicted and observed are reasonably good for BY+3 to BY+4 and better during the summer (model ages 6, 10, and 14) and fall (model ages 7, 11, 15) than the spring and winter seasons. Over-prediction occurs most strongly in BY+1 and BY+6 (the first and last rows), suggesting that the model still has difficulty matching the recaptures of the youngest and oldest fish.


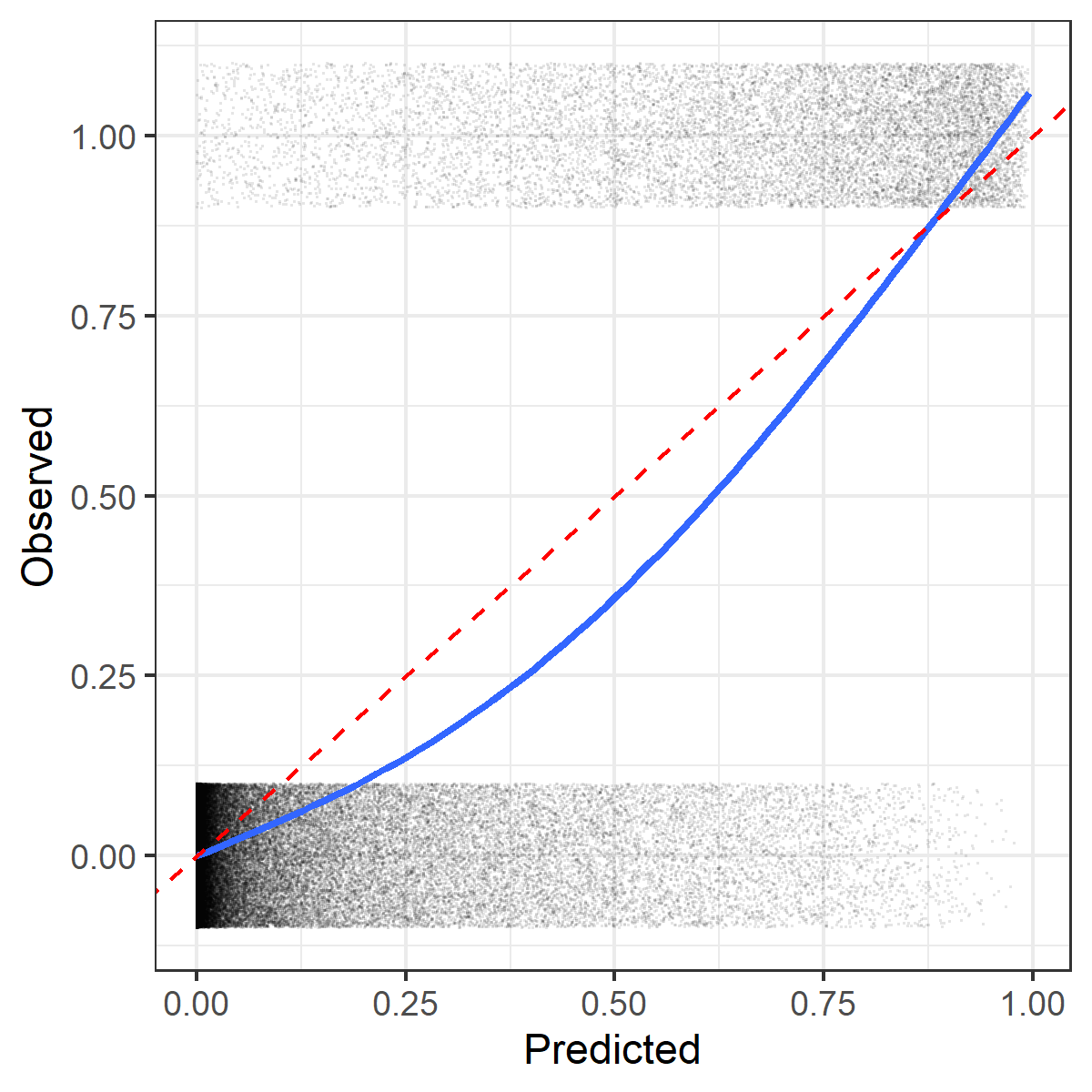


Figure S3.1. Model predictions for the probability of occurrence of CWT against the observed presence (1; observed catch > 0) or absence (0). Each point represents a release group-model age-region-gear combination. Red dashed line shows the 1:1 line, solid line shows a GAM smooth.


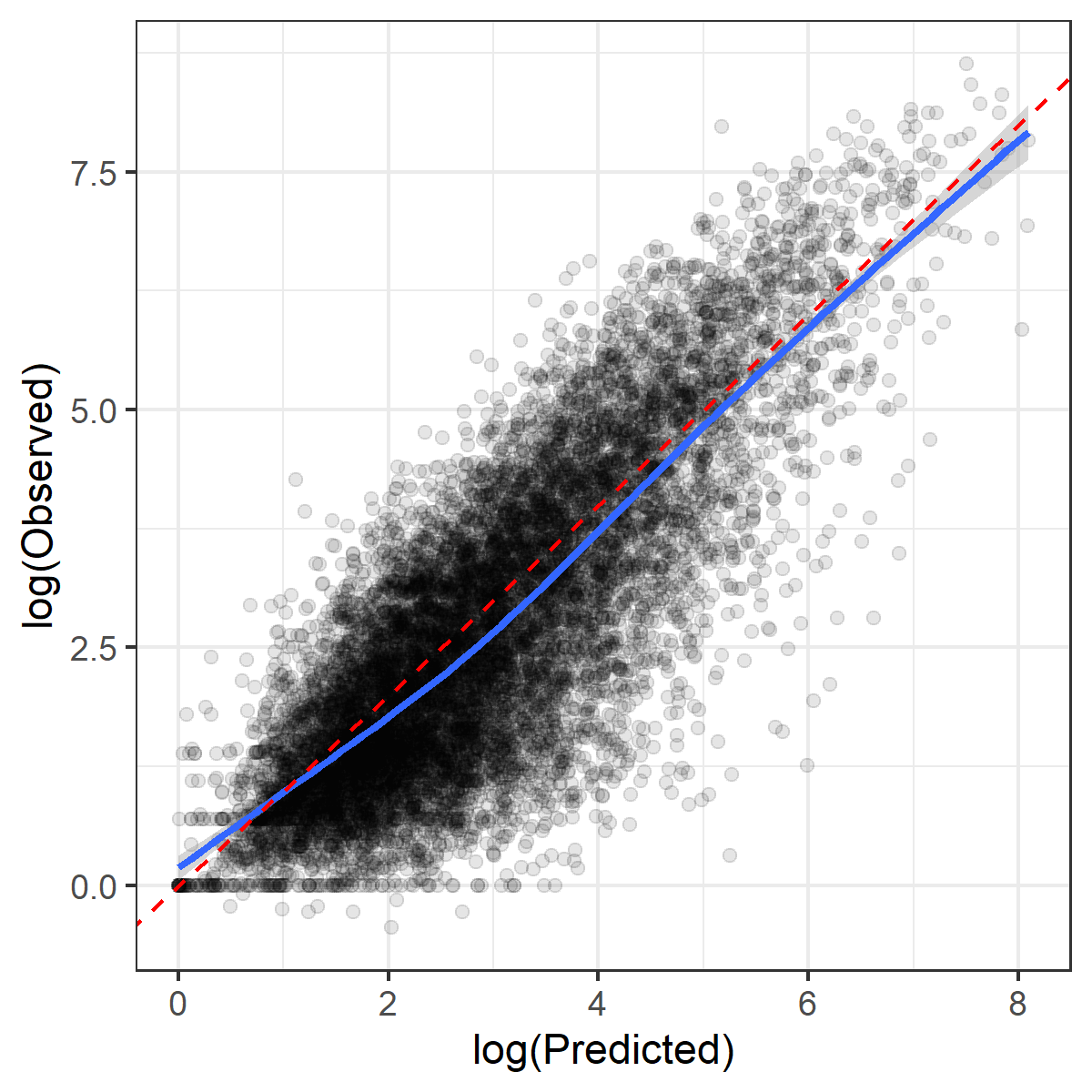


Figure S3.2: Model predictions for positive catch against observed catch of CWT. Each point represents a release group-model age-region combination. Red dashed line shows the 1:1 line, solid line shows a GAM smooth.


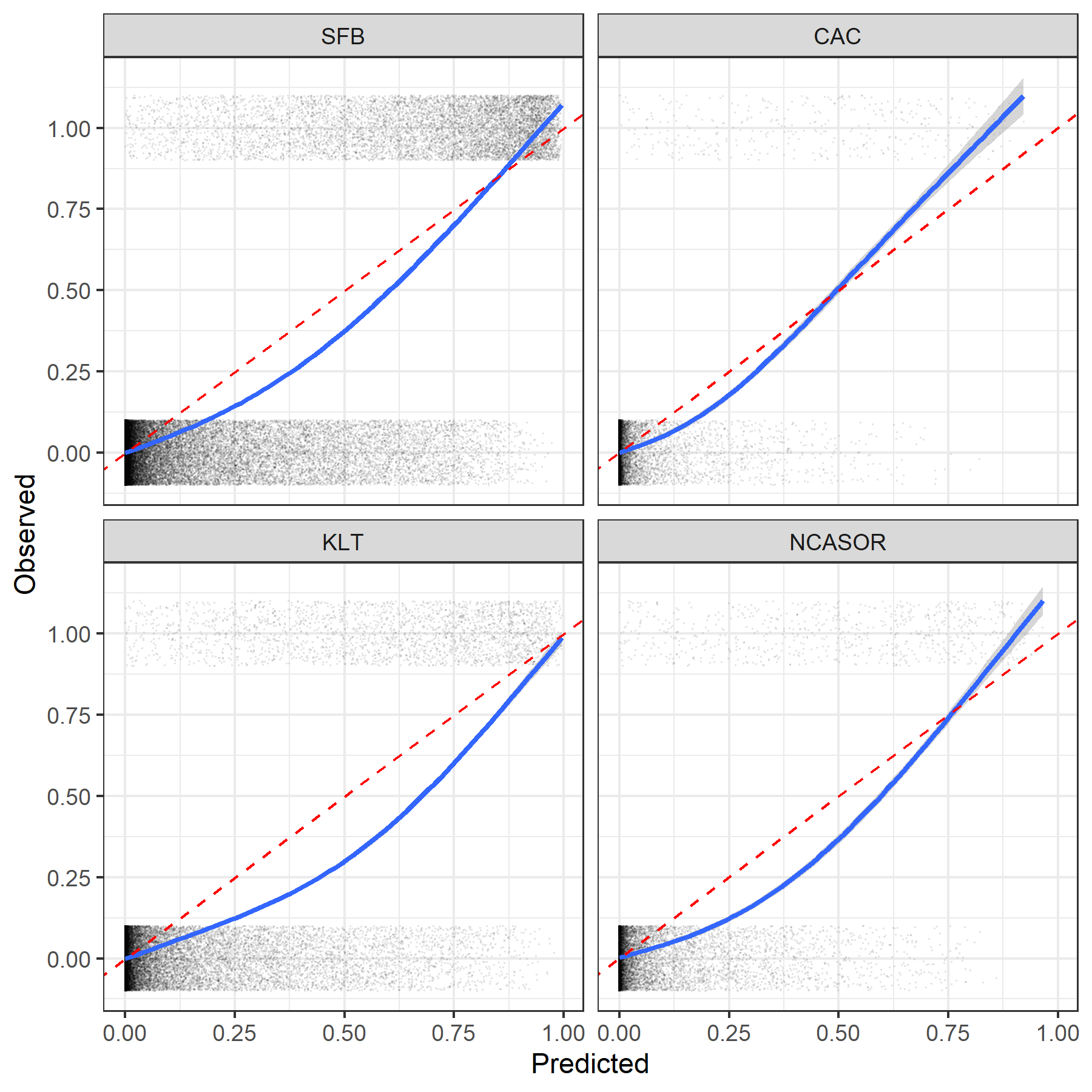


Figure S3.3: Model predictions by origin stock for the probability of occurrence of CWT against the observed presence (1; observed catch > 0) or absence (0). Each point represents a release group-model age-region combination. Red dashed line shows the 1:1 line, solid line shows a GAM smooth.


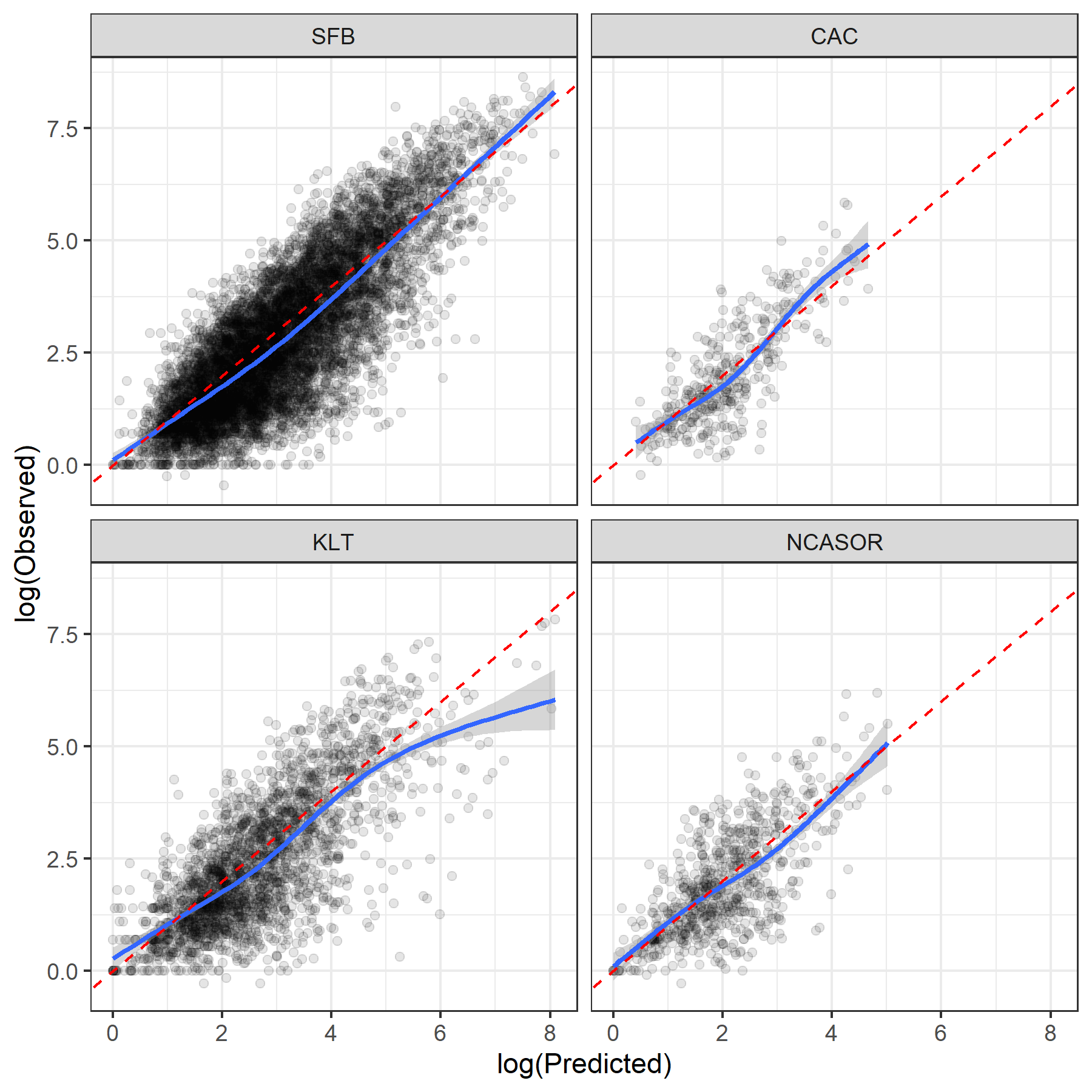


Figure S3.4: Model predictions by origin stocks for positive catch against observed expanded catch of CWT. Each point represents a release group-model age-region combination. Red dashed line shows the 1:1 line, solid line shows a GAM smooth.


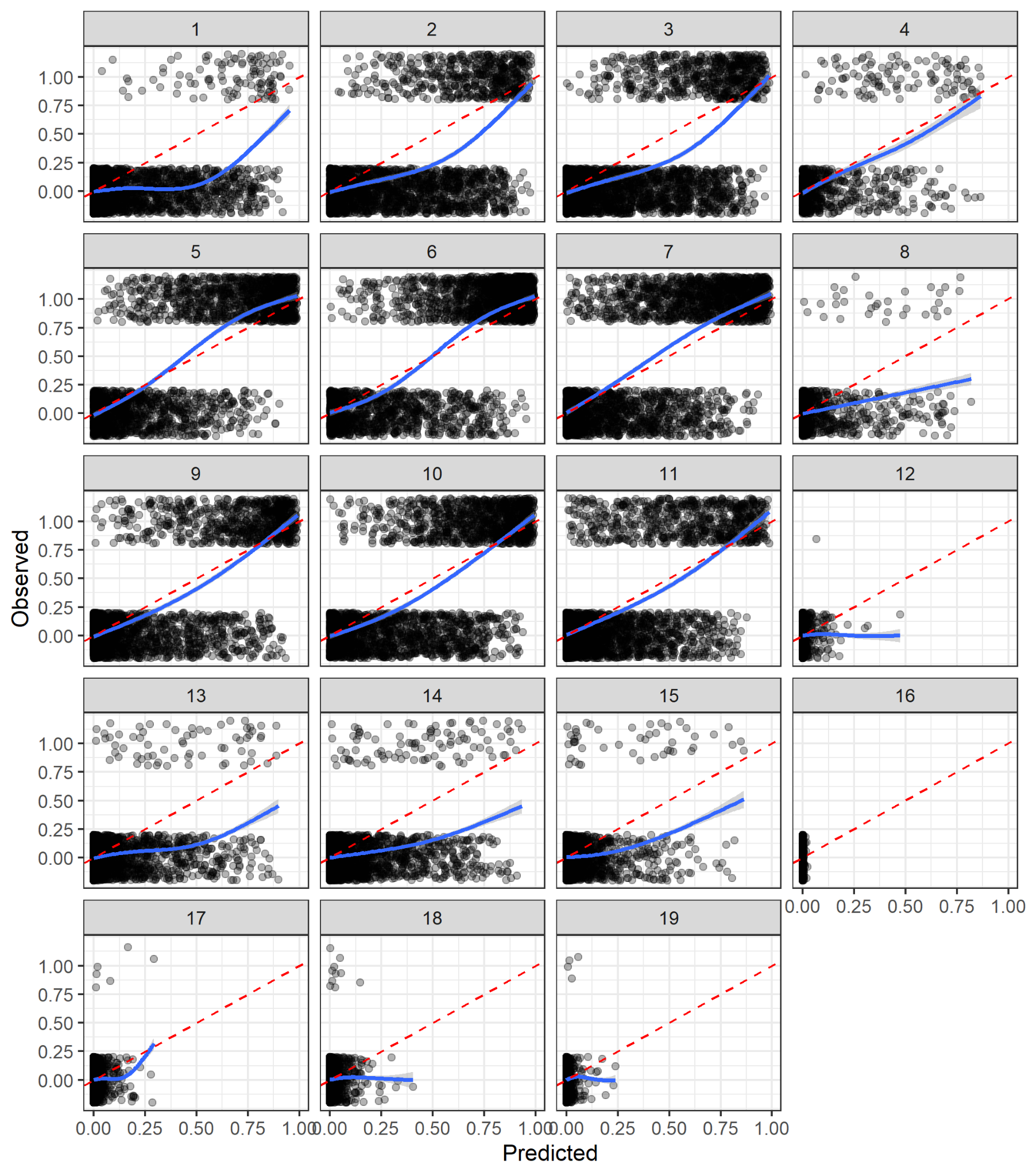


Figure S3.5: Model predictions for the SFB stock for the probability of occurrence of CWT against the observed presence (1; observed catch > 0) or absence (0). Each panel represents a model age, beginning with spring of brood year + 2 (model age = 1; upper left panel) and advancing one season at a time (see Table S1.1). The columns from left to right show spring, summer, fall, and winter seasons. Rows show year brood year + 2 to brood year + 6. Individual points show a release group-model age-region combination. Red dashed line shows the 1:1 line, solid line shows a GAM smooth.


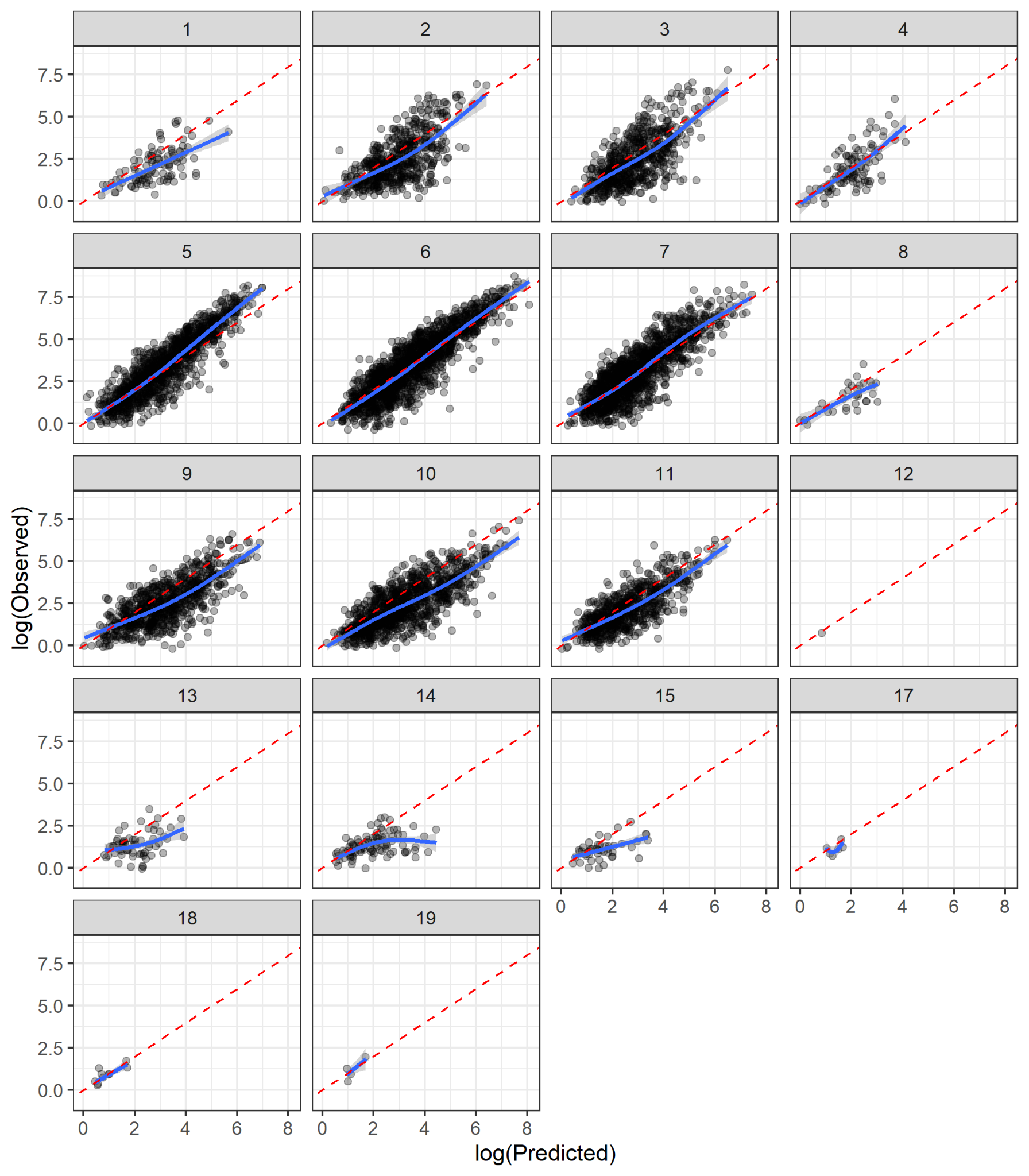


Figure S3.6: Model predictions for the SFB stock for the predicted positive catch of CWT against the observed CWT. Each panel represents a model age beginning with spring of brood year + 2 (model age = 1, upper left panel) and advancing one season at a time (see Table S1.1). The columns from left to right are spring, summer, fall, and winter seasons. Rows show year brood year + 2 to brood year + 6. Individual points show a release group-model age-region combination. Red dashed line shows the 1:1 line, solid line shows a GAM smooth.

Juvenile mortality rates

Similar to results reported in Shelton et al. 2021, we estimate a large amount of variation in early mortality among release groups with an overall estimated hierarchical median of 2.43 (Fig. S3.7). This large variation in juvenile survival rates among release groups has been well documented in a wide variety of previous studies; this is described in detail in Supplement S1 of Shelton et al. (2021).


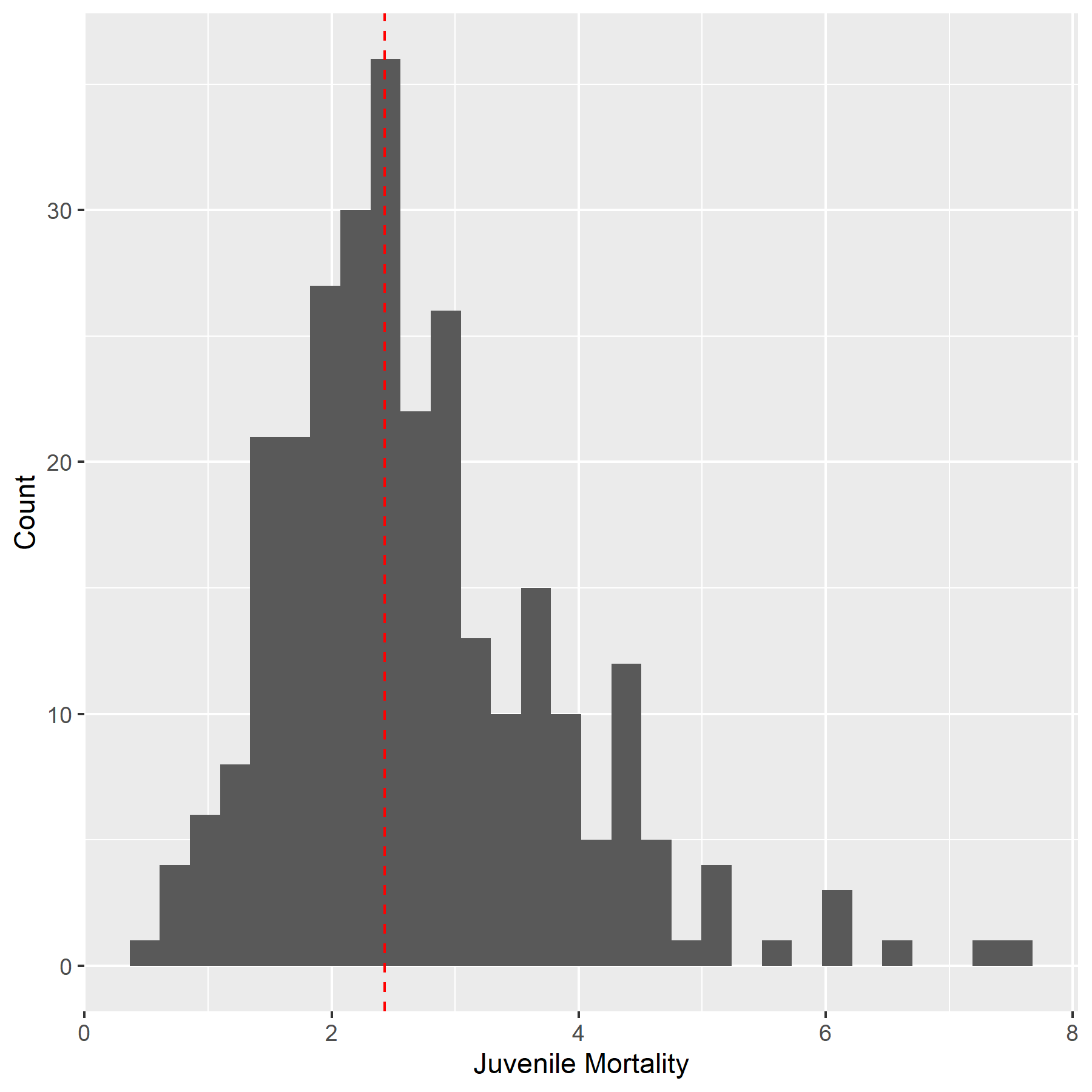


Figure S3.7: The estimated distribution of juvenile mortality rates $\phi$, from release to reaching model age-1, for each of the 284 release groups in the model. The dashed line indicates the estimated hierarchical median value among all the release groups.

Spawning age distributions

We present the estimated spawning age distribution of CWT release groups for one representative brood year for each origin region (Fig. S3.8). The fraction spawning at each age for a single brood year varies substantially among stocks with the SFB stock generally having younger age at maturity than the more northerly origin stocks.


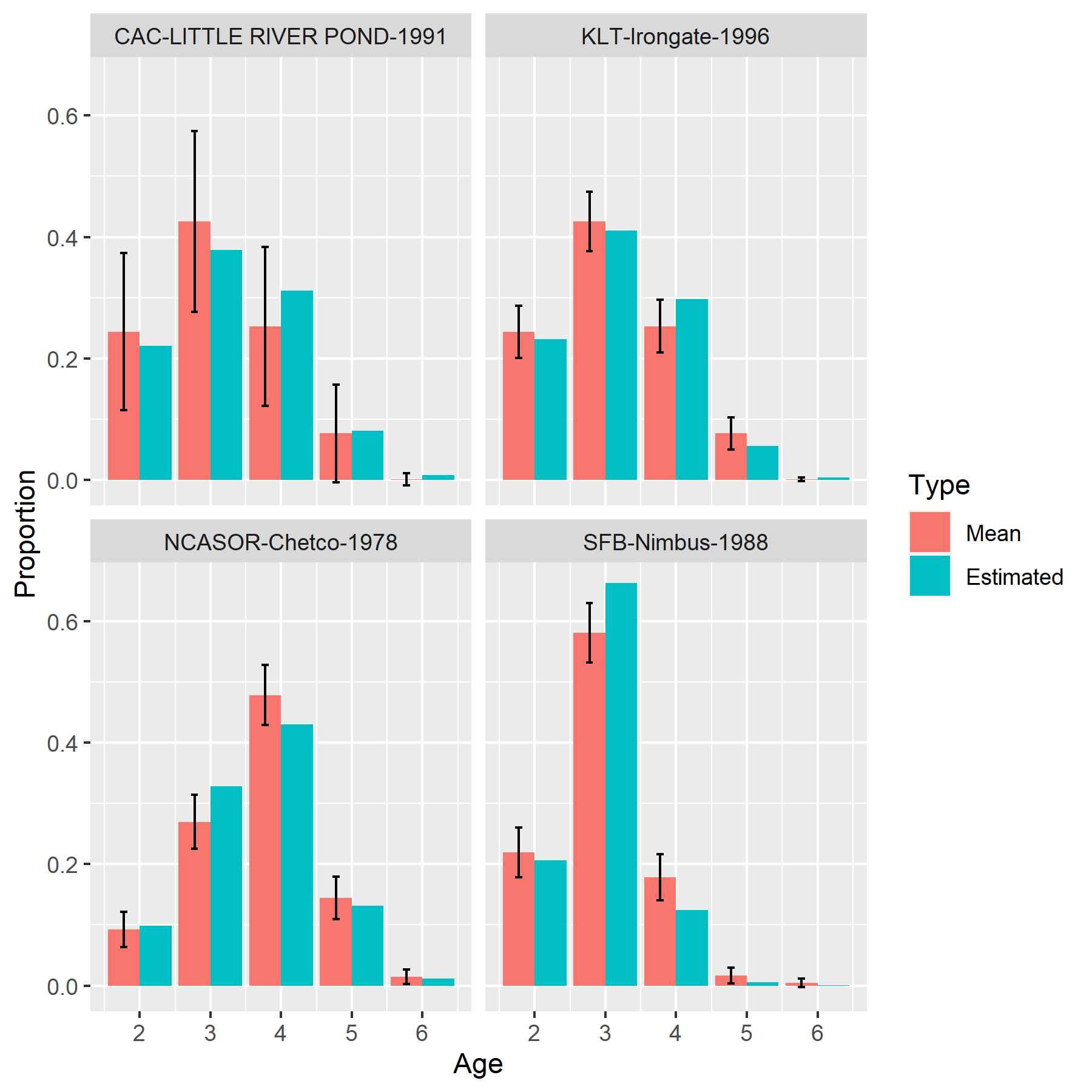


Figure S3.8: Estimated proportion of cohort observed in freshwater. Each panel shows an example cohort from a single brood year from a different stock with the estimated proportional contribution of individuals from each age to freshwater over the course of a life cycle. Red bars indicate long-term mean spawning proportions by age for each stock and blue bars indicate posterior mean estimate of proportions for the release group and year designated in the title. Note that the realized spawning distribution for a single year may differ substantially from the long-term mean spawning age-distribution.

Vulnerability by age

The estimated fraction of Chinook salmon vulnerable for each gear type and model age, associated with a selection of retention sizes, is shown in Figure S3.9.


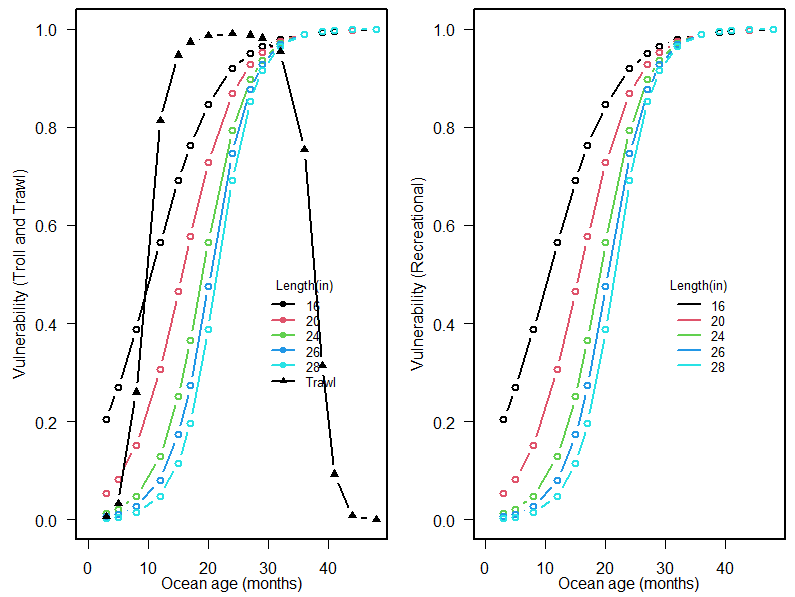


Figure S3.9. Estimated vulnerability to each gear type (left panel: troll and trawl gear types: right panels: recreational) as a function of ocean age. Posterior means are shown. Different colors correspond to vulnerability curves associated with different retention lengths (total length in inches). As most retention lengths are reported in inches, we retain these units.

Catchability

The catchability parameters for each gear type determine the rate at which CWT fish are captured by each fleet (see Supplement S1 for details). We allow catchability to increase through time for the troll, treaty troll, and recreational fleets and allow for spatial variation in the troll fleet (Fig. S3.10). The pelagic trawl fleets do not have time-varying catchabilities. Spatial variation also occurs among recreational fleets due to the different data sources and data types.


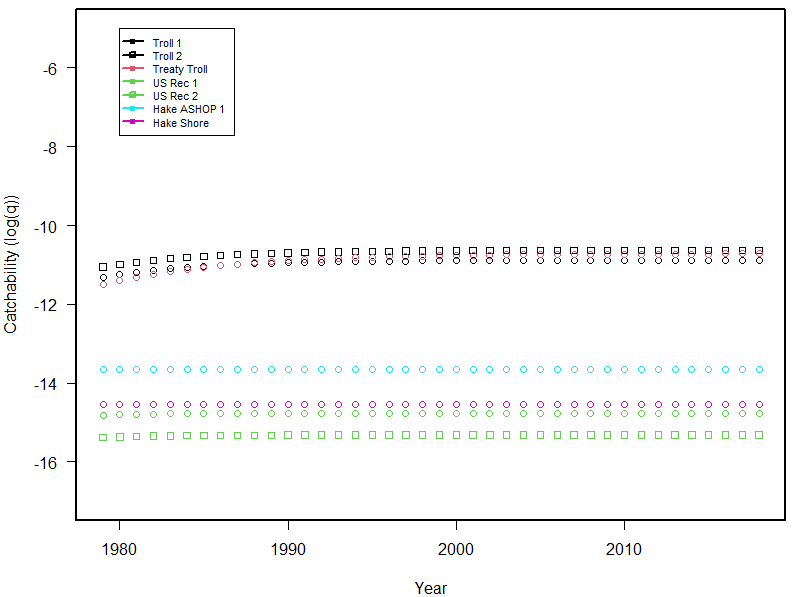


Figure S3.10: Estimates of catchability (log q) for each fleet by year. Point estimates of log q for each fleet are shown.

Diagnostics - *CWT+GSI* model

MCMC Information

We implemented our statistical model in stan (rstan v2.21.2; Stan Development Team, 2022), running 6 chains with a warm up of 400 iterations and 1400 sampling iterations. There were no divergent transitions during the sampling phase of the MCMC. Visually, chains appeared to mix well and converge for most parameters, but some parameters exhibited noticeable divergences among chains. This variability in convergence was indicated by R-hat values with median $\hat{R}=1.01$ and $\hat{R}<1.01$ and $\hat{R}<1.1$ for 48% and 88% of tracked parameters (Table S3.1). Most high R-hat values occurred for model parameters related to estimates of total release group abundance. Estimated effective sample size varied widely among parameters as well, with a median of 1873 but a mean of 4591.

All of the parameters were substantially different from their prior distributions and none of the estimated parameters were estimated to run up against hard boundaries (e.g., no variance parameters were estimated to be very close to 0).

Table S3.1. Summary of R-hat convergence diagnostics for the *CWT+GSI* model.

| Parameter subset | Median | Mean | Max | Proportion R-hat<1.01 | Proportion R-hat<1.1 |
| --- | --- | --- | --- | --- | --- |
| All | 1.01 | 1.21 | 150.46 | 0.48 | 0.88 |
| Mortality | 1.01 | 1.03 | 4.40 | 0.47 | 0.92 |
| CWT release group ocean abundances | 1.00 | 1.02 | 4.02 | 0.78 | 0.97 |
| CWT release group spawning abundances | 1.01 | 1.02 | 1.51 | 0.60 | 0.95 |
| CWT observation parameters | 1.13 | 1.14 | 1.22 | 0 | 0 |
| Spatial distribution | 1.02 | 1.03 | 1.14 | 0.25 | 0.98 |
| GSI total release group ocean abundances | 1.63 | 4.45 | 150.46 | 0 | 0.01 |
| GSI total release group ocean abundances - *Initial abundance only* | 51.59 | 53.54 | 150.46 | 0 | 0 |
| GSI total release group spawning abundances | 1.22 | 1.38 | 3.69 | 0.10 | 0.40 |
| GSI observation parameters | 1.03 | 1.06 | 2.26 | 0.30 | 0.84 |

Visual Diagnostics

Comparisons of observed and predicted CWT ocean catch were highly similar between the *CWT only* and *CWT+GSI* model (i.e., compared to Figs. S3.1-S3.6). Therefore, we only present other diagnostics that exhibited differences between models, as well as new diagnostics corresponding to newly added components for the *CWT+GSI* model.

This model included 139 observations of GSI samples with 10 or more sampled fish per season, year, and region. We present observed and predicted proportions of each of the stocks across all observations, by region, and by fishery type (i.e., recreational versus commercial troll) (Figs. S3.11, S3.12, S3.13). Across all observations, the model tends to under-predict proportions of the predominant SFB stock and over-predict proportions of the rarer stocks. This model bias is particularly pronounced in northern California and in Oregon (i.e., NCA, SOR, NOR) and in the recreational fishery. There are numerous possible explanations for this bias. Differences in fish sampling and data collection methodology between GSI and CWT data could have resulted in different underlying communities being sampled, and thus predicted GSI proportions based on model parameters influenced by CWT data differ from observed GSI proportions. Additionally, greater uncertainty in CAC and NCASOR run sizes may have allowed for inflated estimates of abundance for these stocks, and expected earlier escapement of SFB fish (e.g., Fig. S3.8) based on CWT-tagged fish could have reduced predicted ocean SFB abundances relative to other stocks.

The model includes 68 estimates of total run size for SFB, CAC, KLT, and NCASOR. We present estimated and predicted run sizes over time for each of the stocks (Fig. S3.14). Predicted run sizes for SFB correspond well to independent estimates during the middle of the time series but diverge at the ends. Divergence at the beginning and end of the time series likely occurs due to the inclusion of release groups with incomplete GSI data coverage; for example, the run size abundance for 1998 includes releases from prior years that had multiple ocean rearing years without any GSI data to constrain relative abundances (e.g., a 1994 brood year release group would not have had GSI data for years 1996 or 1997). The model predicts KLT run sizes reasonably well throughout the time series. The model consistently over-predicts run size for both CAC and NCASOR stocks; this occurs likely due to inconsistent implementation of escapement surveys across tributaries and years, resulting in under-estimates of run size used as model inputs.

The model includes 705 estimates of total landings by season, year, region, and fishery type, as provided by the PFMC. We present estimated and predicted landing estimates for the recreational and commercial troll fisheries separately, broken out by spatial region (Figs. S3.15, S3.16). The model consistently under-predicts landings relative to PFMC-based estimates.


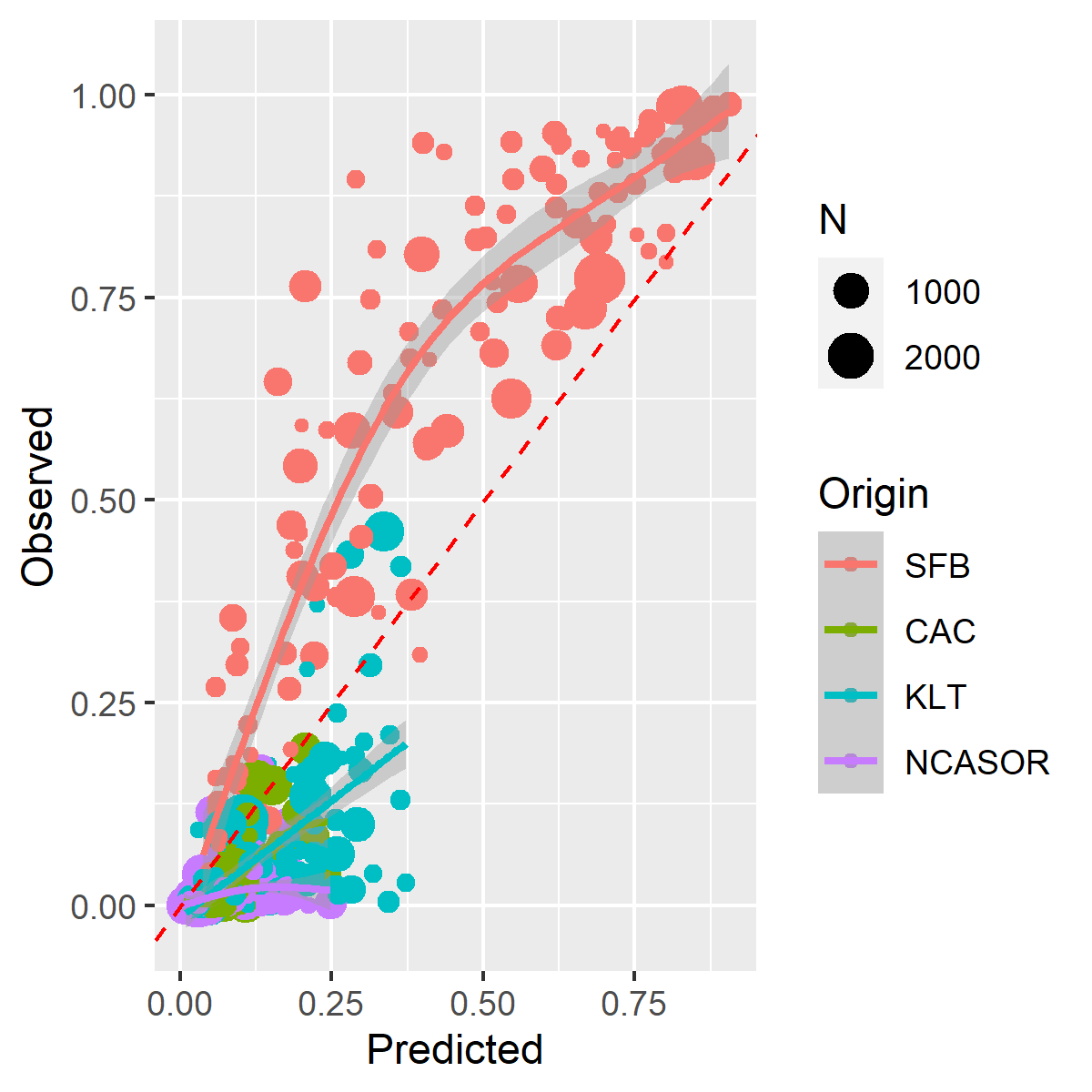


Figure S3.11. Model predictions for the GSI-based stock proportions against observed stock proportions. Sizes of individual data points represent the total number of sampled fish used to generate each observation of stock proportion. Red dashed line shows the 1:1 line, solid lines show a GAM smooth.


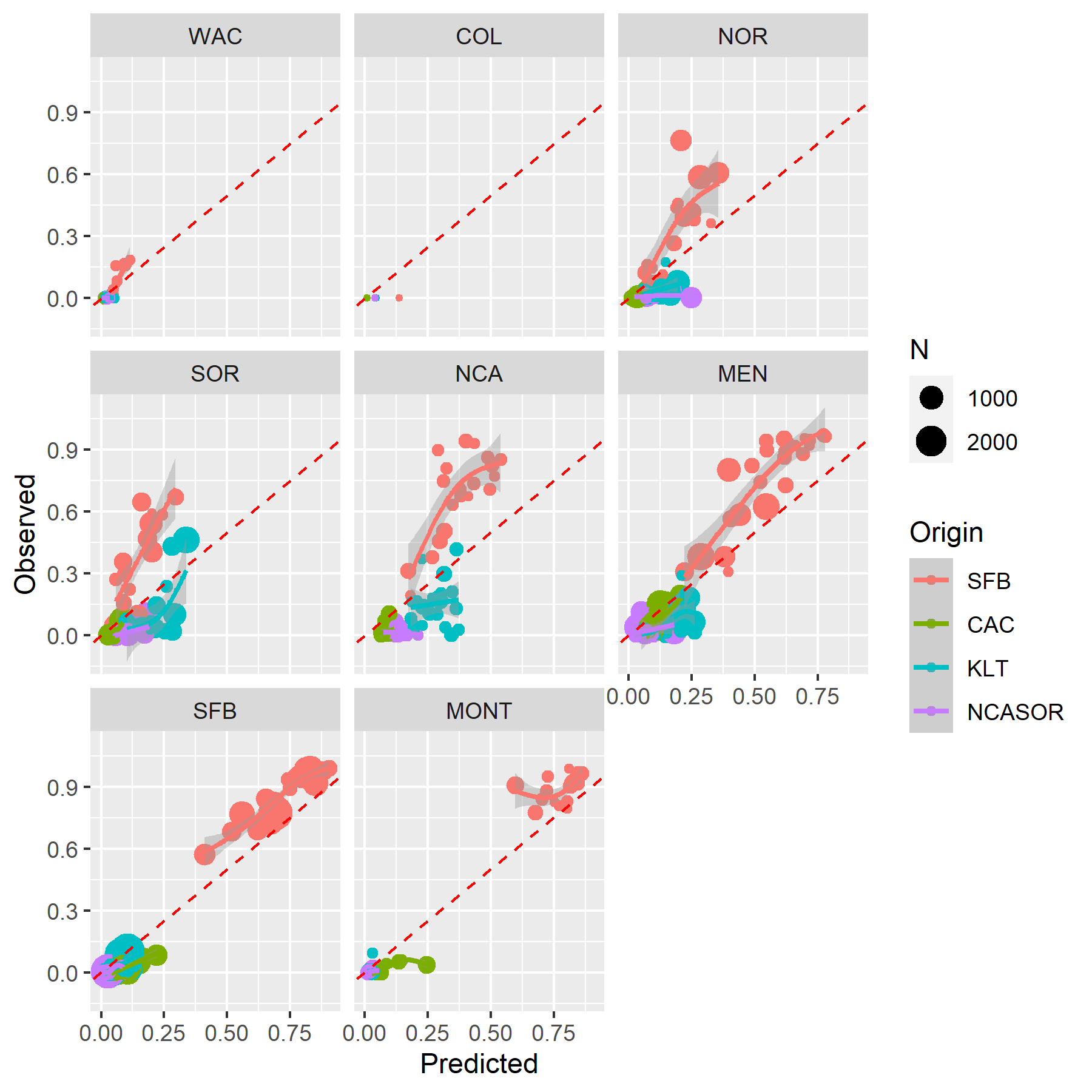


Figure S3.12. Model predictions for the GSI-based stock proportions against observed stock proportions, faceted by spatial region. Sizes of individual data points represent the total number of sampled fish used to generate each observation of stock proportion. Red dashed line shows the 1:1 line, solid lines show a GAM smooth.


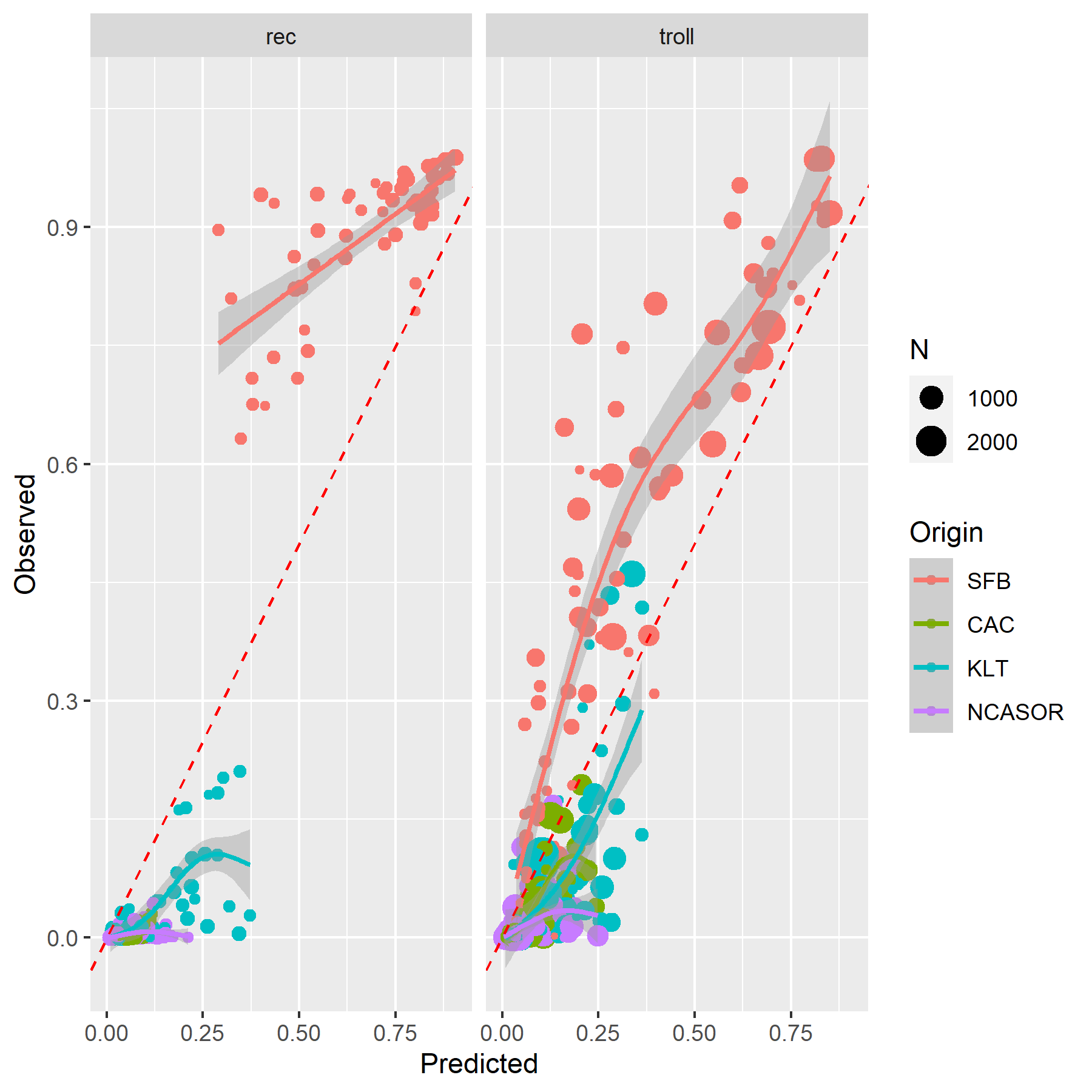


Figure S3.13. Model predictions for the GSI-based stock proportions against observed stock proportions, faceted by fishery type (i.e., recreational versus commercial troll). Sizes of individual data points represent the total number of sampled fish used to generate each observation of stock proportion. Red dashed line shows the 1:1 line, solid lines show a GAM smooth.


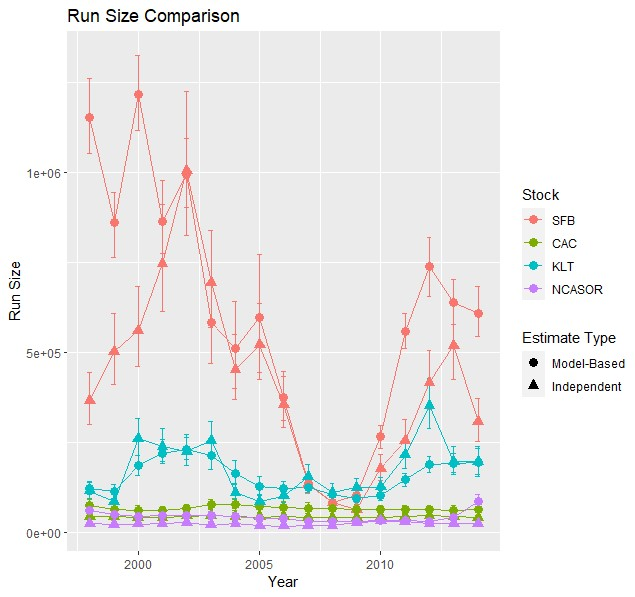


Figure S3.14. Model predictions for stock-specific run sizes against independent estimates generated from available survey data. Error bars for model predictions represent 95% credible intervals and error bars for independent estimates represent simulated 95% confidence intervals based on specified lognormal standard deviations.


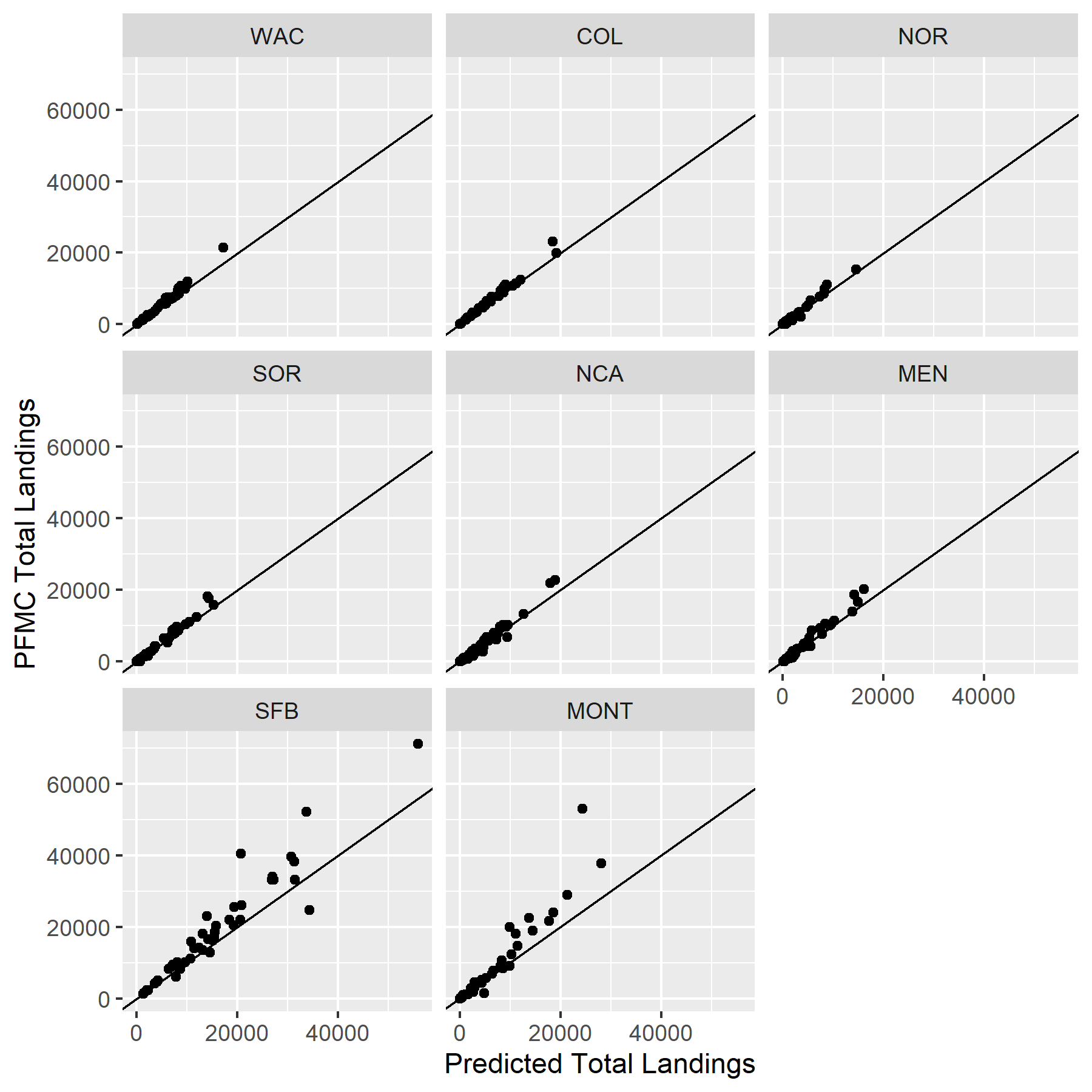


Figure S3.15. Model predictions for recreational fishery total landings by season and year against landings estimates provided by PFMC, broken out by spatial region. Solid lines represent a 1:1 relationship.


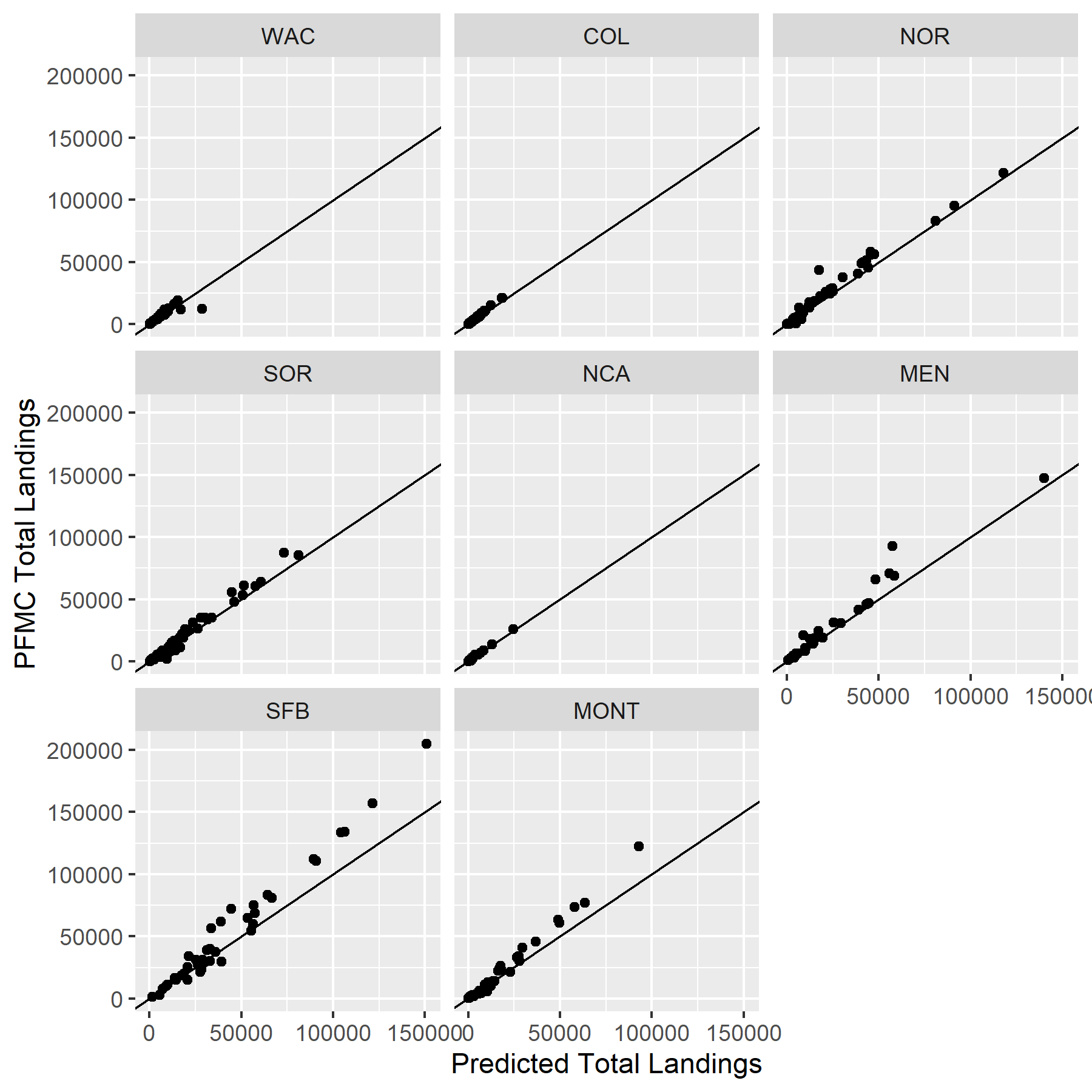


Figure S3.16. Model predictions for commercial troll fishery total landings by season and year against landings estimates provided by PFMC, broken out by spatial region. Solid lines represent a 1:1 relationship.

GSI Overdispersion Parameter

We estimated the overdispersion parameter for GSI mixture observations to be 0.032 (95% credible interval: 0.029 - 0.035) (Fig. S3.17). Overdispersion values between 0 and 1 indicate overdispersion and act to decrease the effective sample size of GSI observations; the low mean estimate of 0.032 indicates the model is putting relatively little weight on the GSI observations compared to other likelihood components in the model.


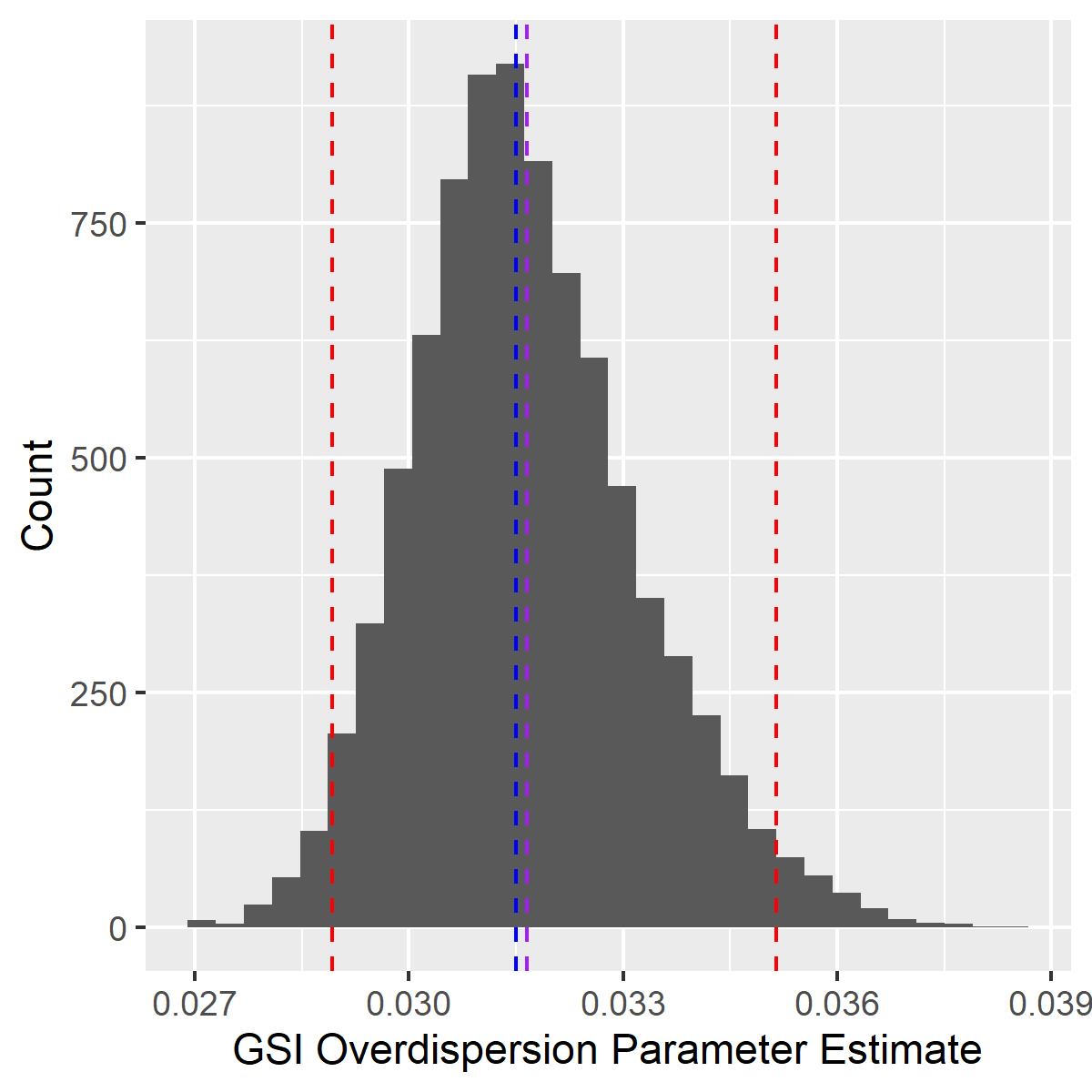


Figure S3.17. Parameter estimates for the GSI overdispersion term. Dashed blue, purple, and red lines indicate the median, mean, and 95% credible intervals, respectively.

Juvenile mortality rates

We again estimate a large amount of variation in early mortality among release groups, with an overall estimated hierarchical median of 4.16 (Fig. S3.18). This translates to a lower juvenile survival rate for the joint CWT and GSI model compared to the no GSI model.


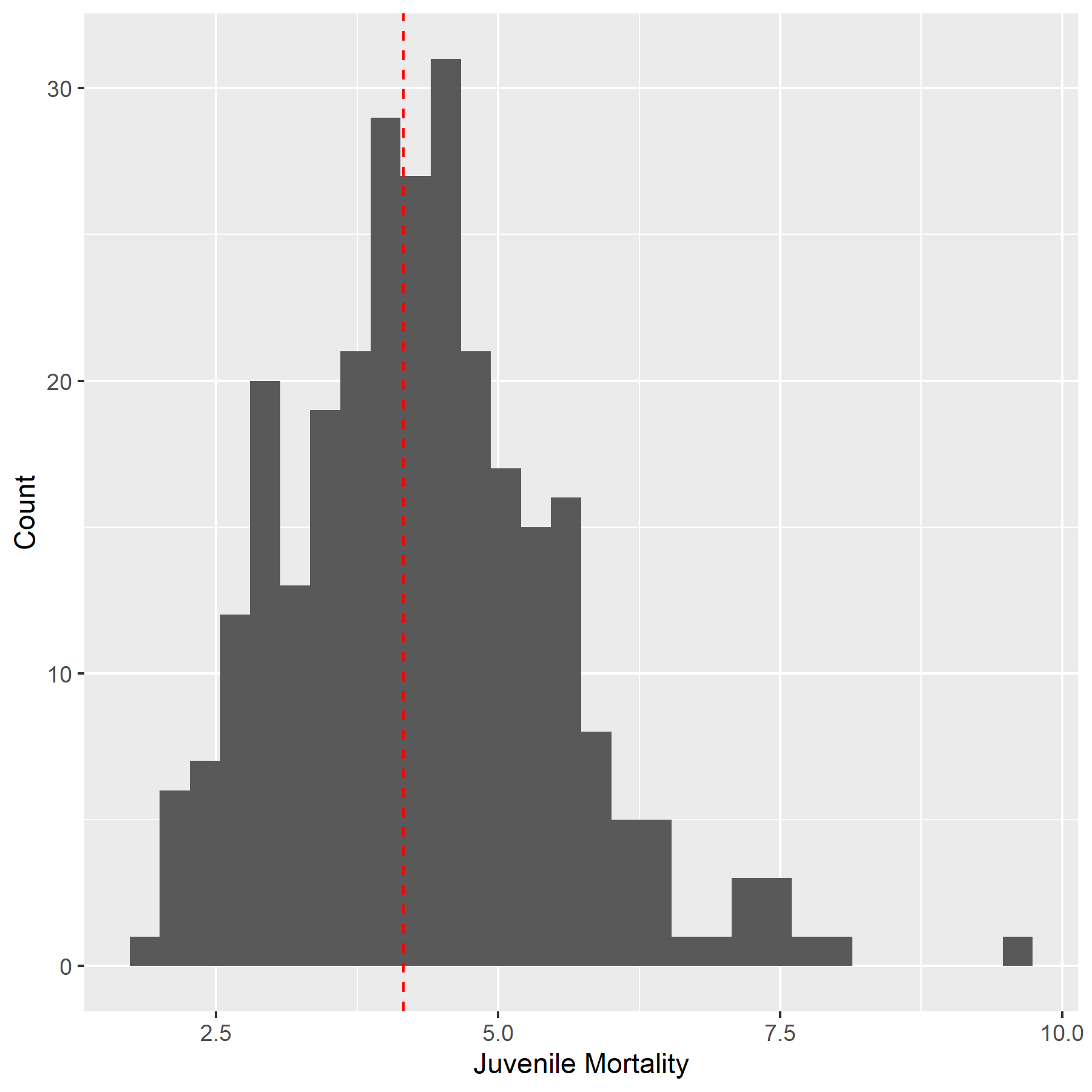


Figure S3.18: The estimated distribution of juvenile mortality rates $\phi$, from release to reaching model age-1, for each of the 284 release groups in the model. The dashed line indicates the estimated hierarchical median value among all the release groups.

Spawning age distributions

We present estimated spawning age distributions of total release groups for one representative brood year for each origin region (Fig. S3.19). Similar to results for CWT release groups from the no GSI model, the fraction spawning at each age for a single brood year varies substantially among stocks with the SFB stock generally having younger age at maturity than the more northerly origin stocks.


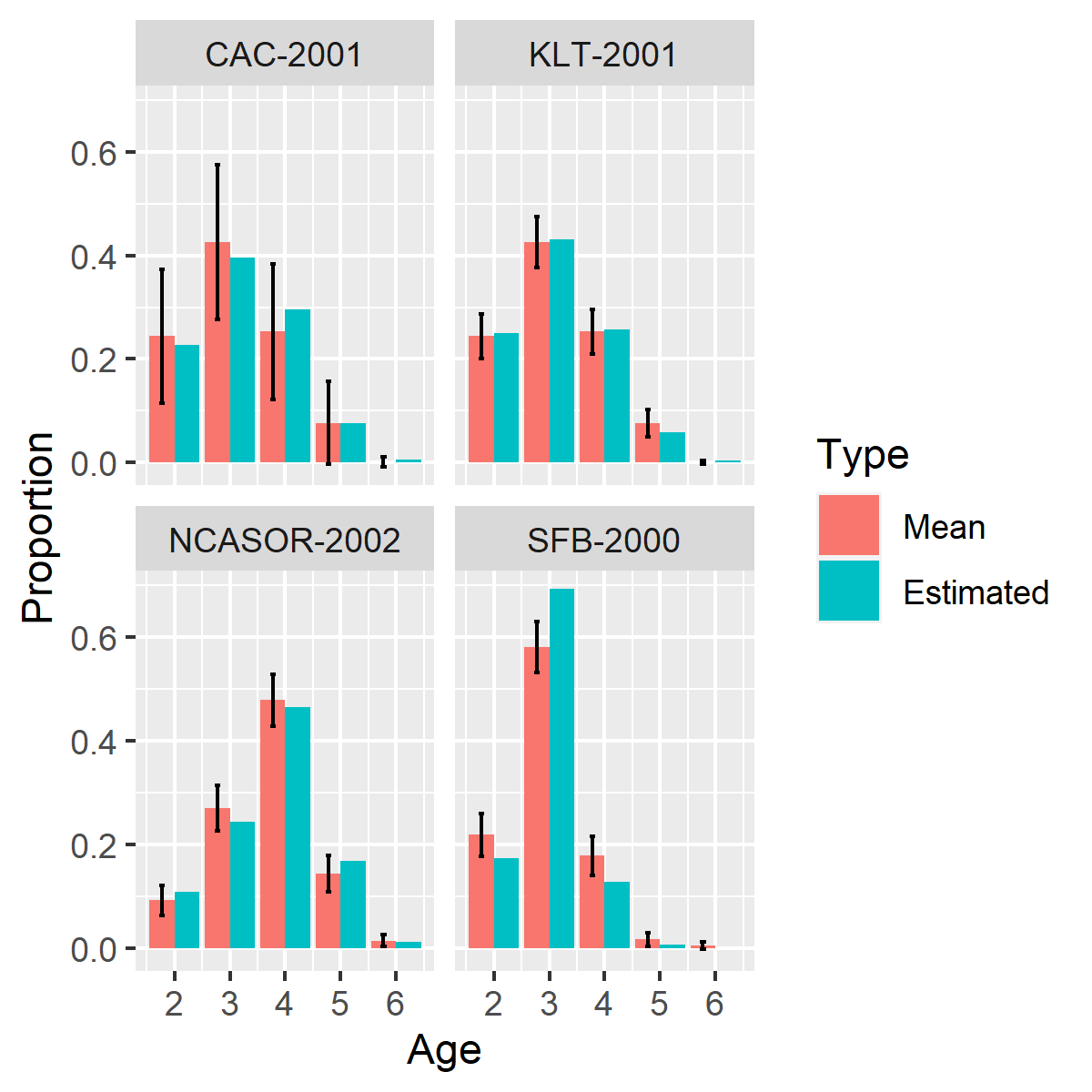


Figure S3.19: Estimated proportion of cohort observed in freshwater. Each panel shows an example cohort from a single brood year from a different stock with the estimated proportional contribution of individuals from each age to freshwater over the course of a life cycle. Red bars indicate long-term mean spawning proportions by age for each stock and blue bars indicate posterior mean estimate of proportions for the release group and year designated in the title. \Note that the realized spawning distribution for a single year may differ substantially from the long-term mean spawning age-distribution.

Vulnerability by age

The estimated fraction of Chinook salmon vulnerable for each gear type and model age, associated with a selection of retention sizes, is shown in Figure S3.20.


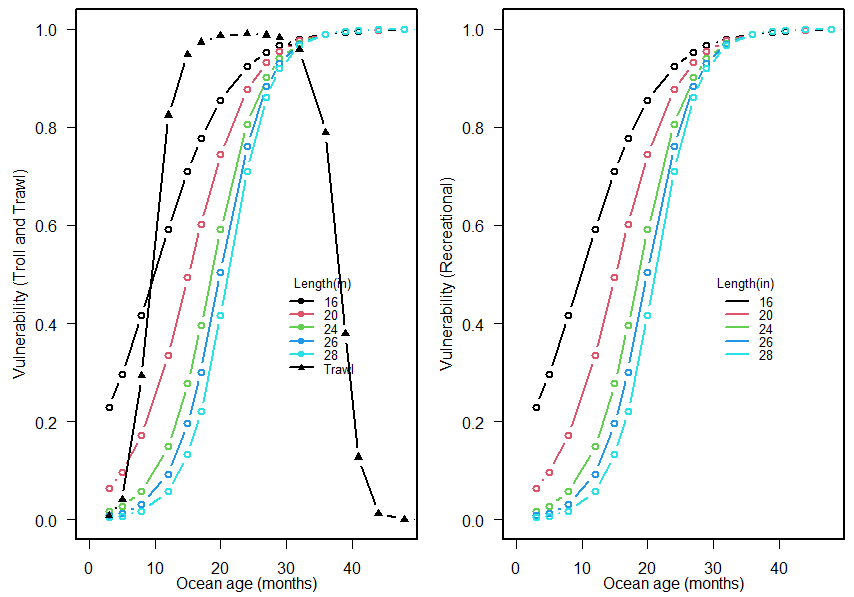


Figure S3.20. Estimated vulnerability to each gear type (left panel: troll and trawl gear types: right panel: recreational) as a function of ocean age. Posterior means are shown. Different colors correspond to vulnerability curves associated with different retention lengths (total length in inches). As most retention lengths are reported in inches, we retain these units.

Catchability

Trends in catchability parameters for each gear type are similar to those reported for the no GSI model (Fig. S3.21). However, the absolute catchability values are higher, resulting in higher expected fishing mortality rates.


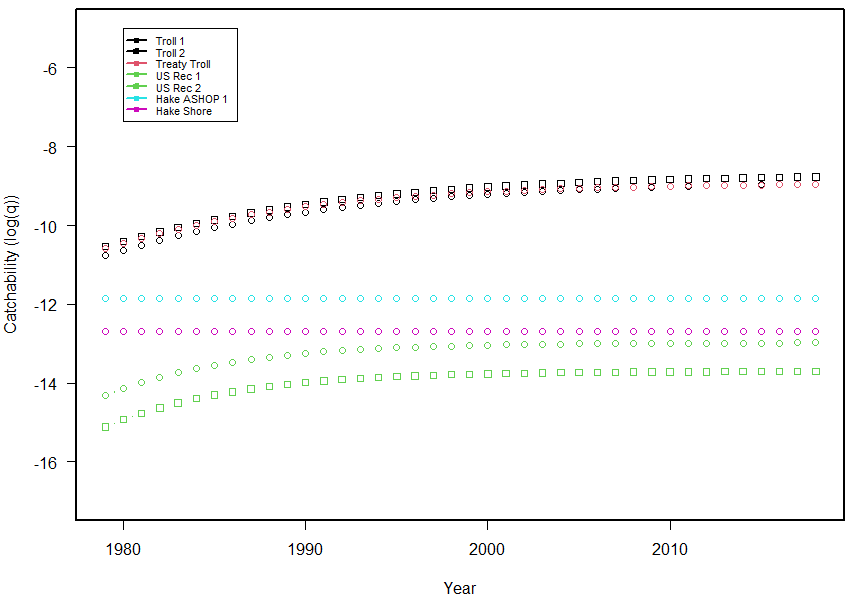


Figure S3.21: Estimates of catchability (log q) for each fleet by year. Point estimates of log q for each fleet are shown.

Diagnostics - *CWT+GSI+Age* Model

MCMC Information

We implemented our statistical model in stan (rstan v2.21.2; Stan Development Team, 2022), running 6 chains with a warm up of 400 iterations and 1400 sampling iterations. There were no divergent transitions during the sampling phase of the MCMC. Visually, chains appeared to mix well and converge for most parameters, but some parameters exhibited noticeable divergences among chains. This variability in convergence was indicated by median $\hat{R}=1.01$ and $\hat{R}<1.01$ and $\hat{R}<1.1$ for 49% and 90% of tracked parameters (Table S3.2). Addition of age structure constraints markedly improved convergence for estimates of total release group abundances, as well as other parameters, but very high R-hat values again occurred for model parameters related to estimates of initial total release group abundance. Estimated effective sample size varied widely among parameters as well, with a median of 2658 but a mean of 4559.

All of the parameters were substantially different from their prior distributions and none of the estimated parameters were estimated to run up against hard boundaries (e.g., no variance parameters were estimated to be very close to 0).

Table S3.2. Summary of R-hat convergence diagnostics for the *CWT+GSI+Age* model.

| Parameter subset | Median | Mean | Max | Proportion R-hat<1.01 | Proportion R-hat<1.1 |
| --- | --- | --- | --- | --- | --- |
| All | 1.01 | 1.19 | 179.58 | 0.49 | 0.90 |
| Mortality | 1.01 | 1.04 | 6.56 | 0.40 | 0.91 |
| CWT release group ocean abundances | 1.00 | 1.03 | 7.19 | 0.77 | 0.97 |
| CWT release group spawning abundances | 1.01 | 1.02 | 1.53 | 0.61 | 0.97 |
| CWT observation parameters | 1.07 | 1.09 | 1.16 | 0 | 0.625 |
| Spatial distribution | 1.01 | 1.02 | 1.12 | 0.41 | 0.99 |
| GSI total release group ocean abundances | 1.22 | 4.16 | 179.49 | 0.01 | 0.29 |
| GSI total release group ocean abundances - *Initial abundance only* | 47.65 | 55.28 | 179.58 | 0 | 0 |
| GSI total release group spawning abundances | 1.08 | 1.18 | 2.96 | 0.13 | 0.55 |
| GSI observation parameters | 1.01 | 1.02 | 2.03 | 0.42 | 0.97 |

Visual Diagnostics

We examined visual diagnostics, as well as analysis of juvenile mortality rates, spawning age distributions, vulnerability by age, and catchability, following the report structure from the CWT+GSI model with no synthetic age structure. We did not observe any obvious differences between the two sets of assessments, with the exception of the estimated overdispersion term, and therefore do not repeat redundant results again here.

Wo do present, however, visual diagnostics comparing the pre-specified age proportions to those estimated in model fitting (Fig. S3.22). Model estimates of age structure largely matched specified age proportions. We do note the model tended to under-estimate the contribution of younger age classes and over-estimate the contribution of older age classes, relative to our somewhat empirically informed, pre-specified age proportions.


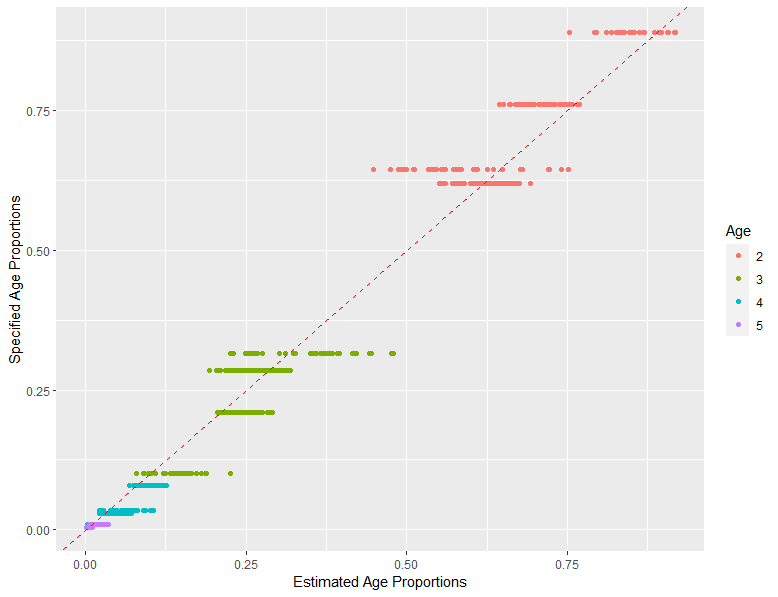


Figure S3.22. Model predictions for the estimated proportions of different age classes plotted against specified age proportions. There are multiple specified proportions for a given age because we assigned the SFB stock a different age structure than other stocks and compared different numbers of age classes depending on the season (ages 2-5 in spring/summer, 3-5 in fall/winter). The red dashed line shows the 1:1 line.

GSI Overdispersion Parameter

We estimated the mean overdispersion parameter for GSI mixture observations to be 0.029 (95% credible interval: 0.027 - 0.032) (Fig. S3.23). The overall distribution of parameter estimates is slightly less in this model with synthetic age structure, compared to the model without it, suggesting slightly less weight is applied to GSI observations in this model.


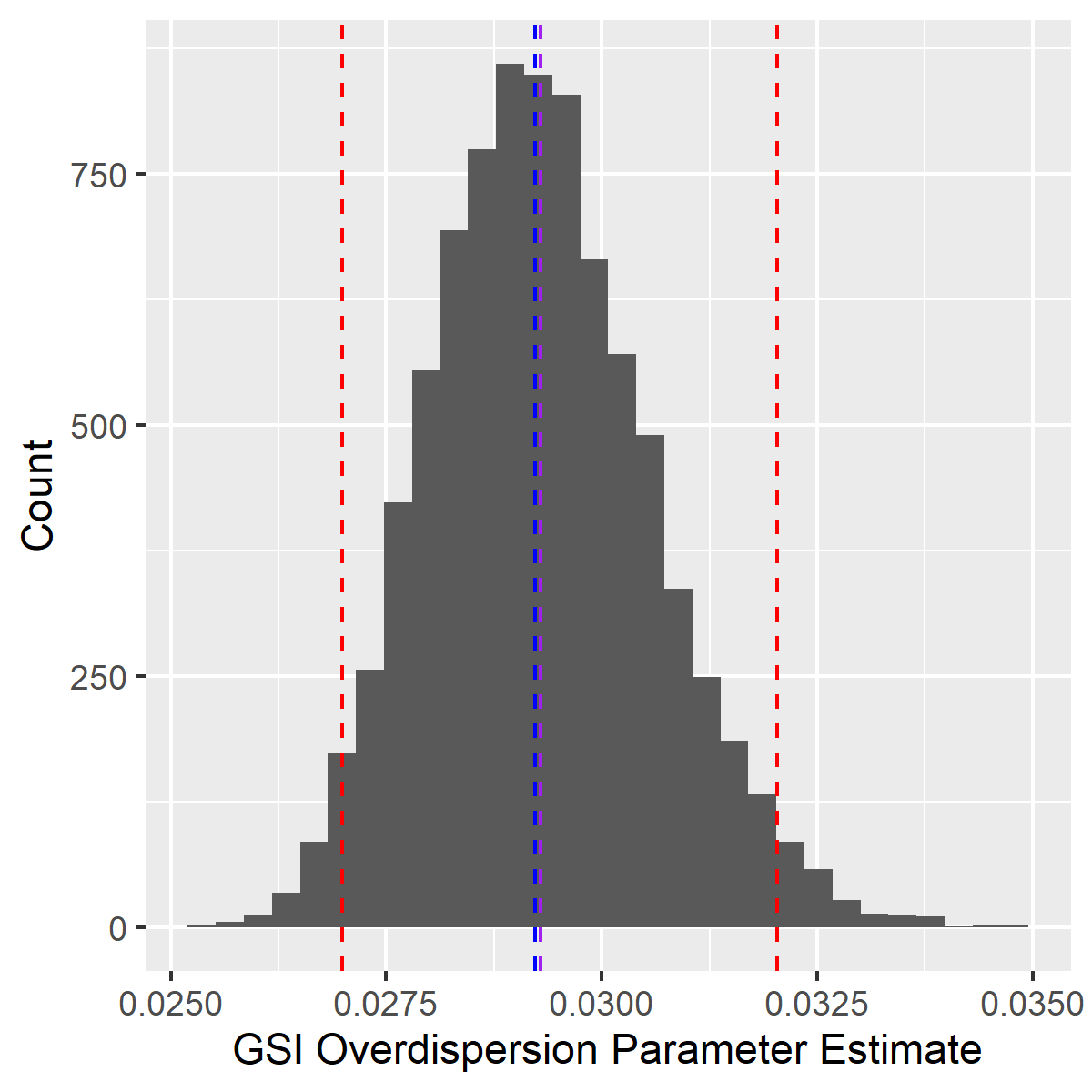


Figure S3.23. Parameter estimates for the GSI overdispersion term. Dashed blue, purple, and red lines indicate the median, mean, and 95% credible intervals, respectively.

Direct Comparison of Convergence for GSI+CWT Models With and Without Synthetic Age Structure

We present a comparison of model convergence, as indicated by R-hat values, for estimates of total release group abundances for the CWT+GSI models with and without synthetic age structure. We observed that adding the synthetic age structure improved model convergence for estimates of post-season abundances of total release groups (i.e., after natural and fishing mortality are applied), but the observed improvement does not in result in satisfactory convergence based on an upper R-hat threshold of 1.01 (Figure S3.24). Adding synthetic age structure did not improve model convergence for estimates of initial abundance of total release groups (i.e., abundance at the start of the first tracked season, before any fishing or natural mortality has been applied) (Figure S3.25). The extreme R-hat values for these parameters suggests our lack of information on starting release group abundance is particularly problematic for these initial estimates of abundance.


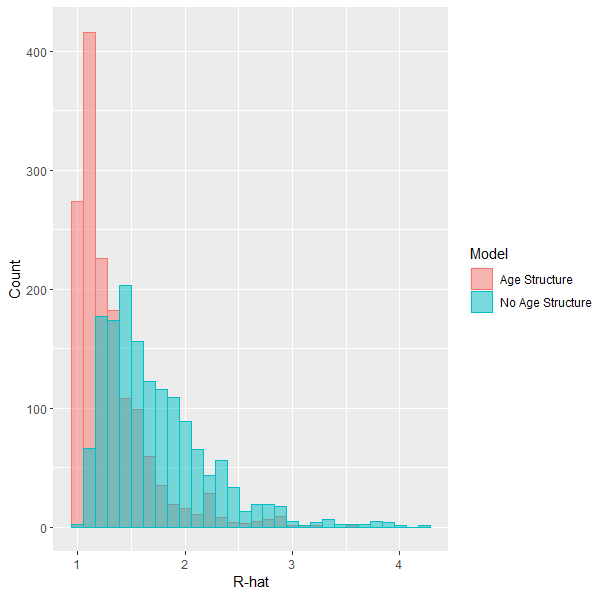


Figure S3.24. Summary of R-hat values for all log-transformed abundance estimates for total release groups at the end of each season, after natural and fishing mortality have been applied, for the CWT+GSI models with and without synthetic age structure.


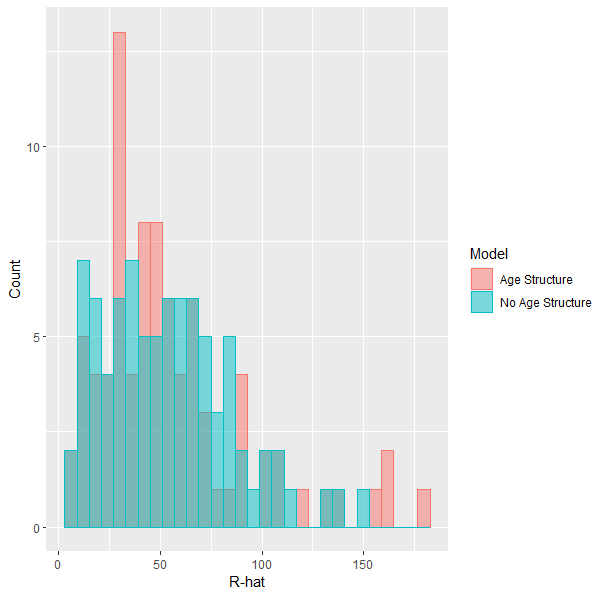


Figure S3.25. Summary of R-hat values for log-transformed estimates of initial abundance for total release groups, at the beginning of the first tracked season, for the CWT+GSI models with and without synthetic age structure.

Comparison of Estimated Spatial Distribution for GSI+CWT Models With and Without Synthetic Age Structure

We compare estimated seasonal spatial distributions of the four stocks for the GSI+CWT models with and without synthetic age structure (Figure S3.26). We observe little to no difference in spatial distribution between models, indicating that convergence (or lack thereof) of abundance parameters does not affect our estimates of spatial distribution in the GSI+CWT models.


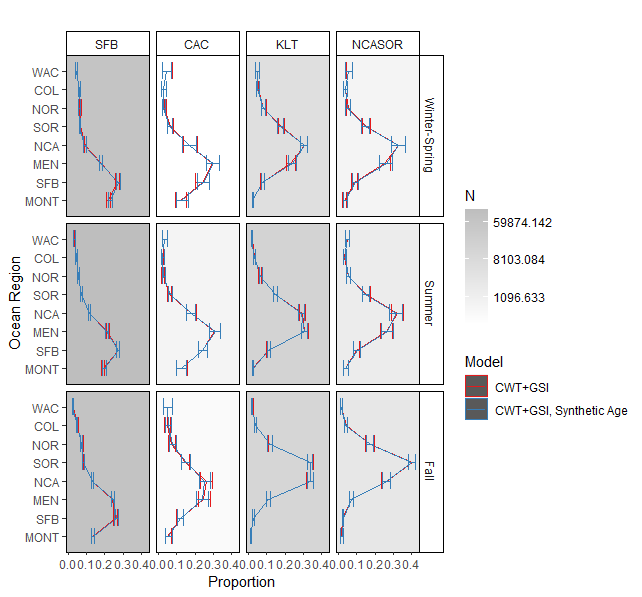


Figure S3.26. Estimated spatial distributions of the four tracked stocks by season. Shades of gray in each facet represent the total number of recovered CWTs that contributed to estimates of spatial distribution. Error bars represent 95% credible intervals.

Comparison of Estimated Stock Abundances for GSI+CWT Models With and Without Synthetic Age Structure

We present estimated regional and seasonal stock abundances for 2002 and 2009 stocks for the *GSI+CWT+Age* model, for comparison to estimated stock abundances for the *GSI+CWT* model in Figure 5 (Figure S3.27). We observe little to no difference in estimated stock abundances between models.


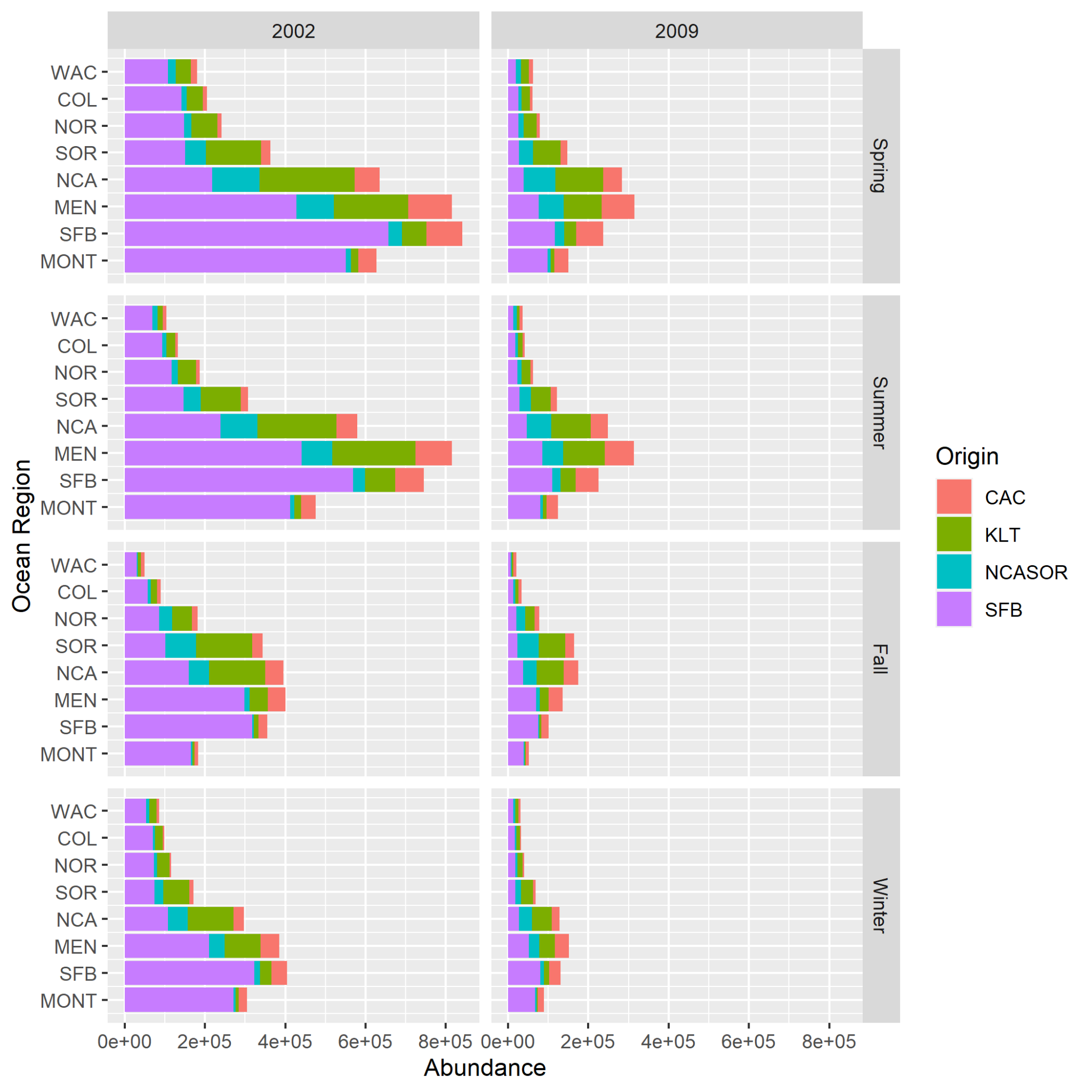


Figure S3.27. Estimated stock abundances and proportional contributions by season, year, and region for the *CWT+GSI+Age* model. We selected 2002 and 2009 to highlight contrast in total abundance and stock-specific proportional contributions to abundance.

**Citations**

Stan Development Team (2022). “RStan: the R interface to Stan.” R package version 2.21.2, https://mc-stan.org/
